# Phage-assisted continuous evolution of proteases with altered substrate specificity

**DOI:** 10.1038/s41467-017-01055-9

**Published:** 2017-10-16

**Authors:** Michael S. Packer, Holly A. Rees, David R. Liu

**Affiliations:** 1 Department of Chemistry and Chemical Biology, Harvard University, 12 Oxford Street, Cambridge, MA 02138 USA; 2 Graduate Program in Biophysics Program, Harvard University, 240 Longwood Avenue, Boston, MA 02115 USA; 3 Broad Institute of MIT and Harvard, 75 Ames Street, Cambridge, MA 02142 USA; 4 Howard Hughes Medical Institute, Harvard University, 12 Oxford Street, Cambridge, MA 02138 USA

## Abstract

Here we perform phage-assisted continuous evolution (PACE) of TEV protease, which canonically cleaves ENLYFQS, to cleave a very different target sequence, HPLVGHM, that is present in human IL-23. A protease emerging from ∼2500 generations of PACE contains 20 non-silent mutations, cleaves human IL-23 at the target peptide bond, and when pre-mixed with IL-23 in primary cultures of murine splenocytes inhibits IL-23-mediated immune signaling. We characterize the substrate specificity of this evolved enzyme, revealing shifted and broadened specificity changes at the six positions in which the target amino acid sequence differed. Mutational dissection and additional protease specificity profiling reveal the molecular basis of some of these changes. This work establishes the capability of changing the substrate specificity of a protease at many positions in a practical time scale and provides a foundation for the development of custom proteases that catalytically alter or destroy target proteins for biotechnological and therapeutic applications.

## Abstract (web summary)

Proteases are promising therapeutics to treat diseases such as hemophilia which are due to endogenous protease deficiency. Here the authors use phage-assisted continuous evolution to evolve a variant TEV protease with altered target peptide sequence specificities.

## Introduction

Proteases are ubiquitous regulators of protein function in all domains of life and represent approximately one percent of known protein sequences^1^. Substrate-specific proteases have proven useful as research tools and as therapeutics that supplement a natural protease deficiency to treat diseases such as hemophilia, or that simply perform their native functions such as the case of botulinum toxin, which cleaves SNARE proteins and mediates beneficial muscle paralysis in numerous therapeutic and cosmetic applications^2^.

Researchers have engineered or evolved industrial proteases with enhanced thermostability and solvent tolerance^3, 4^. Similarly, a handful of therapeutic proteases have been engineered with improved kinetics and prolonged activity^5–7^. The potential of proteases to serve as a broadly useful platform for modulating the activity of a protein of interest, however, is greatly limited by the native substrate scope of known proteases. In contrast to the highly successful generation of monoclonal antibodies with tailor-made binding specificities^8^, the generation of proteases with novel protein cleavage specificities has proven to be a major challenge. For example, efforts to engineer trypsin variants that exhibit the substrate specificity of the closely related protease chymotrypsin were unsuccessful until researchers grafted the entire substrate-binding pocket, multiple surface loops, and additional residues from chymotrypsin^9–12^. This approach of replacing protease residues with amino acids from related proteases to impart specificity features from the latter^13, 14^ has not provided proteases with specificities not already known among natural proteases, prompting researchers to instead turn to laboratory evolution to generate proteases with novel specificities^15–19^.

Although most reports describe evolution of proteases that cleave single mutant substrates, the evolution of a protease that can degrade a target protein of interest will almost always require changing substrate sequence specificity at more than one position within a substrate motif. Simultaneous specificity changes at two protease sub-sites has previously been accomplished in a stepwise fashion requiring distinct rounds of evolution^20^. Continuous evolution strategies, which require little or no researcher intervention between generations^21^, therefore may be well-suited to evolve over many generations proteases capable of cleaving a target protein that differs substantially in sequence from the preferred substrate of a wild-type protease. In phage-assisted continuous evolution (PACE), a population of evolving selection phage (SP) is continuously diluted in a fixed-volume vessel (the “lagoon”) by an incoming culture of host *Escherichia coli* ^22^. The SP is a modified phage genome in which the evolving gene of interest has replaced gene III, a gene essential for phage infectivity. If the evolving gene of interest possesses the desired activity it will trigger expression of gene III from an accessory plasmid (AP) in the host cell, thus producing infectious progeny encoding active variants of the evolving gene. The mutation rate of the SP is controlled using an arabinose-inducible mutagenesis plasmid (MP) such as MP6, which encodes six mutagenic proteins that disrupt DNA replication proofreading, base excision repair, mismatch repair, and export of damaged nucleobases. Upon full induction, this plasmid increases the mutation rate of the SP by ∼300,000 fold^23^. Because the rate of continuous dilution is slower than phage replication but faster than *E. coli* replication, mutations only accumulate in the SP.

Here we describe the use of PACE to continuously evolve TEV protease, which natively cleaves the consensus substrate sequence ENLYFQS, to cleave a target sequence, HPLVGHM, that differs at six of seven positions from the consensus substrate and is present in an exposed loop of the pro-inflammatory cytokine IL-23. After constructing a pathway of evolutionary stepping stones and performing ~2500 generations of evolution using PACE, a resulting protease contains 20 amino acid substitutions, cleaves human IL-23 at the intended target peptide bond, and blocks the ability of IL-23 to stimulate IL-17 production in a murine splenocyte assay. Together, these results establish a strategy for generating proteases with specificities that have been altered at several substrate positions in order to cleave a target protein of interest.

## Results

### Choice of target substrate and protease

The biochemistry, substrate specificity^24^, and structure^25^ of TEV protease have been extensively studied. Previous directed evolution efforts yielded TEV protease variants with altered specificity at any one of the following positions: P6, P1, or P1ʹ^15, 16, 18, 19^. Based on these studies and our own experiments in which we evolved TEV protease variants that accept substitutions at P6 or P1, we created an “evolvability” scoring matrix (Supplementary Table 1) that assigns a difficulty score to each of the 20 possible amino acids at each of the seven positions recognized by TEV protease. We used this matrix to rank all possible heptapeptides within all human extracellular proteins. From this ranking, we manually curated a handful of disease-associated proteins in which the target peptide sequences were predicted to be solvent exposed based on their crystal structures (Supplementary Table 2).

The resulting candidate target sequences included HPLVGHM, a peptide found in human IL-23. IL-23 is a pro-inflammatory cytokine secreted by macrophages and dendritic cells in response to pathogens and tissue damage, ultimately promoting an innate immune response at the site of injury or infection. This immune response is mediated by IL-23-dependent stabilization of Th17 cells, a class of T helper cells that produce pro-inflammatory cytokines IL-17, IL-6, and TNFα^26^. Hyperactivity of this pathway can lead to a variety of autoimmune disorders including psoriasis and rheumatoid arthritis^27^. Monoclonal antibodies that neutralize IL-23 are FDA-approved drugs for the treatment of psoriasis and show promise in late-stage clinical trials for other autoimmune disorders^28^. These studies suggest that a protease that catalyzes IL-23 degradation may have anti-inflammatory activity.

The target peptide HPLVGHM differs from the TEV consensus substrate sequence, ENLYFQS, at six of seven positions. Two of these substitutions are predicted to not substantially impact TEV protease activity due to its low specificity at positions P5 and P1ʹ, while the other four substitutions occur at positions that are known to be crucial specificity determinants of wild-type TEV protease (P6 Glu, P3 Tyr, P2 Phe, and P1 Gln). Indeed, substitution of TEV substrate P2 Phe or P1 Gln with the corresponding IL-23 substrate residue (P2 Gly or P1 His) has been shown to reduce TEV protease activity by more than an order of magnitude in each case^24^. Encouragingly, researchers previously used site-saturation mutagenesis and an elegant yeast display screen to identify TEV mutants that accept P1 His instead of the P1 Gln, demonstrating the evolvability of P1 recognition^19^.

### Evolution of TEV variants that cleave the IL-23 peptide

**Fig. 1:**
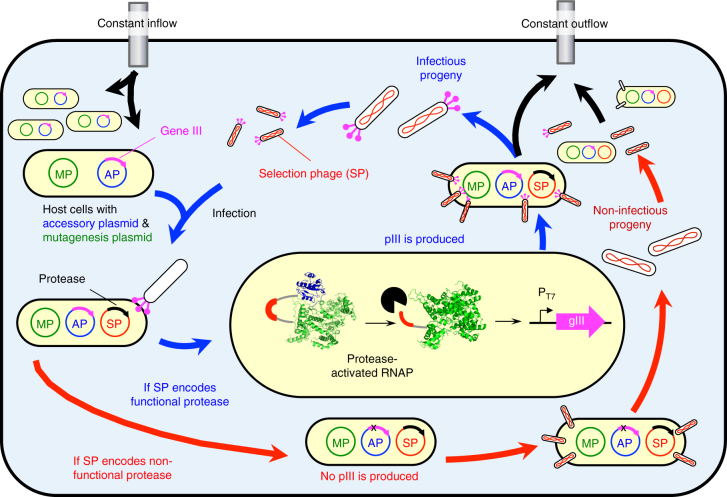
Overview of protease PACE. A culture of host *E. coli* continuously dilutes a fixed-volume vessel containing an evolving population of selection phage (SP) in which essential phage gIII has been replaced by a protease gene. These host cells contain an arabinose-inducible mutagenesis plasmid (MP) and an accessory plasmid (AP) that supplies gIII. The expression of gIII is made protease-dependent through the use of a protease-activated RNA polymerase (PA-RNAP) consisting of T7 RNA polymerase fused through a cleavable substrate linker to T7 lysozyme, a natural inhibitor of T7 RNAP transcription. If an SP encodes a protease capable of cleaving the substrate linker, then the resulting liberation of T7 RNAP leads to the production of pIII and infectious progeny phage encoding active proteases. Conversely, SP encoding proteases that cannot cleave the PA-RNAP yield non-infectious progeny phage

PACE requires linking the activity of interest to expression of an essential phage gene (such as gene III) and thus phage survival. We previously established such a linkage for a range of activities including RNA polymerase activity, DNA binding, protein binding, and protein cleavage^22, 29–33^. To link proteolysis to gene expression we used a protease-activated RNA polymerase (PA-RNAP) consisting of T7 RNA polymerase (T7 RNAP) fused through a protease-cleavable linker to T7 lysozyme, a natural inhibitor of T7 RNAP (Fig. 1)^32^. In this study the PA-RNAP is expressed from the same host-cell AP that places gene III expression under control of the T7 promoter, while the evolving protease is expressed from the SP (Supplementary Figs. 1 and 2).

We verified that SP expressing TEV protease S219V, a form that does not self-cleave (hereafter referred to as “wild-type”), propagate robustly on host cells expressing a PA-RNAP containing the TEV consensus substrate, ENLYFQS, in the linker connecting T7 RNAP and T7 lysozyme. In contrast, replacing the PA-RNAP linker with the target peptide HPLVGHM results in a failure of phage to propagate and rapid clearance of phage, consistent with the inability of wild-type TEV protease, or TEV variants containing a handful of immediately accessible mutations, to cleave the target IL-23 peptide.

**Fig. 2:**
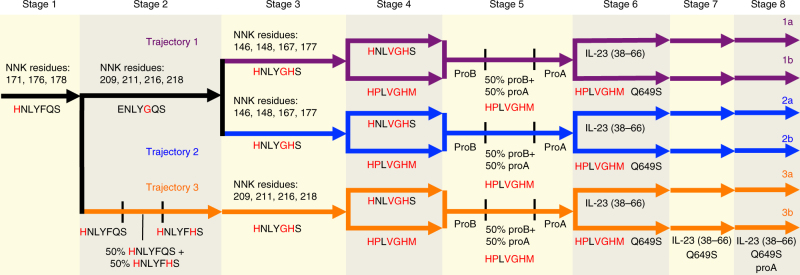
PACE evolutionary trajectories. Across the eight stages of PACE along three diverging trajectories (shown in purple, blue, and orange), each arrow represents a PACE experiment with the corresponding substrate peptide and selection stringency parameters listed beneath the arrow. Increased selection stringency annotations are: Q649S (a T7 RNAP mutant with decreased transcriptional activity), proA (lower expression of substrate PA-RNAP), and IL-23 (38–66) (native IL-23 sequence in place of GGS linker). Numbers above the arrows denote TEV protease residues that were targeted in site-saturation mutagenesis libraries used to initiate that PACE experiment. In the first PACE experiment, wild-type TEV protease was mutagenized at the positions shown. All other libraries were generated using the protease genes emerging from the previous PACE stage as the PCR template. For PACE stages with no targeted mutagenesis, lagoons were inoculated with an aliquot of the phage population from the preceding experiment

We anticipated that successful evolution of TEV protease variants that cleave the IL-23 target would require multiple evolutionary stepping stones^20, 29–31, 33^ to guide evolving gene populations through points in the fitness landscape that bring them successively closer to activity on the final target substrate. We designed three evolutionary trajectories such that substrate changes known to strongly disrupt the activity of wild-type TEV protease, including P6 His, P2 Gly, and P1 His,^24^ were introduced in the earliest stepping stones (Fig. 2). We confronted these challenging substitutions first while the evolving protease populations had access to variants with wild-type-like levels of activity, reasoning that the likelihood of success was higher while proteases had sufficient activity to exchange for altered specificity without falling below a minimum activity threshold needed to survive selection. We introduced these challenging substrate changes one stepping stone at a time to avoid collapse of the evolving phage population and to illuminate at each stage how mutations within TEV protease altered substrate specificity.

We began all three evolutionary trajectories (Fig. 2) by introducing the P6 His substitution (HNLYFQS) into the PA-RNAP and expressing a site-saturation mutagenesis library of TEV protease from the SP. Using NNK codons, we simultaneously randomized three TEV protease residues N171, N176, and Y178, all of which are proximal to the P6 substrate residue. This first PACE yielded variants with enhanced apparent activity on the HNLYFQS substrate (Supplementary Fig. 3) and genotypes were highly enriched for D127A+S135F+N176I, or I138T+N171D+N176T (Supplementary Table 3). Mutations N171D and N176T have been previously characterized as allowing P6 tolerance for uncharged residues such as threonine and proline^15^.

Next we pursued two parallel lines of PACE using either ENLYGQS (trajectories 1 and 2) or HNLYFHS (trajectory 3) as the second stepping-stone substrate. For trajectories 1 and 2, we diversified the population that emerged from PACE on the first stepping stone (HNLYFQS) with NNK codons at all four TEV protease residues (209, 211, 216, and 218) that line the hydrophobic pocket that is occupied by the P2 Phe and performed PACE using host cells expressing ENLYGQS. The resulting population of TEV mutants is typified by the mutations N176I, V209M, W211I, M218F (Supplementary Table 4), which confer apparent cleavage activity on both HNLYFQS and ENLYGQS substrates (Supplementary Fig. 4).

In trajectory 3, we used a mixing strategy to access TEV proteases that could cleave the HNLYFHS stepping-stone double mutant substrate. Unlike PACE experiments initiated from a site-saturation mutagenesis library, a mixing strategy relies on a transitional period of phage propagation on a mixture of two different host cell populations, one expressing an accepted substrate (HNLYFQS) and the other expressing the next stepping-stone substrate (HNLYFHS). Following this transitional period, the SP is propagated exclusively on hosts expressing the next stepping-stone substrate (HNLYFHS). The variants that emerged from this stage of trajectory 3 showed weak apparent activity on the double mutant substrate HNLYFHS (Supplementary Fig. 5), and only a single additional enriched mutation D148A (Supplementary Table 5). Encouragingly, mutation at residue D148 has previously been reported to enable activity on ENLYFHS^19^.

Due to the low apparent activity of proteases emerging from this mixing experiment, we relied on the site-saturation mutagenesis strategy to evolve activity on the third stepping stone, HNLYGHS. The TEV protease populations from trajectory 3 were randomized at sites implicated in P2 recognition (209, 211, 216, and 218), while for trajectories 1 and 2 the TEV protease population was simultaneously randomized at four sites (146, 148, 167, and 177) as previously described for the reprogramming of TEV specificity at the P1 position^19^ (Fig. 2). The primers used to randomize TEV protease residues 167 and 177 must also encode the identity of intervening amino acids N171 and N176. Although the population appeared to converge on N176I (Supplementary Table 4), we reasoned that it was best to preserve genetic diversity at N176 by constructing one library with primers encoding N176I (trajectory 1) and another with N171D+N176T (trajectory 2). Libraries constructed for all three trajectories were then subjected to PACE on host cells expressing the triple mutant substrate HNLYGHS. The variants emerging at this stage of trajectory 1 and 2 were enriched for mutations at residues 146, 148, and 177, consistent with acceptance of the newly introduced P1 substitution^19^. Similarly, clones from trajectory 3 exhibit mutations at residues 209, 211, and 218 that may promote acceptance of the newly added P2 Gly substitution. Regardless of trajectory, all clones emerging at this stage exhibit at least one mutation from each of three targeted mutagenesis libraries (Supplementary Table 6), suggesting that they have evolved activity on the triple mutant substrate.

Given the known tolerance of TEV protease for amino acids at positions P5 and P1ʹ, we speculated that proteases evolved to recognize the triple mutant substrate HNLYGHS might already exhibit activity on the final target substrate (HPLVGHM). Indeed, the populations arising from evolution on the triple mutant substrate successfully propagate in PACE on host cells producing the HPLVGHM substrate, and the resulting variants display weak apparent activity on the final target substrate (Supplementary Fig. 6). In order to evolve high levels of activity on the final target substrate from these weakly active mutants, we applied three strategies to increase selection stringency on all three trajectories: (1) express a lower concentration of the PA-RNAP substrate by using a weaker constitutive promoter (proA instead of proB)^34^; (2) replace the flexible GGS linker that flanks our substrate with the native and potentially structured amino sequence from IL-23 (human IL-23 residues 38–66); and (3) introduce a mutation in the T7 RNAP portion of the PA-RNAP that decreases transcriptional activity (Q649S)^35^. We confirmed that all three strategies indeed increased selection stringency (Supplementary Fig. 7).

We first applied the lowered substrate concentration strategy using a mixing experiment to transition from proB to proA expression of the PA-RNAP; this experiment yielded modest changes in genotypes. Exploiting the ease of performing PACE on multiple lagoons in parallel, we implemented the other two strategies simultaneously on all three trajectories. The resulting six populations (trajectories 1a, 1b, 2a, 2b, 3a, and 3b; see Fig. 2) were carried forward into PACE on hosts expressing a PA-RNAP with both the IL-23 (38–66) linker and the attenuated T7 RNAP mutant Q649S. In the final stage of PACE for all six populations, we used a proA promoter to generate less of the PA-RNAP containing the IL-23 (38–66) linker and the Q649S mutation. This series of stringency modulation experiments produced variants with higher levels of apparent activity on the final HPLVGHM substrate (Supplementary Fig. 8).

### Characterization of evolved TEV protease variants

Mutations that arise early in long evolutionary trajectories can create a cascade of contingencies because subsequent mutations must be compatible with the preexisting genetic context, a phenomenon known as epistasis. Genotypes suggest that epistasis strongly shaped the outcomes of trajectories 1 and 2, which were dominated by N176I vs. N171D+N176T, respectively, prior to the third stage of PACE. During subsequent evolution, the amino acid identity at amino acid 176 appears to have dictated the optimal identity of residue 177, such that the combinations N176I+N177S, or N176T+N177M, predominate trajectories 1 and 2 respectively. Swapping the identity of N177 between clones from trajectories 1 and 2 results in a substantial loss of activity (Supplementary Fig. 9), further consistent with epistasis at this position. It is likely that these genetic differences between trajectory 1 and 2 also later led to the enrichment of distinct mutations outside of the substrate-binding site (Supplementary Tables 3–11). For example, mutations at position 203 persisted only in trajectory 1, while mutations at positions 28, 30, 68, 132, and 162 were only abundant in trajectory 2.

Unsurprisingly, we observed a dramatically different outcome in the third trajectory, which not only experienced a different schedule of stepping-stone substrates but was also subjected to a mixing experiment instead of NNK mutagenesis at residues 146, 148, 167, and 177. Our data are consistent with a model in which a lack of diversification at these critical residues traps trajectory 3 in a local fitness maximum, evidenced by weak apparent activity on the final target substrate (Supplementary Figs. 6 and 8) and few genotypic changes after the fifth stage of trajectory 3 (Table 1). Consequently, while all six populations yielded TEV variants with apparent activity on the final target, the TEV protease variants that exhibited the highest apparent activity on the final target were all derived from trajectories 1 and 2.

**Table 1:** Representative evolved TEV protease genotypes

*Trajectory 1*																													
1								D127A		S135F								N176I											
2								D127A		S135F								N176I			V209M	W211I			M218F				
3								D127A		S135F		T146A	D148P					N176I	N177R		V209M	W211I			M218F				
4								D127A		S135F		T146A	D148P					N176I	N177R		V209M	W211I			M218F				K229E
5	E2K							D127A		S135F		T146C	D148P			S170A		N176I	N177S	R203Q	V209M	W211I			M218F	Q226stop			
6	E2K							D127A		S135F		T146C	D148P			S170A		N176I	N177S	R203Q	V209M	W211I			M218F	Q226stop			
7	E2K							D127A		S135F		T146C	D148P			S170A		N176I	N177S	R203Q	V209M	W211I			M218F	Q226stop			
8	E2K							D127A		S135F		T146C	D148P			S170A		N176I	N177S	R203Q	V209M	W211I			M218F	Q226stop			
*Trajectory 2*																													
1											I138T						N171D	N176T											
2								D127A		S135F							N171D	N176T			V209M	W211I			M218F				
3								D127A		S135F		T146S	D148P				N171D	N176T	N177M		V209M	W211I			M218F				K229E
4								D127A		S135F		T146S	D148P		F162S		N171D	N176T	N177M		V209M	W211I			M218F				K229E
5								D127A		S135F		T146S	D148P		F162S		N171D	N176T	N177M		V209M	W211I			M218F				K229E
6					R50G		E107D	D127A		S135F		T146S	D148P		F162S	S170A	N171D	N176T	N177M		V209M	W211I			M218F				K229E
7				T30A				D127A		S135F		T146S	D148P		F162A	S170A	N171D	N176T	N177M		V209M	W211V	K215E		M218F				K229E
8		T17S	H28L	T30A		N68D	E107D	D127A	F132L	S135F		T146C	D148P	S153N	F162S	S170A	N171D	N176T	N177M		V209M	W211I			M218F				K229E
*Trajectory 3*																													
1								D127A		S135F								N176I											
2								D127A		S135F			D148A					N176I											
3							E107D	D127A		S135F		T146A	D148A					N176I			V209E	W211L		V216I	M218W				
4							E107D	D127A		S135F		T146A	D148A					N176I			V209F	W211C			M218L				
5			H28Y				E107D	D127A		S135F		T146A	D148A	S153N				N176I			V209F	W211C			M218L	Q226S	P227A	V228S	K229stop
6			H28Y				E107D	D127A		S135F		T146A	D148A	S153N				N176I			V209F	W211C			M218L	Q226S	P227A	V228S	K229stop
7			H28Y	T30A			E107D	D127A		S135F		T146A	D148A	S153N				N176I			V209F	W211C	K215E		M218L	Q226P	P227A	V228S	K229stop
8			H28Y				E107D	D127A		S135F		T146A	D148A	S153N				N176I			V209F	W211C	K215E		M218L	Q226P	P227A	V228S	K229stop

For each stage of each PACE trajectory, a representative evolved protease clone emerging at the end of the stage (numbered in the first column) is shown. The mutations within each clone are listed as an individual row, illustrating the enrichment of new mutations during successive stages of PACE as well as genotypic differences between trajectories. The enriched stop codon mutations truncate 8 or 11 amino acids of the C-terminus of the protein and preserve proteolytic activity

Three representative proteases from the end of trajectories 1 and 2 were purified and assayed in vitro for their ability to cleave a model protein substrate in which MBP and GST were fused through a linker containing the final HPLVGHM substrate sequence (Supplementary Fig. 10). All three evolved proteases cleaved the model substrate. We selected the most active clone (TEV L2F from trajectory 2, containing 20 non-silent mutations, Supplementary Table 11) for detailed characterization. We assayed the kinetic parameters of this mutant enzyme on wild-type (ENLYFQS) and target (HPLVGHM) substrate peptides using a previously described HPLC method^19^. Unlike the wild-type enzyme, which exhibits no detectable activity on the HPLVGHM peptide, the L2F variant processes this substrate with ∼15% of the catalytic efficiency (*k*_cat_/*K*_M_) with which TEV protease cleaves its native substrate (Table 2 and Supplementary Fig. 11). Compared to wild-type TEV, evolved TEV L2F appears to have slightly improved kinetics on the canonical ENLYFQS substrate, possibly due to broadly activating mutations that increase *k*_cat_, while experiencing only a modest fivefold increased *K*_m_ on the target substrate HPLVGHM. These results collectively indicate that PACE generated a mutant protease that cleaves a target substrate containing mutations at six positions with only modestly lower efficiency than wild-type TEV protease cleaves its consensus substrate.

**Table 2:** Kinetic parameters of wild-type and evolved TEV proteases

Substrate	Protease	*k*_cat_ (s^−1^)	*K*_m_ (μM)	*k*_cat_/*K*_m_ (s μM)^−1^
ENLYFQS	Wild-type	0.23 ± 0.011	170 ± 22	1.3 × 10^−3^
HPLVGHM	Wild-type	ND	ND	ND
ENLYFQS	TEV L2F	0.27 ± 0.019	150 ± 28	1.7 × 10^−3^
HPLVGHM	TEV L2F	0.19 ± 0.010	920 ± 110	2.0 × 10^-4^

ND = not detected (no product formation observed after 30 min of incubation of 1 μM protease and 2 mM substrate with a limit of detection of 1 nM product)

### Substrate specificity profiling of an evolved TEV protease

**Fig. 3:**
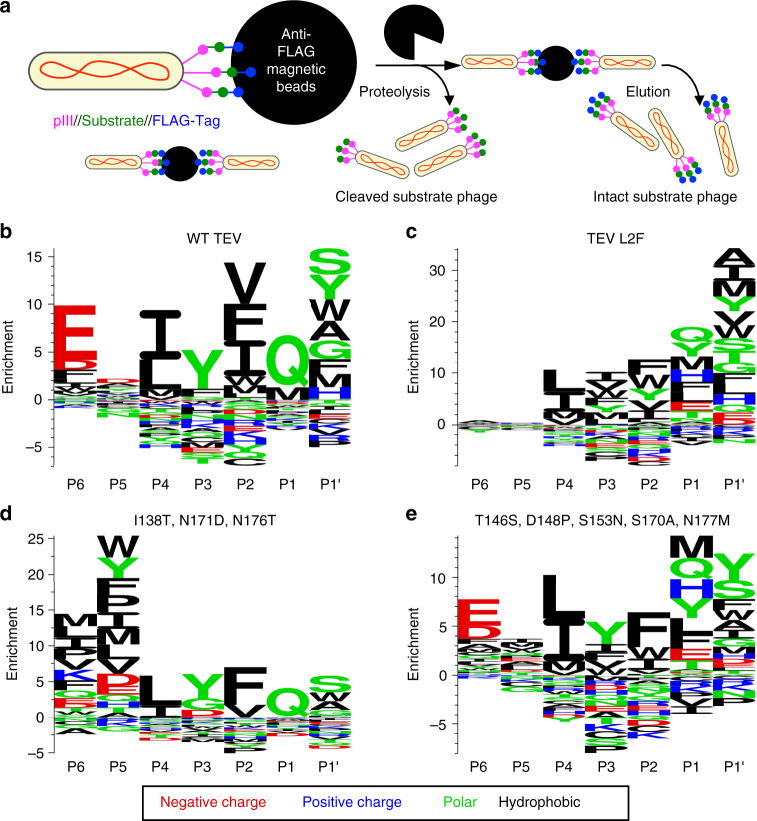
Protease specificity profiling. **a** Overview of phage substrate display. M13 bacteriophage libraries encode pIII fused to a FLAG-tag through a randomized protease substrate linker. These substrate phage are bound to anti-FLAG magnetic beads and treated with a protease to release phage that encode substrates that can be cleaved by the protease. The remaining intact substrate phage are eluted with excess FLAG peptide. The abundance of all substrate sequences within the cleaved and eluted samples is measured by high-throughput sequencing. **b**–**e** For all assayed proteases, phage substrate display was separately performed on seven libraries, each with a different single randomized position within the ENLYFQS motif. The results are displayed as sequence logos, with letter height proportional to enrichment in the cleaved versus eluted sample. Letters placed above the *x*-axis indicate protease acceptance and letters beneath the axis indicate rejection. **b** Wild-type TEV protease exhibits strong enrichment for the consensus motif EXLYFQS. **c** Evolved TEV L2F has broadened specificity at P6 and shifted specificity at P3, P1, and P1ʹ in accordance with the HPLVGHM target substrate. **d** Mutations I138T, N171D, and N176T are sufficient to broaden P6 specificity. **e** Mutations T146S, D148P, S153N, S170A, and N177M shift specificity at both P1 and P3

Proteolysis assays on individual substrates reveal that evolved TEV protease L2F maintains the ability to detectably cleave starting and intermediate substrates while acquiring activity on the final IL-23 target (Supplementary Fig. 12). A more comprehensive understanding of the substrate specificity of this evolved enzyme requires an unbiased protease specificity profile generated from a large number of substrate variants. To obtain such a profile, we applied a previously reported phage substrate display method (Fig. 3a)^36–38^. M13 bacteriophage encoding pIII fused to a FLAG-tag through a library of substrate linkers were immobilized on anti-FLAG magnetic beads. When incubated with a protease of interest, phage encoding cleaved substrates are liberated from the solid support, while phage encoding the intact substrates remain immobilized and are eluted with excess FLAG peptide. The abundance of each substrate in the cleaved and eluted populations was measured by high-throughput DNA sequencing, yielding enrichment values (Supplementary Table 12) and sequence logos (Fig. 3b–e) that convey protease substrate specificity across all possible amino acids^39^.

We applied this specificity profiling technique to seven separate libraries, each containing a single randomized position within the canonical ENLYFQS substrate. These libraries are inherently biased by the identities of the residues that are held constant, but because of their small theoretical diversity, they are easy to construct and a single round of selection yielded robust enrichment values. We validated this method by enrichment of the consensus motif EXLYFQS (where X = any amino acid) for wild-type TEV protease (Fig. 3b). We also applied this substrate specificity profiling method to more complex libraries containing sets of three consecutive randomized amino acids within either the ENLYFQS or HPLVGHM substrate. The resulting specificity profiles from these larger libraries (Supplementary Fig. 13) did not substantially differ from the results of the single-site libraries. However, the identity of the constant residues (ENLYFQS vs. HPLVGHM) did alter the resulting specificity profiles of TEV L2F; the specificity of TEV L2F is more pronounced for P1 His, the target residue, and P6 Glu, the wild-type residue, in the context of HPLVGHM libraries (Supplementary Fig. 13 and corresponding enrichment values in Supplementary Table 13).

When we compare the specificity profile of evolved TEV L2F (Fig. 3c) to that of wild-type TEV, a number of differences are apparent: TEV L2F shows a shifting of P3 specificity towards aliphatic residues Ile and Val, a shifting of P1 specificity towards His, a shifting of P1’ specificity towards aliphatic amino acids Ala, Ile, and Met, and a broadening of specificity at P6. These changes are consistent with evolutionary pressure to cleave the target substrate HPLVGHM. Although the evolved L2F protease recognizes a shortened motif due to loss of P6 specificity, it retains the ability to reject the substantial majority of amino acids at each of the five others positions used by TEV. The overall specificity of the evolved L2F protease lies well within the range of specificities exhibited by natural proteases such as caspases, granzymes, clotting factors, and MMPs, which typically specify strongly only one or two positions and accept mixtures of several to many amino acids at other positions, yet retain sufficient overall specificity to mediate physiological signaling roles^40–43^. Nonetheless, the use of evolved proteases in complex biological environments may require a proteome-wide evaluation of off-target substrates to identify potential undesired effects.

### Functionally independent groups of TEV mutations

To illuminate the molecular basis of the evolved changes in substrate specificity, we generated TEV mutants containing small subsets of mutations and profiled their substrate specificities using substrate libraries in which a single residue of the ENLYFQS substrate was randomized. A number of mutations were predicted to influence solubility and stability based on previous reports^44–46^ or their distance from the substrate in the crystal structure^25^. We constructed various combinations of the predicted solubility mutations (T17S, N68D, E107D, D127A, F132L, S135F, F162S, R203Q, K215E, and K229E) as well as mutants that putatively influence specificity at P1 (T146S, D148P, S153N, S170A, and N177M), P6 (I138T, N171D, and N176T), and P2 (V209M, W211I, and M218F) based on the emergence of these mutations during PACE.

All of the tested combinations of mutations resulted in proteases that retained activity to varying degrees (Supplementary Fig. 14), despite being taken out of their PACE-evolved contexts. As expected, the solubility-enhancing mutants exhibited no significant change in specificity (Supplementary Fig. 15). The P2 variant also did not display any substantial specificity changes, consistent with the lack of a strong change in P2 specificity in the TEV L2F specificity profile (Supplementary Fig. 15).

Mutations in the P6 variant (I138T, N171D, N176T) are sufficient to confer loss of glutamate specificity at P6 with no other obvious changes to substrate preferences (Fig. 3d), suggesting some degree of modularity in protease–substrate interactions. Conversely, the P1 variant (T146S, D148P, S153N, S170A, and N177M) not only exhibits broadened specificity at the P1 site, but also shows a concurrent increased affinity for P3 aliphatic side chains (Fig. 3e). The mutations within these two variants appear to be responsible for the three largest differences in substrate specificity between wild-type TEV and TEV L2F.

### Evolved TEV L2F cleaves human IL-23

Next we tested the ability of the evolved TEV L2F protease to cleave full-length human IL-23 protein. In its active form, IL-23 is a heterodimer between the IL-12p40 subunit and the IL-23p19 subunit. We incubated TEV L2F with IL-23 in either its heterodimeric or monomeric p19 state, and observed by Western blot the formation of a single cleavage product for the IL-23 heterodimer, and in the presence of excess protease, two cleavage products for the monomeric IL-23p19 substrate (Supplementary Fig. 16).

IL-23 digestion reactions were subjected to LC–MS to identify the cleavage products. The heterodimer cleavage reaction generated a single protein product of mass 3598 Da less than the starting material, matching the fragment liberated by cleavage of the target peptide bond at the HPLVGH//M sequence (Supplementary Fig. 17). Data from the monomer cleavage reaction in the presence of a 1.5-fold excess of TEV L2F revealed two new product masses corresponding to a single cleavage at the target site (HPLVGH//M), as well as proteolysis at both the target site (HPLVGH//M) and an additional secondary site (ARVFAH//G) that is also consistent with the L2F specificity profile shown in Fig. 3c (Supplementary Fig. 18). The absence of an ion corresponding to IL-23 cleaved at only the secondary site suggests that the target site is kinetically favored by TEV L2F. The secondary site was only cleaved in the monomeric substrate and not the heterodimer presumably because it is occluded by the IL-12p40 subunit in the heterodimer structure (Supplementary Fig. 19)^47^.

### Evolved TEV deactivates IL-23 and prevents IL-17 secretion

**Fig. 4:**
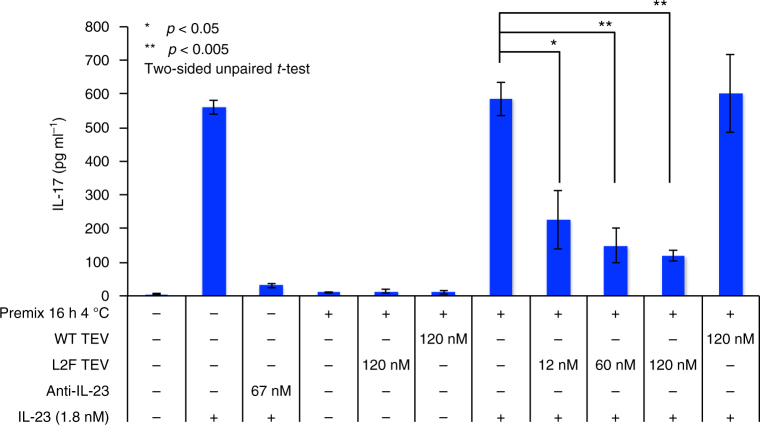
Protease-mediated attenuation of IL-17 secretion in mouse splenocytes. The activity of IL-23 in vivo is mediated by stabilization of a T-helper cell lineage (Th_17_) that secretes IL-17, leading to downstream pro-inflammatory signals. This pathway can be assayed within a culture of mouse mononuclear splenocytes, by measuring the amount of IL-17 secretion into the cell culture media using an ELISA. As a positive control, anti-IL-23 antibodies in a super-stoichiometric ratio prevent IL-17 secretion. Preincubation of IL-23 and evolved TEV L2F attenuates IL-17 secretion, demonstrating that cleavage of the HPLVGHM target site inactivates immune signaling capabilities of IL-23. Values represent the mean and error bars represent the standard deviation of three technical replicates

Finally we tested the ability of the evolved TEV L2F protease to abrogate the biological activity of IL-23. We used a previously described IL-23 activity assay with primary isolates of mouse mononuclear splenocytes^48^. When cultured in the presence of IL-2 and IL-23, Th17 cells are stabilized and secrete IL-17 into the media supernatant, which is quantified by ELISA. We observed a dose-dependent attenuation of IL-17 production when IL-23 was pre-incubated with TEV L2F (Fig. 4). These samples were also visualized by western blot demonstrating that the p40 subunit is unaffected by incubation with protease, and that inhibition of IL-17 production is causally linked to IL-23p19 cleavage (Supplementary Fig. 20). A sub-stoichiometric dose of L2F protease (0.40 equivalents) resulted in the loss of nearly all IL-23-induced IL-17 secretion, consistent with TEV L2F catalyzing cleavage of IL-23 following multiple turnover Michaelis–Menten kinetic behavior that we observed in previous experiments using synthetic peptide substrates (Table 2 and Supplementary Figs. 21 and 22). Direct addition of TEV L2F to splenocyte cultures in serum-containing media supplemented with IL-23 did not attenuate IL-17 secretion (Supplementary Fig. 23). The presence of an equivalent concentration of serum did not inhibit cleavage in vitro (Supplementary Fig. 24), suggesting that TEV protease activity could be diminished in the oxidizing cell culture supernatant. Alternatively, it is possible the protease is sequestered by other secreted factors or cell-surface proteins within the complex culture media, or that IL-23 binding to IL-23R may occur faster than IL-23 proteolysis.

## Discussion

The generation of on-demand biochemical catalysts has been a longstanding interest of the scientific community^49^. Previous efforts to evolve protease specificity have been successful at altering the substrate specificity of model proteases by one or two amino acids^15–20^. By using PACE to conduct ∼2500 total generations of evolution in three diverging evolutionary trajectories, evolutionary stepping stones to guide populations through long evolutionary trajectories, and both targeted and elevated random mutagenesis, we evolved TEV protease variants that cleave a substrate dramatically different from the wild-type substrate with only a modest decrease in kinetic parameters compared with wild-type TEV on its consensus substrate. This work demonstrates for the first time that a protease can be reprogrammed through laboratory evolution to cleave and deactivate a target protein containing a very different substrate sequence than is recognized by the wild-type protease. Our approach also exemplifies a target selection strategy that integrates knowledge from known positional tolerances of a wild-type enzyme with results from previous evolution efforts. The above data also demonstrate that evolved L2F protease has sufficient specificity to avoid degrading itself, the numerous proteins required for the PACE selection, MBP, GST, or essential protein components of the IL-23 assay beyond IL-23. The ability of the L2F protease to cleave IL-23 is also not inhibited by the presence of 10% fetal bovine serum, which contains high concentrations of other proteins. The specificity of the evolved L2F protease overall resembles that of many natural proteases, which like L2F reject the majority of possible amino acids but accept mixtures of others at each recognized substrate position^40–43^.

While these advances suggest the utility of evolved proteases for many research and industrial applications, they face additional challenges for therapeutic applications. The evolution of proteases under many generations of positive selection alone can result in proteases that accept the target substrate but do not reject the wild-type or intermediate substrates. In cases in which it is desirable to reject cleavage of certain non-target substrates, the application of a PACE negative selection strategy to apply selection pressure against non-target cleavage may be useful^30^. Moreover, any circulating foreign protein therapeutic if administered repeatedly poses a substantial immunogenicity risk. Consequently, ideal starting points for therapeutic protease evolution may be circulating human proteases, such as the use of human kallikrein 7 in a pioneering study that sculpts its specificity to more selectively proteolyze human amyloid beta^50^. The PACE of a human protease will present a significant but tractable technical hurdle, producing enzymes containing disulfide bonds in active forms in the *E. coli* cytosol^51–54^.

These challenges notwithstanding, this study represents a foundation for the directed evolution of proteases with highly altered specificities. We demonstrate the feasibility of catalytic inactivation of a target protein with an evolved protease, which in some cases may offer substantial benefits over stoichiometric binding of a neutralizing antibody. In addition to potency advantages, we anticipate that evolved proteases may also enable research and therapeutic applications that are unavailable to antibodies such as proteolysis-induced gain-of-function and proteolysis-mediated alteration of a protein’s import, export, subcellular localization, half-life, or post-translational modification state.

## Methods

### Ranking of target sites within extracellular proteins

A list of human extracellular and transmembrane proteins with their corresponding amino acid sequences were tabulated using the ProteinData functionality in Mathematica 10. This data was transferred into MATLAB for further processing by a customizable script that performed the following operations (Supplementary Note 1). A rating matrix that is seven positions wide (for the seven sites within the TEV protease recognition motif) by 20 long (for each possible amino acid) was manually populated with subjective “evolvability” integer ratings. Each protein was converted into a binary sparse matrix with as many rows as the length of the protein sequence and 20 columns one for each amino acid. For each protein matrix, seven rows at a time were multiplied by the “evolvability” matrix, with the trace of the resulting 7 × 7 product matrix providing a score for the heptapeptide. For each extracellular protein the best score and the corresponding peptide and starting-residue index were saved. Once all protein sequences had been processed, we sorted the protein names along with their best-match candidate substrate sequences by score.

### Expression vectors and phage libraries

All primers were designed to perform USER cloning^55^ and ordered from Integrated DNA Technologies (IDT). For the cloning of phage libraries, NNK codons were generating using hand-mixed phosphoramidite ratios to provide uniform incorporation rates (Supplementary Table 15). All PCR reactions were performed using Phusion U Hot Start polymerase (Thermo-Fisher).

For the assembly of APs and expression vectors, PCR products were purified using EconoSpin columns (Epoch Life Sciences) and assembled with DpnI and USER enzyme in CutSmart Buffer (New England BioLabs). Following assembly, plasmids were transformed into NEB Turbo Competent *E. coli* cells (New England BioLabs).

For the assembly of phage libraries, PCR products were purified by gel electrophoresis and extracted using the MinElute kit by Qiagen. Following an assembly reaction identical to that of the AP, the USER reaction was desalted using the MinElute PCR purification kit (Qiagen) prior to electroporation into competent *E. coli* S1059^22^ (for SP libraries) or NEB Turbo Electrocompetent *E. coli* cells^22^ (for substrate display phage libraries). Phage libraries were grown overnight in 2×YT and filtered through sterile 0.22 μm membranes to eliminate host cells. The titers of phage libraries were evaluated by plaque assay using strain S1059 as hosts. Briefly, phage were prepared in four 50-fold serial dilutions of 50 µl. To each dilution was added 100 µl of fresh host cell culture at approximately OD_600_ = 1.0 followed by addition of 900 µl top agar (2×YT, 6 g L^−1^ agar). The mixture was mixed by pipet, then transferred to a quarter-plate prepared with a thin layer of bottom agar (2×YT, 16 g L^−1^ agar). Plaque assays were incubated overnight at 37 °C.

In order to assess library quality, 12 clones were sequenced to confirm diversity at the targeted amino acid positions. Briefly, individual plaques were picked with a pipet tip in order to provide template material (SP-infected *E. coli*) for rolling circle amplification (TempliPhi, GE Healthcare). Sanger DNA sequencing was performed using primer BCD1136 (Supplementary Table 15), and results were aligned and tabulated using SeqMan (DNAStar).

### PACE experiments

PACE experiments were performed as follows: ^22, 29–33^. Briefly, *E. coli* strain S1030 was co-transformed by electroporation with a mutagenesis plasmid (MP6)^23^ and an AP (plasmids are described in Supplementary Table 14 and detailed in Supplementary Figs. 1 and 2). Chemostats containing 80 ml of Davis Rich Media with 22.5 μg ml^−1^ carbenicillin and 15 μg ml^−1^ chloramphenicol were inoculated with overnight starter cultures and grown at 37 °C while mixing at 250 rpm via a magnetic stir bar. Once the chemostat grew to ∼OD_600_ = 1.0, we began dilution with fresh media at a rate of 80–100 ml h^−1^, with the waste needle set at a height of 80 ml. At the same time, we began the flow of chemostat culture at ∼10–20 ml h^−1^ into a lagoon with a waste needle set at a height of 15 ml. The total flow rate through each lagoon was set based upon the difficulty of a given experiment, with slower dilution being used for more challenging evolutions. For the full duration of the experiment 10% w/v arabinose solution was syringe-pumped into the lagoons at a rate of 0.5–1.0 ml h^−1^.

Experiments starting with an NNK mutagenized SP library initiated with a lagoon inoculum of 1–2 ml of phage library containing 10^8^–10^10^ pfu ml^−1^. For all other experiments, lagoons were inoculated with 50–100 μl of filtered phage population from the last time point of the previous PACE experiment. In PACE experiments using mixtures of host cell cultures (see the main text), lagoons received an influx of cell culture from two separate chemostats containing hosts bearing two different APs (combined rate of 10–20 ml h^−1^) for a period of 24–48 h.

Phage samples were collected from lagoon waste outflow lines at 24 h intervals and passed through a 0.22 µm sterile filter to remove host cells. The titers of phage samples were evaluated by plaque assay using strain S1059 as hosts. At the end of each PACE experiment, eight individual plaques were picked with a pipet tip in order to provide template material (SP-infected *E. coli*) for rolling circle amplification (TempliPhi, GE Healthcare). The same pipet tip was subsequently transferred to a 96-deep well culture plate containing 2×YT media for growth overnight at 37 °C. After a PACE experiment, enriched mutations should be present within multiple clones of this small sample of eight population members. Sanger DNA sequencing was performed using primer BCD1136 (Supplementary Table 15), and results were aligned and tabulated using SeqMan (DNAStar).

### Luminescence assays of evolved clones

Clones chosen for characterization were sterile-filtered from the corresponding position within the 96-well culture plate. Saturated overnight cultures of S1030 cells containing a substrate AP were used to initiate luciferase assays in 96-well culture plates. Approximate volumes were 500 µl 2×YT, 50 µl overnight starter culture, and 10 µl filtered phage samples. All assays included a negative control (no phage), a positive control (SP encoding T7 RNAP), and wild-type TEV SP as a reference. Experimental and control conditions were performed in triplicate. After 3–5 h of growth in a 37 °C shaker, 100 µl was transferred to a clear-bottom assay plate to measure OD_600_ and luminescence on a Tecan Infinite Pro Plate Reader. Measurements were analyzed as OD-normalized values and as luminescence fold-change over the negative control.

### Purification of TEV proteases and fusion protein substrates

TEV protease was purified as previously described^19, 44–46^, but with minor modifications. Briefly, OneShot BL21 Star (DE3) chemically competent cells (Invitrogen) were transformed with expression vectors encoding MBP fused through a TEV cleavage site to a 6×His-tagged TEV protease. A total of 5 ml of saturated overnight starter culture was added to 1–2 l of LB+kanamycin (40 µg ml^−1^), and grown at 37 °C until OD_600_ = ~0.7. Expression was induced with 1 mM IPTG for 4 h at 30 °C, and cells were harvested by centrifugation at 6000 × *g* for 5 min. The pellet was resuspended in 15–25 ml binding buffer (10% glycerol, 50 mM Tris pH 8.0, 1.0 M NaCl, 1 mM DTT, and 20 mM imidazole) with a Roche Complete EDTA-free protease inhibitor tablet (note that TEV protease is unaffected by conventional protease inhibitors). Cells were lysed by sonication for 4 min with a 1-s on, 1-s off cycle at medium power. Lysate was clarified by centrifugation at 18,000 × *g* for 20 min. Clarified lysate was incubated with 1–2 ml TALON metal affinity resin (Clontech) for 1 h mixing end-over-end at 4 °C. Resin was pelleted at 700 × *g* for 5 min, and resuspended with 10 ml of binding buffer to load onto a gravity flow column. Resin was washed with 10 column volumes of binding buffer, followed by 2 column volumes of bind buffer with imidazole supplemented to 50 mM. TEV protease was eluted with 4 column volumes of elution buffer (10% glycerol, 50 mM Tris pH 8.0, 0.1 M NaCl, 1 mM DTT, and 250 mM imidazole). The purity of fractions was assessed by SDS-PAGE using precast Bolt 4–12% Bis–Tris gels (ThermoFisher), and TEV containing fractions were pooled and concentrated to <250 µl using an Amicon Ultra Centrifugal filter with a 10 kDa molecular weight cut-off (EMD-Millipore). The concentrated sample was further purified to >95% using a SuperDex 200 Increase 10/300 column (GE Healthcare) running with storage buffer (20% glycerol, 50 mM Tris pH 8.0, 0.1 M NaCl, 1 mM DTT). Proteases used in mammalian cell culture were further subjected to endotoxin removal resin (Pierce), followed by assaying with an LAL endotoxin quantification kit (Pierce). Protein concentrations were determined by Bradford Assay (ThermoFisher) and aliquots were frozen in liquid nitrogen for storage at −80 °C.

MBP–GST test substrates were expressed, purified, and stored exactly as described above for TEV, except for the following changes. Expression was induced with 1 mM IPTG for 16 h at 20 °C, and binding buffer was 50 mM Tris pH 8.0, 0.5 M NaCl, supplemented with a Roche Complete EDTA-free protease inhibitor tablet. After sonication and centrifugation, clarified lysate was incubated with 1 ml glutathione-linked sepharose (Clontech) for 1 h mixing end-over-end at 4 °C. Loaded resin was washed with 40 column volumes of binding buffer, followed by 4 column volumes of elution buffer (50 mM Tris pH 8.0, 100 mM NaCl, 10 mM glutathione). Samples were >95% pure as assessed by SDS-PAGE and were dialyzed against storage buffer (20% glycerol, 50 mM Tris pH 8.0, 0.1 M NaCl, 1 mM DTT) using Slide-A-Lyzer Cassettes with a 10-kDa molecular weight cut-off (ThermoFisher).

### Assaying proteolysis of fusion protein substrates

Protease assays consisted of 5 µg of MBP–GST substrate and 1 µg of wild-type or evolved TEV protease incubated for 3 h at 30 °C in storage buffer (20% glycerol, 50 mM Tris pH 8.0, 0.1 M NaCl, 1 mM DTT) supplemented with freshly prepared DTT to a final concentration of 2 mM. Reactions were analyzed by SDS-PAGE and visualized with Coomassie stain.

### HPLC kinetics assay

Protease kinetics were determined as previously described^19^, but with minor adjustments. Briefly, synthetic peptide substrates (THPLVGHMGTRRW-dinitrophenol-lysine and TENLYFQSGTRRW-dinitrophenol-lysine) and synthetic standards for cleaved products (MGTRRW-dinitrophenol-lysine and SGTRRW-dinitrophenol-lysine) were ordered from Genscript. Dinitrophenol moieities provided strong absorbance at 355 nm for more accurate quantification. Reactions and standards were analyzed by HPLC on a C18 reverse-phase column (Kinetex 5μ C18 100A, Phenomenex) using an acetonitrile gradient from 5 to 50%. Standard curves were constructed for both products (MGTRRW-dinitrophenol-lysine and SGTRRW-dinitrophenol-lysine) to enable quantification of reaction progress.

Reactions were carried out with 0.05–0.1 μM protease and 50 μM to 2 mM substrate. Proteases (in storage buffer plus 1 mM freshly prepared DTT) and substrates (in sterile water) were prepared as solutions at 2× concentration (50 µl each) then combined to yield a total reaction volume of 100 µl. Reactions were incubated at 30 °C for 10 min and quenched with 25 µl of 5% TFA. After quenching, protease was eliminated from samples using an Amicon Ultra Centrifugal filter with a 10-kDa cut-off (EMD-Millipore). Prior to conducting reactions in triplicate, all conditions were tested and monitored at 5, 10, 30 min to ensure that 10 min was within the linear range of the reaction (<25% substrate consumption). Peak integrations were tabulated, converted into product concentrations using the standard curves, and fit to the Michaelis–Menten kinetics model using Prism GraphPad.

### Protease specificity profiling

For each combination of library and protease, 60 µl of a 50% suspension of Anti-FLAG M2 Magnetic beads (Sigma) was transferred into a 1.5-ml eppendorf tube. For all subsequent manipulations, a magnetic plate was used to separate beads and allow aspiration of the supernatant. After washing with 1 ml of TBS (20 mM Tris pH 7.0, 150 mM NaCl), beads were incubated with 30–100 µl of substrate phage libraries (titers ranged from 10^8^ to 10^10^ pfu ml^−1^) in 1 ml of TBS at room temperature for 2 h rotating end-over-end. After initial binding, the supernatant was discarded and beads were washed with 1 ml of TBS. Beads with bound substrate phage were incubated in 0.5 ml TBS containing 0.5 µM protease for 2 h. Supernatant containing cleaved substrate phage was recovered, and the beads were again washed with 1 ml of TBS. The remaining bound uncleaved substrate phage was eluted in 0.1 ml of TBS containing 0.1 mg ml^−1^ FLAG peptide (Sigma).

For substrate libraries containing a single randomized amino acid position, a single round of selection was sufficient. For substrate libraries containing windows of three randomized amino acids, a second round of selection was necessary to detect enrichment. In these cases, the round 1 cleaved substrate phage were expanded in overnight cultures consisting of 100 µl cleaved substrate phage and 900 µl S1030 culture (diluted to OD_600_ = ~0.1). Following outgrowth, cultures were centrifuged to pellet *E. coli* prior to aspirating the supernatant phage to be used in a second round of phage substrate display as previously described. Due to expansion biases during outgrowth, these specificity profiles were only interpreted after normalizing to the second round elution of the no-protease control experiments.

### High-throughput sequencing and data analysis

Samples were amplified by PCR using Q5 Hot Start 2× Master Mix (NEB) with 1 µl of template phage sample and primers (MSP819 or MSP820, and MSP824, see Supplementary Table 15). Illumina barcodes were added in a second PCR reaction using 1 µl of the first round PCR material as template. Samples were pooled and purified by gel electrophoresis using a MinElute Gel Extraction kit (Qiagen). The concentration of the pooled library was measured by Quant-iT PicoGreen dsDNA Assay (ThermoFisher) and diluted to ∼4 nM. This concentration was further adjusted based on qPCR quantification (Kapa Biosystems). Samples were loaded onto an Illumina MiSeq using a v2 50-cycle kit set up to run a single-direction read of 50 nucleotides.

Data was automatically demultiplexed by MiSeq Reporter software and the resulting fastq files were processed by a custom Python script (Supplementary Note 2). This script searches each sequencing read for a perfect match to sequences flanking both sides of the proteolysis site. If the proteolysis site in between these matching flanks is exactly 21 nucleotides, then the proteolysis site is translated to a seven amino acid sequence. Sequences were disregarded in subsequent analysis if they contained a stop codon, were template material used in library cloning (HNLYGHS), or were the FLAG tag sequence (YKDDDDK) due to spontaneous genetic deletion of the proteolysis site. The list of proteolysis sites was tabulated into a position-specific amino acid frequency table. For each library-protease combination, enrichment values were calculated as freq_cleaved_/freq_elution_-1. For each protease, specificity data from the randomized position within single-site libraries was combined into a single table and converted into a sequence logo using a the Seq2Logo webserver^39^. For libraries containing windows of three randomized positions, sequence logos for each protease-library combination were separately generated.

### Western blot visualization of recombinant IL-23 cleavage

IL-23 was purchased as a Myc-tagged IL-23p19 monomer (TP309680 Origene) and as a heterodimer of IL-23p19 and IL12p40 (PHC9321 ThermoFisher). A total of 5 µg of heterodimer or 0.44 µg of monomer was incubated with 5 µg of TEV L2F for 3 h at 30 °C in storage buffer (20% glycerol, 50 mM Tris pH 8.0, 0.1 M NaCl, 1 mM DTT) supplemented with freshly prepared DTT to a final concentration of 2 mM. Samples were electrophoresed on precast Bolt 4–12% Bis–Tris gels (ThermoFisher), then transferred to a PVDF membrane using the iBlot 2 Dry Blotting System (ThermoFisher). Membranes were incubated at room temperature for 30 min in Odyssey Blocking Buffer (LiCor). Primary antibody (IL-23 Antibody (26H20L23), ABfinity Rabbit Monoclonal, ThermoFisher) was added to blocking buffer in 1:1000 dilution, and the membrane was incubated on a rocker at 4 °C overnight. After three washes with TBST (20 mM Tris pH 7.0, 150 mM NaCl, 0.1% Tween 20), the membrane was incubated 1 h at room temperature in blocking buffer containing a 1:1000 dilution secondary antibody (IRDye 800CW Donkey anti-Rabbit IgG, LiCor). After three more washes with TBST, the membrane was scanned using an Odyssey Imaging System.

### LC–MS identification of cleavage sites

IL-23p19 and the IL-23 heterodimer (ThermoFisher) were reduced with 10 mM DTT to identify intact masses of unreacted IL-23p19 subunits. IL-23 substrates were incubated in a manner similar to western blots for 3 h at 30 °C using 2–10 µg of substrate and 4 µg of TEV L2F. All samples were analyzed using an Agilent LC–MS 6220 (ESI-TOF) equipped with an Agilent PLRP-S column. A standard protein LC method was used containing a 15-min reverse-phase gradient (0.1% formic acid in water, MeCN 0.1% formic acid).

### IL-23-induced IL-17 production in mouse splenocytes

The following protocol was adapted from previous work^48^ as follows: two male mice (C57BL/6J) were euthanized and dissected to isolate spleens, under a protocol approved by the Broad Institute Institutional Animal Care and Use Committee. Spleens were pulverized into 10 ml of cell culture media (DMEM, Glutamax, high-glucose, penicillin, streptomycin, 10% Fetal Bovine Serum, FBS, ThermoFisher) through a 100 µm nylon mesh Falcon Cell Strainer (Corning). Cell suspensions were centrifuged for 3 min at 700 g, and the supernatant was discarded. The pellet was resuspended in 1 ml ACK lysis buffer (Gibco/ThermoFisher). After 5 min, lysis was stopped with the addition of 9 ml of DMEM, and cells were pelleted by centrifugation at 700 × *g* for 3 min. If the pellet was red due to remaining red blood cells, ACK lysis was repeated. Otherwise if the pellet was white, lysis was complete and cells were resuspended in 4 ml of DMEM. Cell density was quantified using a Scepter 2.0 Handheld Automated Cell Counter (Millipore). Cultures were diluted to 2 × 10^6^ cells per ml in cell culture media supplemented with 100 units per ml recombinant human IL-2 (Roche). The outer perimeter wells of a 96-well round bottom culture plate were filled with 100 µl of cell-free media to prevent evaporative loss in central wells containing cell cultures. The central wells were prepared in triplicate filled first with 125 µl of culture followed by 25 µl of additives (see below). Cell culture supernatant was sampled after two days of growth, and 10 µl was used to perform a mouse IL-17 ELISA (R&D Systems).

Additives containing IL-23 and varying doses of protease or neutralizing antibody (MAB1510, R&D Systems) were prepared in cell culture media immediately prior to mixing with splenocytes. Additives containing doses of proteases were also prepared as pre-incubated samples at 300× final concentration. Incubation was performed at 4 °C for 16 h in storage buffer (20% glycerol, 50 mM Tris pH 8.0, 0.1 M NaCl, 1 mM DTT) supplemented with 2.5 mg ml^−1^ of BSA carrier-protein to enhance stability during incubation. These pre-incubated samples were prepared at high concentration to confirm cleavage efficiency by western blot as described above. However, this western blot was conducted using two primary antibodies Anti-IL-12p40 (ab62822 Abcam)/Anti-IL-23p19 (sc271279 Santa Cruz) (dilutions were 1:500 and 1:100 respectively) and two secondary antibodies IRDye800CW Donkey anti-Mouse/IRDye 680RD Donkey anti-Goat (LiCor) (dilutions were 1:2000 each).

### Data availability

High-throughput sequencing data will be made available via the Sequence Read Archive under BioProject number PRJNA397152. DNA sequences and constructs are available from Addgene. Complete mutational tables are provided in Supplementary Information.

## Electronic supplementary material

Supplementary Information
