# Supplementary material for: Phage-assisted continuous evolution of proteases with altered substrate specificity
Source: Nat Commun. 2017 Oct 16;8:956. doi: 10.1038/s41467-017-01055-9 (PMC5643515; doi:10.1038/s41467-017-01055-9)
Supplement: Supplementary file 1 — Supplementary Information [file 41467_2017_1055_MOESM1_ESM.pdf]

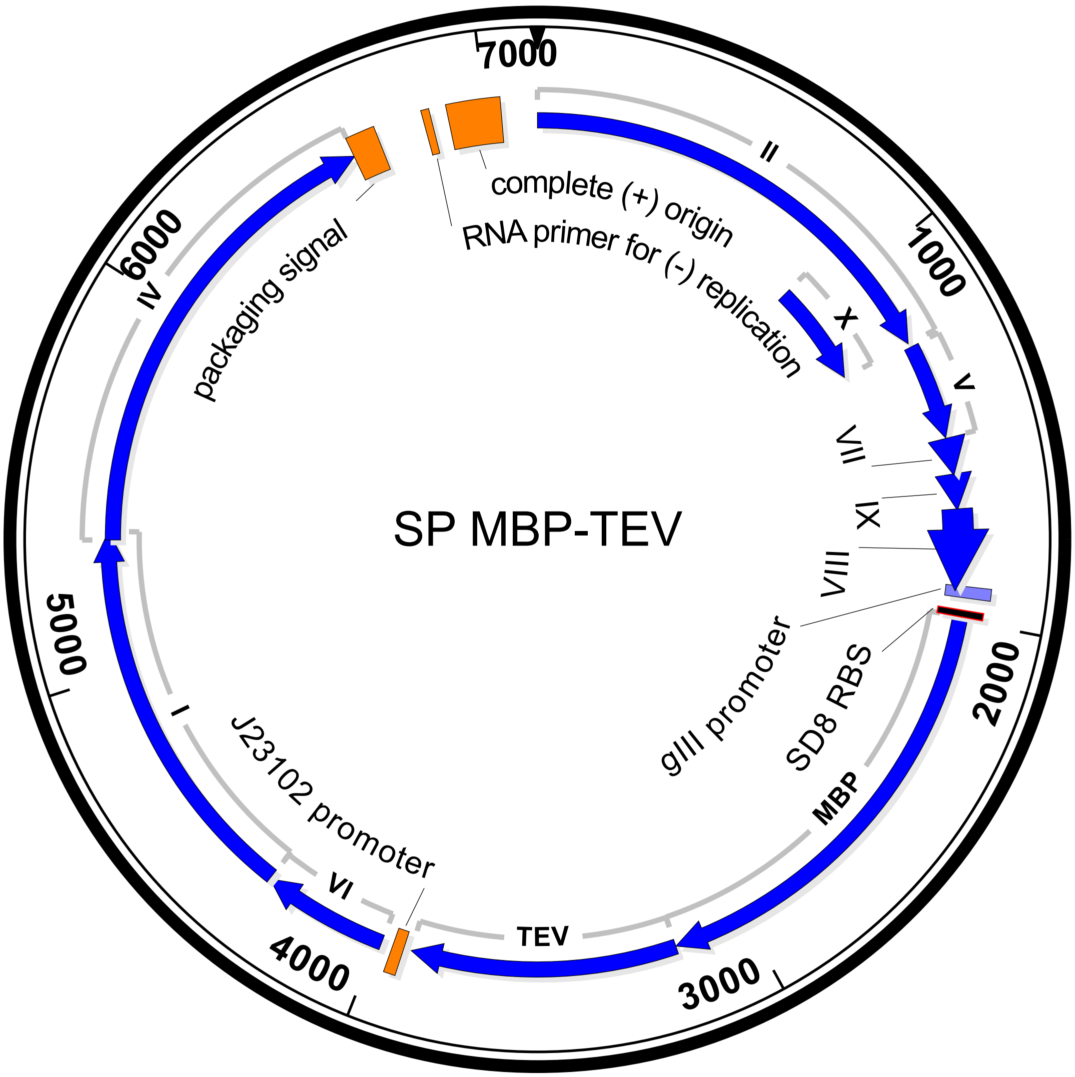

Supplementary Figure 1. Selection phage plasmid map. M13 bacteriophage gIII has been replaced with the evolving gene of interest, maltose-binding protein (MBP) fused to TEV through a GGS linker. The MBP fusion enhances soluble expression of active TEV protease. The gIII promoter has been left intact to drive expression MBP-TEV from an SD8 RBS. The gVI promoter, which is natively internal to the gIII coding sequence, has been replaced with a synthetic J23102 promoter.

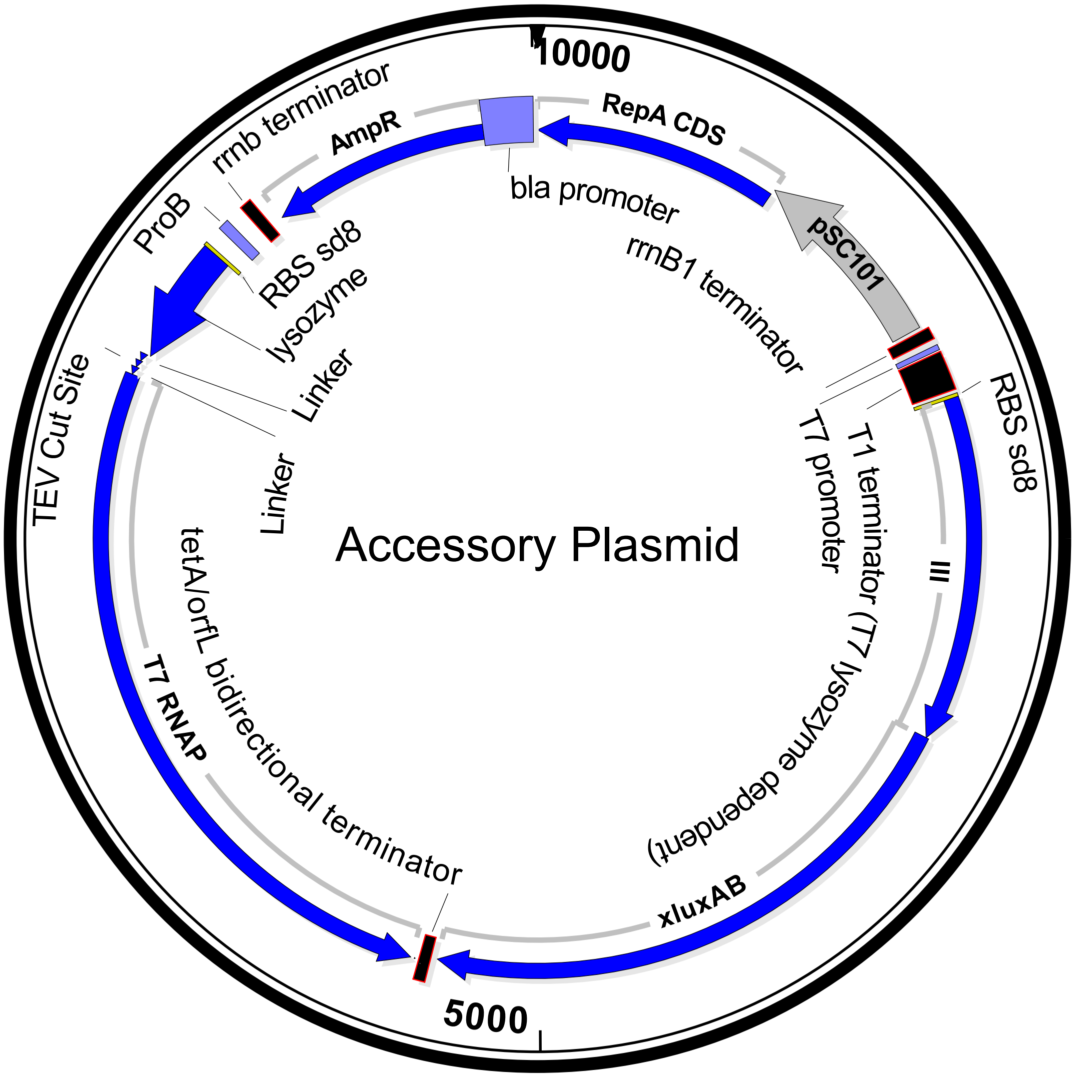

Supplementary Figure 2. Accessory plasmid map. A single accessory plasmid encodes for constitutive expression (proB promoter) of a PA-RNAP (Lysozyme-Linker-TEVcutsite-Linker-T7RNAP) as well as T7 promoter-controlled expression of gIII and the translationally coupled luciferase reporter (xluxAB). A lysozyme-dependent terminator (T1 terminator) is placed downstream of the T7 promoter to lower transcription of gIII-xluxAB in the absence of active protease. This plasmid encodes the low copy pSC101 origin of replication and is maintained with carbenicillin.

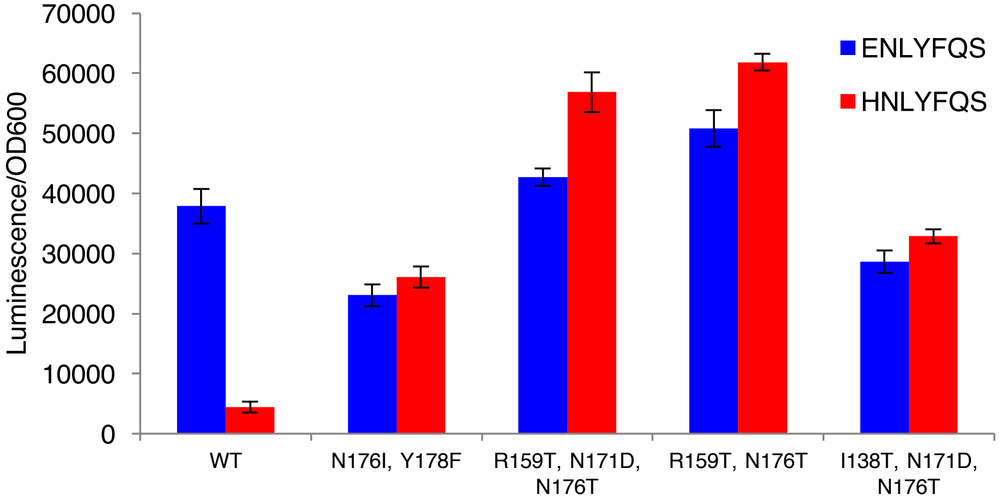

Supplementary Figure 3. Luciferase activity assay of clones from the middle of PACE stage 1 of trajectories 1, 2, and 3. TEV protease clones (corresponding genotypes are shown beneath the x-axis) after 36h of evolution on the first stepping-stone substrate show apparent proteolytic activity on both the wild-type substrate and the single mutant substrate HNLYFQS. Center values represent the mean and error bars represent the standard deviation of three technical replicates.

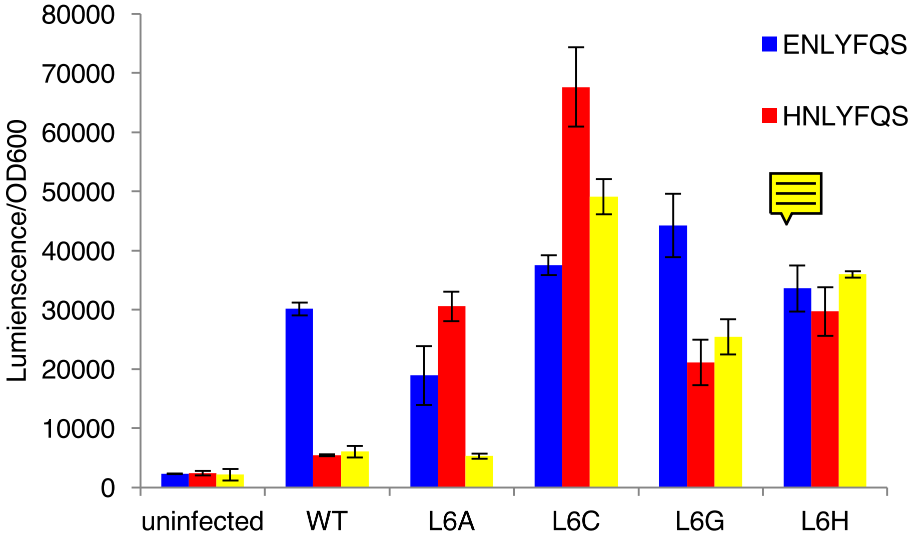

Supplementary Figure 4. Luciferase activity assay after PACE stage 2 of trajectories 1 and 2. TEV protease clones from trajectories 1 and 2 (corresponding genotypes can be found in Supplementary Table 4) after evolution on the second stepping-stone substrate, ENLYGQS, show activity on the wild-type substrate (ENLYFQS; blue bars) and both single mutant substrates (HNLYFQS/ENLYGQS; red and orange bars, respectively). Center values represent the mean and error bars represent the standard [illegible]ation of three technical replicates.

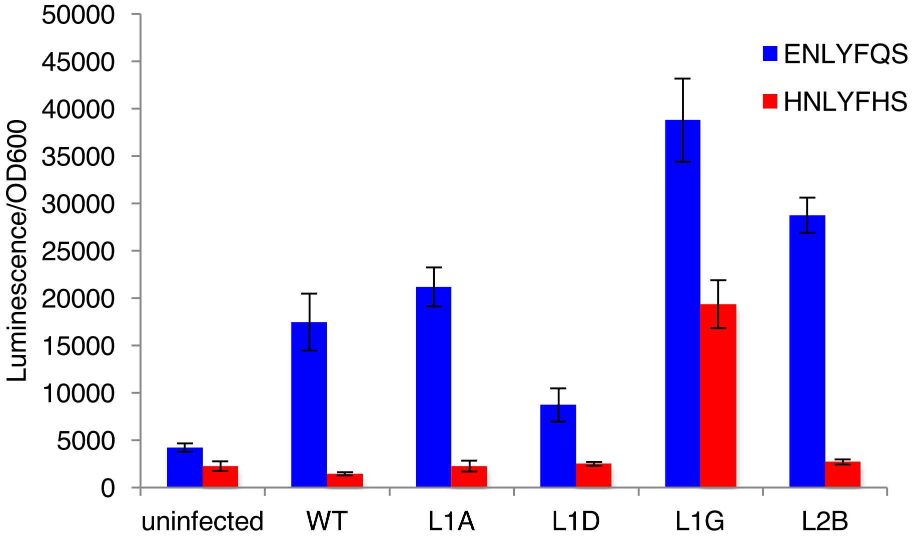

Supplementary Figure 5. Luciferase activity assay after PACE stage 2 of trajectory 3. TEV protease clones from trajectory 3 (corresponding genotypes can be found in Supplementary Table 5) after evolution on the second stepping-stone substrate, HNLYFHS, show apparent activity on the wild-type substrate and the double mutant substrate, HNLYFHS. Center values represent the mean and error bars represent the standard deviation of three technical replicates.

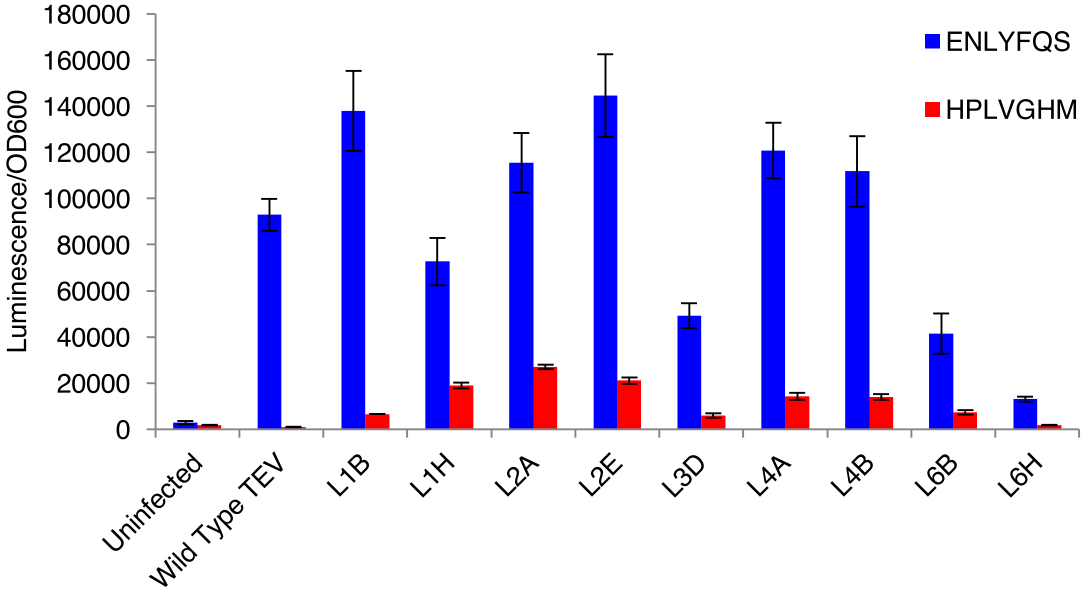

Supplementary Figure 6. Luciferase activity assay of clones after PACE stage 4. PACE evolved TEV SP clones (corresponding genotypes can be found in Supplementary Table 7) from stage four of the evolutionary trajectories show proteolysis of HPLVGHM substrates within a protease-activated RNA polymerase as measured by downstream luciferase signal. These data indicate that the evolved enzymes were acquiring the desired phenotype, but higher selection stringency would be necessary in order to achieve catalytic activity similar to that of wild-type TEV protease. Center values represent the mean and error bars represent the standard deviation of three technical replicates.

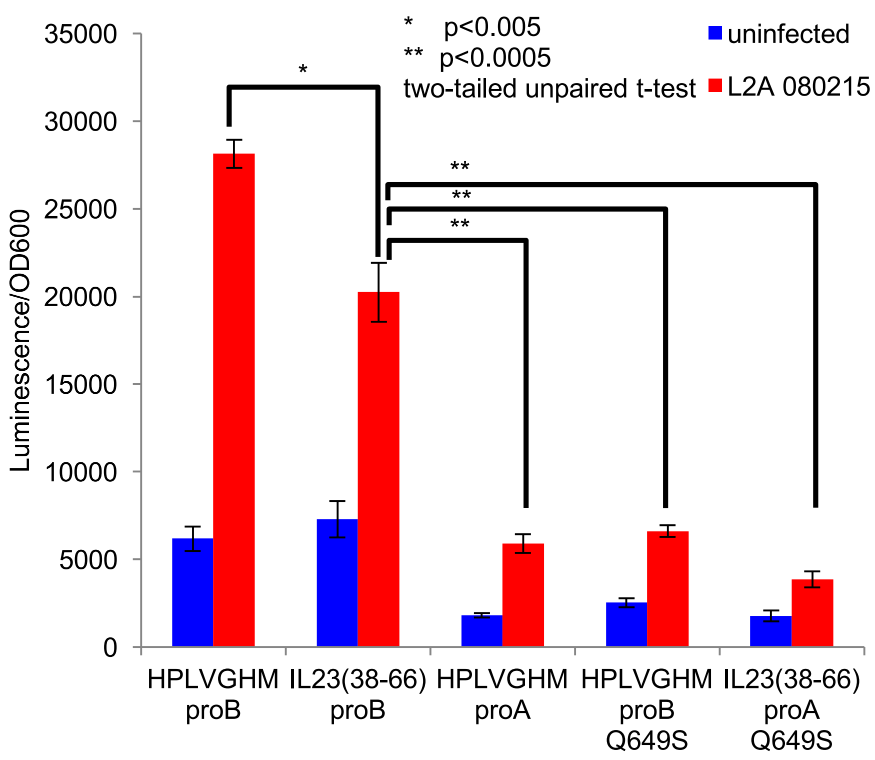

Supplementary Figure 7. Validation of stringency modulation. Using the highest activity TEV variant prior to stringency modulation in PACE, protease-induced luminescence assays were conducted using APs that were expected to exert higher selection stringency. Prior to stringency modulation, the HPLVGHM proB AP exhibits robust protease-induced luminescence and 4.7-fold fold activation. Replacement of the flexible GGS-linkers in the PA-RNAP with amino acids 38-66 of IL-23 preserves the relative position of the cleaved peptide bond within the substrate linker, but due to the altered secondary structure of the substrate in this sequence context, the protease-induced luminescence is diminished (2.8-fold activation). When expression levels of the HPLVGHM PA-RNAP are lower due to a weaker constitutive promoter (proA instead of proB), we see much lower background and protease-induced luminescence as well as a 3.3-fold activation. The introduction of deactivating mutation Q649S to the T7 RNAP portion of the PA-RNAP also causes a decrease in background and protease-induced luciferase signal (2.7-fold activation). When all three strategies are combined in a single AP, an even greater decrease in luciferase signal is observed (2.1-fold activation). Lower fold activation corresponds with higher selection stringency. Center values represent the mean and error bars represent the standard deviation of three technical replicates.

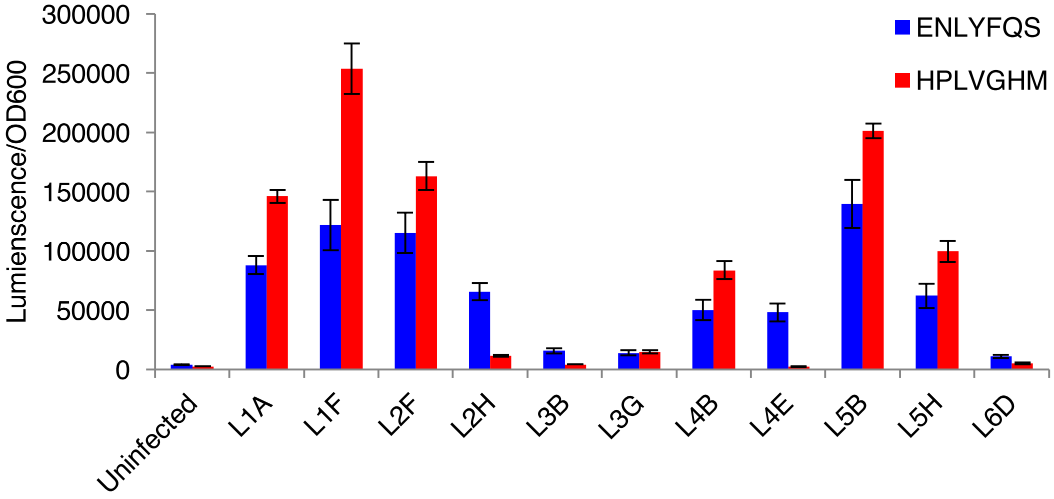

Supplementary Figure 8. Luciferase activity assay of clones after PACE stage 8. After multiple PACE stages with increasing positive selection stringency, many TEV protease variants (corresponding genotypes can be found in Supplementary Table 11) exhibit markedly stronger apparent activity on the HPLVGHM substrate when compared with clones from previous PACE experiments such as those seen in F[illegible]4. Center values represent the mean and error bars represent the standard deviation of three technical replicates.

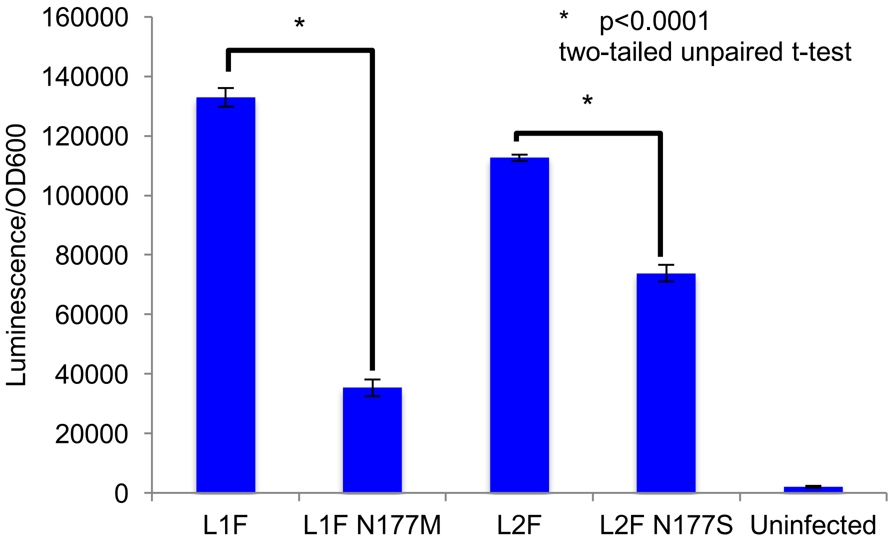

Supplementary Figure 9. Epistatic interactions with TEV protease residue N177. PACE evolved clones L1F and L2F, from [illegible]E 8 of trajectories 1 and 2 respectively, exhibit robust apparent activity on the HPLVGHM substrate. When the identity of residue N177 is swapped between these clones, as in variants L1F N177M and L2F N177S, a statistically significant decrease (p-value <10^-4) in apparent activity is observed, suggesting that the optimal substitution for N177 depends upon the identities of other mutations within TEV protease. Center values represent the mean and error bars represent the standard deviation of three technical replicates.

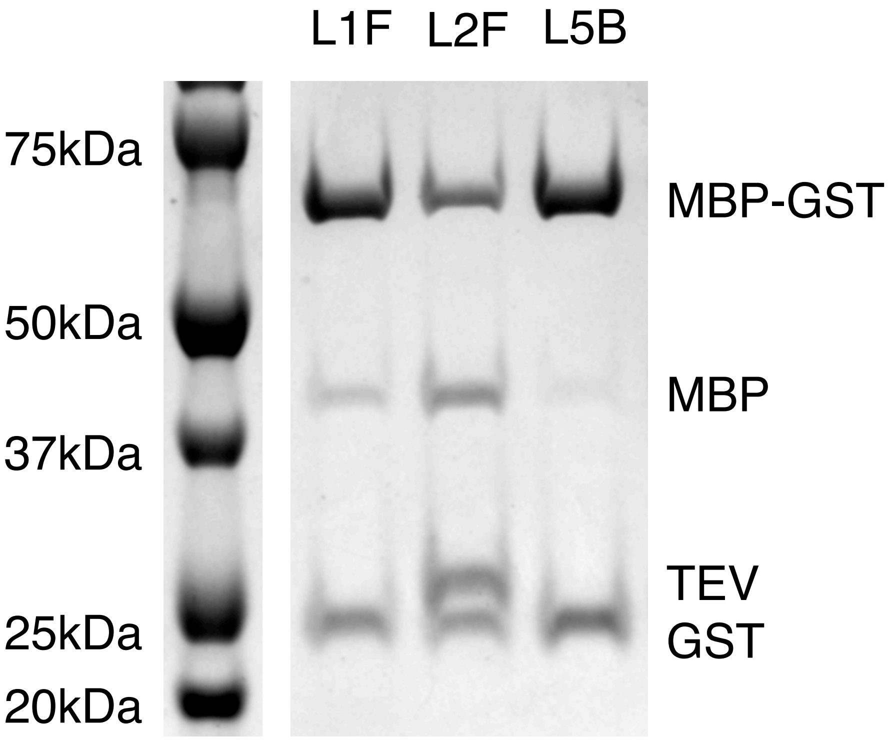

Supplementary Figure 10. Protein cleavage assay to identify the most active clone. TEV protease variants from the final PACE experiment were overexpressed and purified. Approximately 1 μg of protease was incubated for 3 h at 30°C with 5 μg of a fusion protein construct in which MBP is linked to GST through a cleavable substrate linker containing the peptide HPLVGHM. Note that TEV protease variants L1F and L5B encode premature stop codons leading to products with approximately the same molecular weight as GST. Consequently, the intensity of the MBP product band best reflects reaction efficiency, leading to the conclusion that TEV L2F exhibits the highest catalytic activity among the clones tested.

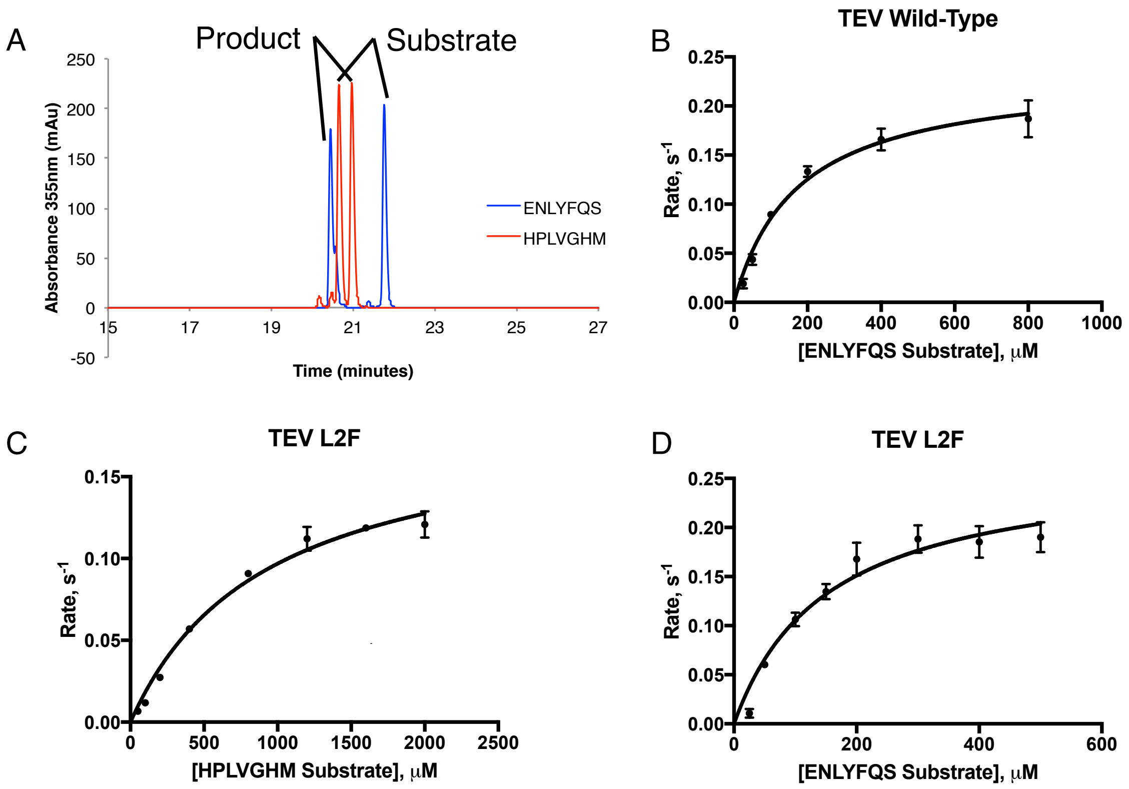

Supplementary Figure 11. HPLC assay of TEV protease kinetics. (A) Synthetic peptide standards. TEV protease substrate peptides and the corresponding product peptides in a 1:1 mixture are separable by reverse-phase liquid chromatography. (B) Wild-type TEV protease (0.1 μM) was incubated for 10 min at 30 °C with ENLYFQS substrate concentration ranging from 50 to 800 μM. (C) TEV L2F protease (0.1 μM) was incubated for 10 min at 30 °C with HPLVGHM substrate concentration ranging from 50 to 2000 μM. (D) TEV L2F protease (0.05 μM) was incubated for 10 min at 30 °C with ENLYFQS substrate concentration ranging from 50 to 500 μM. Data was fit to a Michaelis-Menten enzyme kinetics model with center values representing the mean and error bars representing the standard deviation of three technical replicates.

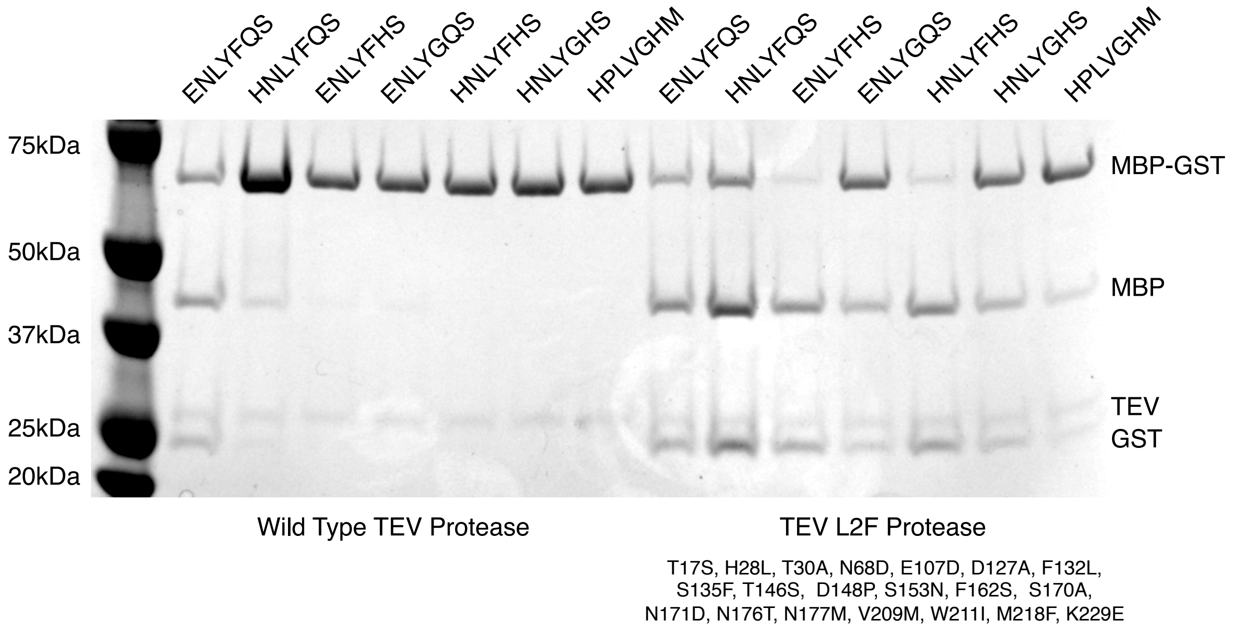

Supplementary Figure 12. Evolved TEV protease cleaves wild-type, intermediate, and target substrates. In a manner analogous to that described above in Supplementary Figure 10, we assayed TEV proteases on a panel of substrate sequences. Approximately 1 μg of protease was incubated for 3 h at 30°C with 5 μg of a fusion protein construct in which MBP is linked to GST through a cleavable substrate linker containing the indicated amino acid sequence. WT TEV efficiently cleaves wild-type substrate, and to a much lesser degree processes single mutant substrates (HNLYFQS, ENLYFHS, ENLYGQS). Evolved TEV protease clone L2F yields a visible product band for the target substrate HPLVGHM. This evolved protease has also maintained activity on wild-type, single, double, and triple mutant substrates that were used as evolutionary stepping-stones in PACE.

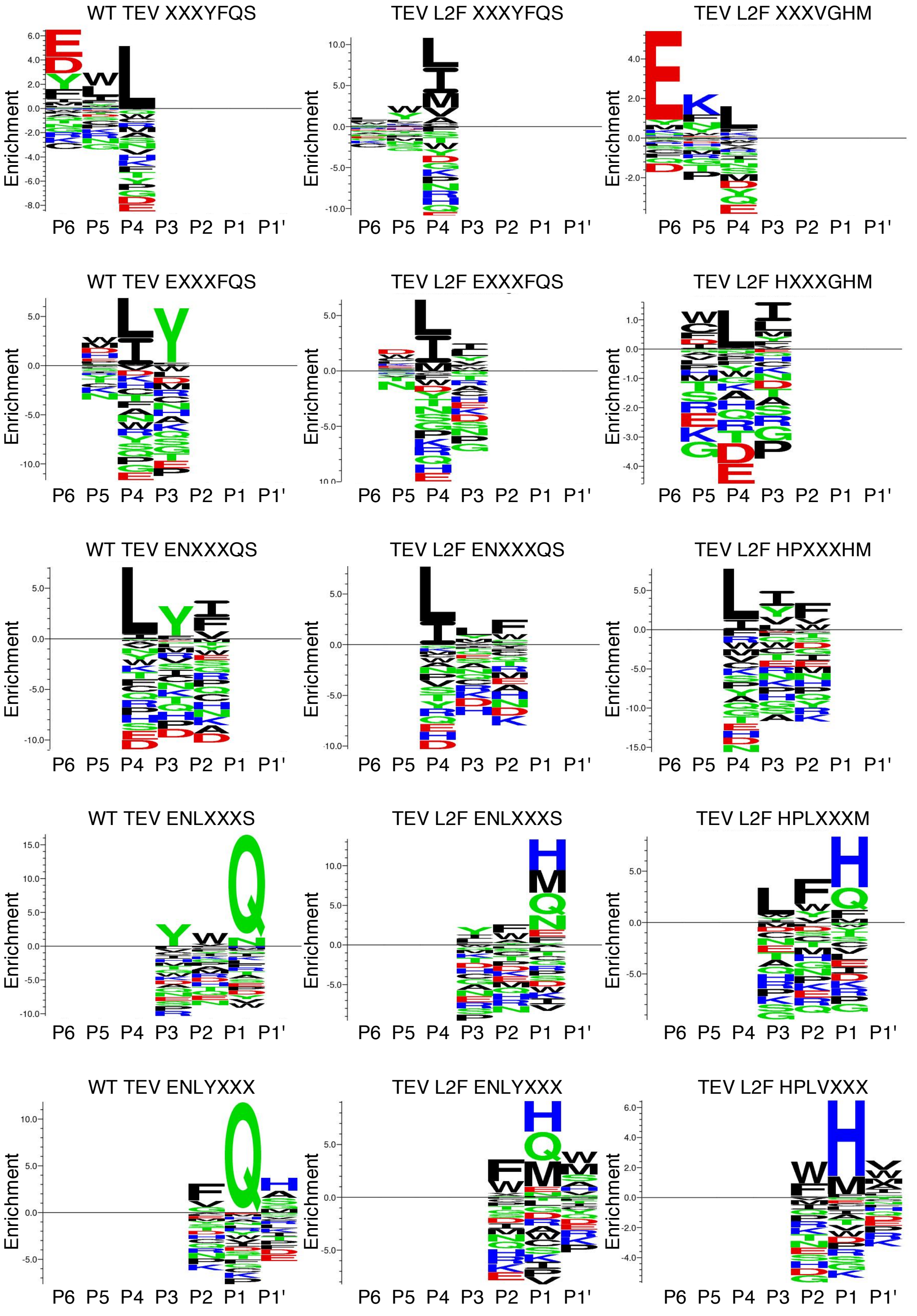

Supplementary Figure 13. Specificity profiles generated from libraries with three randomized substrate amino acids. The logos above were generated using phage substrate

libraries containing windows of three randomized amino acids within either the ENLYFQS or the HPLVGHM substrate (corresponding enrichment values in Supplementary Table 13). The nature of the library and the protease that was used in the selection is specified in the title above each sequence logo (with X denoting randomized substrate residues). Letter height is proportional to enrichment in the cleaved versus eluted sample. Letters placed above the x-axis indicate protease acceptance and letters beneath the axis indicate rejection. The specificity profiles of wild-type and evolved TEV L2F protease using three-residue ENLYFQS libraries are largely similar to those seen in Figure 3 using single-site randomized substrate libraries. In the context of the HPLVGHM libraries, however, we observe that TEV L2F exhibits greater specificity for glutamate at P6 and for histidine at P1.

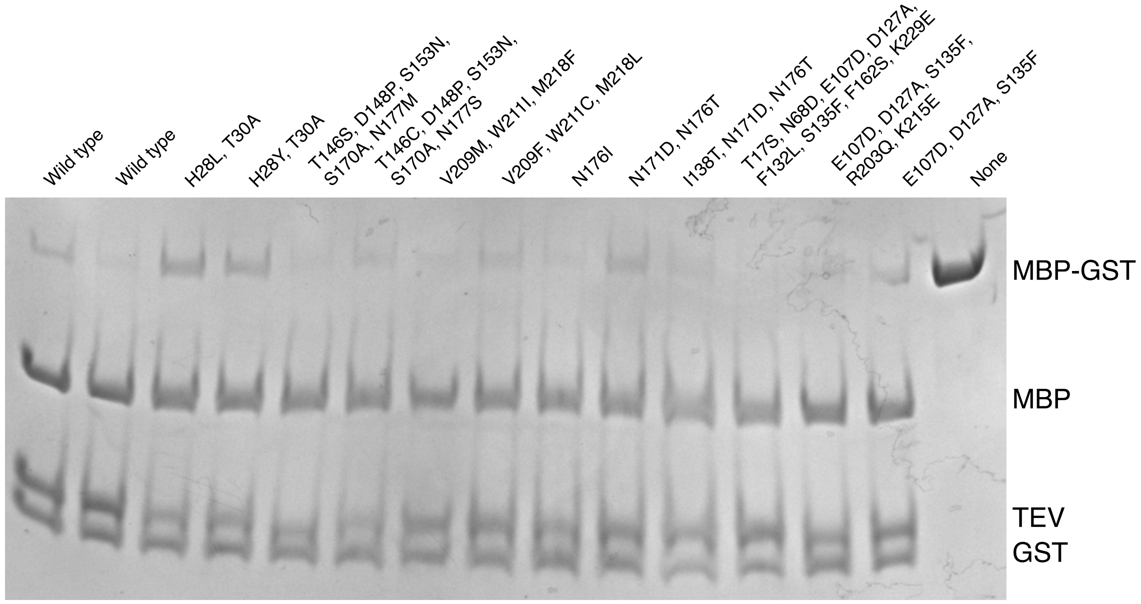

Supplementary Figure 14. TEV protease variants containing subsets of TEV L2F mutations are all active. TEV protease variants were engineered to contain groups of mutations taken from the L2F variant. These enzymes were purified and assayed *in vitro* on the test substrate, MBP–GST, containing the wild-type substrate motif ENLYFQS in the linker. Approximately 1 µg of protease was incubated for 3 h at 30°C with 5 µg of a fusion protein construct in which MBP is linked to GST through a cleavable substrate linker containing the ENLYFQS peptide. All assayed variants retained proteolytic activity despite the naïve genetic dissection of mutations.

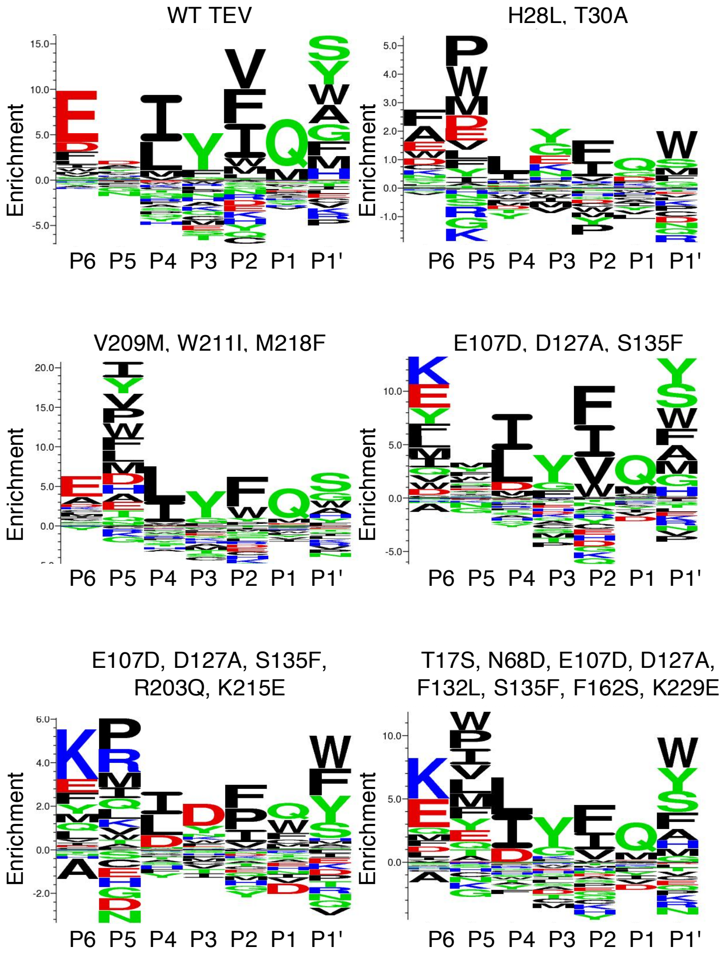

Supplementary Figure 15. Specificity profiles of TEV variants. The logos above were generated using phage substrate libraries each containing a single randomized amino acid within the ENLYFQS substrate (corresponding enrichment values in Supplementary Table 12). The genotype of the protease that was used in the selection is specified in the title above each sequence logo. Letter height is proportional to enrichment in the cleaved versus eluted sample. Letters placed above the x-axis indicate protease acceptance and letters beneath the axis indicate rejection. The above proteases demonstrate strong enrichment of the wild-type consensus residue at all seven individual positions, but certain variants exhibit broadened specificity and allow, for example, Lys at P6 or Asp at P3.

A

-	5	-	5	TEV L2F (µg)
5	5	-	-	IL-23 (µg)
-	-	0.45	0.45	IL-23p19 (µg)

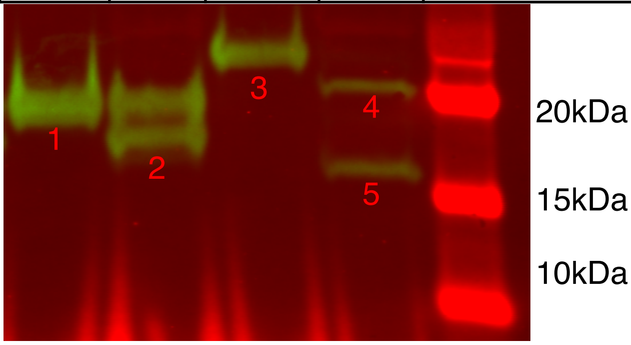

B RAVPGGSSPAWTQCQQLSQKLCTLAWSA
HPLVGH_MDLREEGDEETTNDVPHIQCGD
GCDPQGLRDNSQFCLQRIHQGLIFYEKLL
GSDIFTGEPSLLPDSPVGQLHASLLGLSQL
LQPEGHHWETQQMPSLSPSQPWQRLLLR
FKILRNLQAFVAVAARVFAH_GAATLSP

Supplementary Figure 16. Identification of IL-23 cleavage sites by Western blot and LC-MS. IL-23 heterodimer (IL-23) and IL-23 monomer (IL-23p19) were incubated with and without TEV L2F. Reaction mixtures were subject to LC-MS and (A) visualized by western blot with anti-IL-23p19 monoclonal antibody. Bands 1 and 3 correspond to intact IL-23p19; differences in size are due to carboxy-terminal affinity purification tags. Cleavage product bands 2 and 4 correspond to IL-23 fragments with new masses that are 3,598 Da less than the corresponding starting materials. This mass difference is a perfect match for the fragment liberated by cleavage at the target site (HPLVGH//M). Cleavage of the monomer also results in a second product (band 5) with a mass that matches IL-23 cleaved at both the target site (HPLVGH//M) and an off-target site (ARVFAH//G). (B) The IL-23p19 amino acid sequence is shown with the target cleavage site in blue and the off-target site in orange.

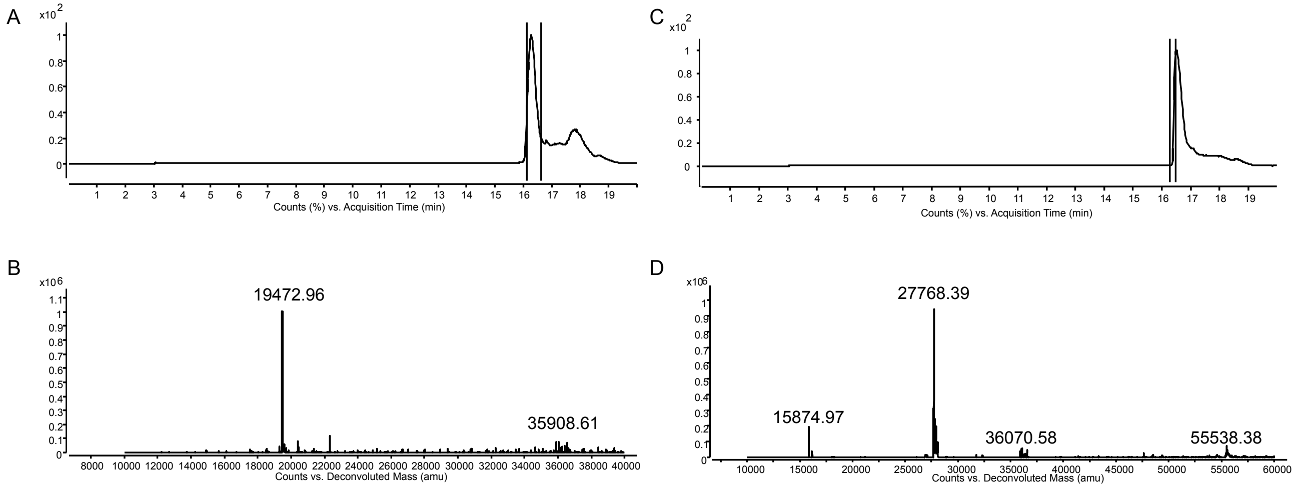

Species	Expected Mass (Da)	Observed Mass (Da)	Delta (Da)	Error (%)
IL12-IL23	55891.2	55538.38	352.82	0.631
IL12	37168.97	36070.58	1098.39	2.955
IL23	18722.23	19472.96	-750.73	-4.010
TEV L2F	27711.63	27768.04	-56.41	-0.204
IL23 HPLVGH/M	15124.11	15874.97	-750.86	-4.965

Supplementary Figure 17. Identification of the cleavage site within IL-23 heterodimer by mass spectrometry. IL-23 was obtained in its native heterodimeric state following expression and purification from cultured mammalian cells (PHC9321, ThermoFisher). Ten micrograms of this protein (final concentration 6.0µM) was incubated for 3 h at 30°C under reducing conditions (5mM DTT) either in the presence or absence of TEV L2F (4µg; final concentration 4.8µM). These samples were analyzed by LC-MS to yield total ion current (A, C) and the corresponding deconvoluted mass spectra (B, D). Both samples exhibit a cluster of masses around 36,000 Da corresponding to the multiple glycoforms of the IL12p40 subunit. The unreacted sample (A, B) contains a mass of 19,472Da that is 751 Da greater than the expected mass of IL-23, which is likely caused by an unspecified proprietary C-terminal tag. The reaction mixture (C, D) contains a 27,768 Da match for TEV L2F as well as a 15,875 Da mass that matches the expected cleavage product plus the unspecified 751 Da C-terminal tag.

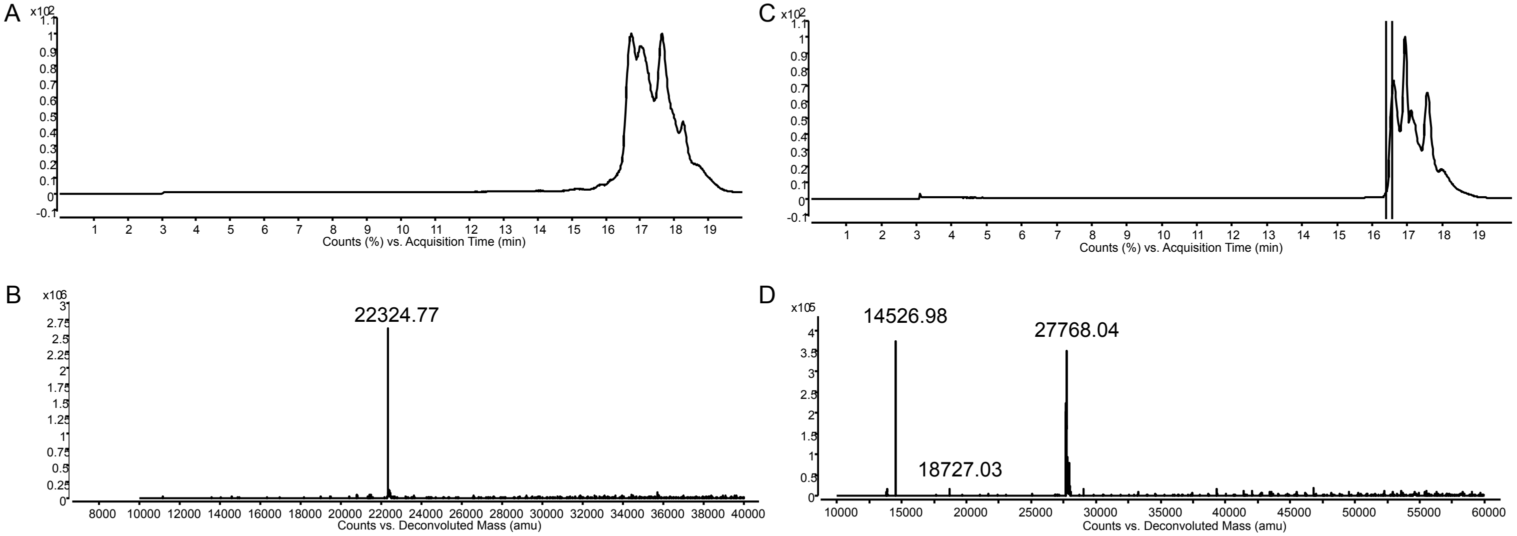

Species	Expected Mass (Da)	Observed Mass (Da)	Delta (Da)	Error (%)
IL23	22324.16	22324.77	-0.61	-0.003
TEV L2F	27711.63	27768.04	-56.41	-0.204
IL23 HPLVGH/M	18726.04	18727.03	-0.99	-0.005
IL23 HPLVGH/M, ARVFAH/G	14526.[illegible]	[illegible]4526.98	-0.54	-0.004

Supplementary Figure 18. Identification of two cleavage sites within IL-23 monomer by mass spectrometry. IL-23p19 was expressed and purified from cultured HEK293T cells using a C-Terminal Myc/DDK tag (TP309680, Origene). Two micrograms of this protein (final concentration 3.0µM) was incubated for 3 h at 30°C under reducing conditions (5mM DTT) either in the presence or absence of TEV L2F (4µg; final concentration 4.8µM). These samples were analyzed by LC-MS to yield total ion current (A, C) and the corresponding deconvoluted mass spectra (B, D). The unreacted sample (A, B) contains a mass of 22,324 Da which is a perfect match for the IL-23p19 sequence and Myc tag in the product data. The reaction mixture (C, D) contains three additional masses: TEV L2F (27,768 Da), substrate cleaved only at the HPLVGHM target site (18,727 Da), and substrate cleaved at both the target site and an off-target site ARVFAHG (14,526 Da).

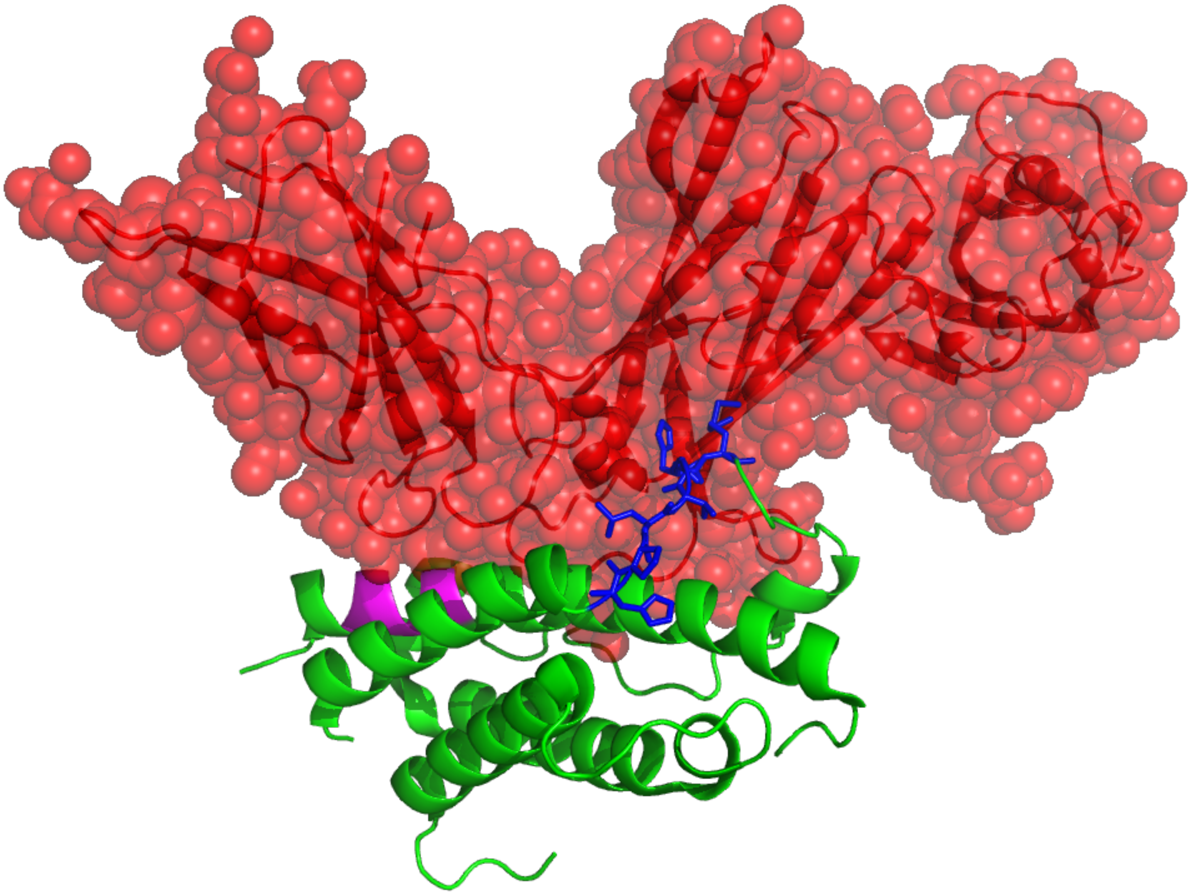

Supplementary Figure 19. Crystal structure of the IL12-IL23 complex. The heterodimer of IL12p40 and IL23p19 has been previously characterized by X-ray crystallography (PDB entry 4OE8; Desmet *et al.* (2014) *Nat. Comm.* **5**, 5237). In the above rendering, IL12p40 is shown in red and IL23p19 is shown in green. The target cleavage site (HPLVGH/M, shown in blue) is surface accessible, thus permitting proteolysis even in the presence of IL12p40. This structure also demonstrates that the secondary cleavage site (magenta) is occluded at the interface with IL12p40, thus providing a likely explanation for why proteolysis at this site is only observed when IL23p19 is present as a monomer.

IL23 (0.54µM)	-	-	-	+	+	+	+	+
TEV	-	L2F 36µM	WT 36µM	-	L2F 3.6µM	L2F 18µM	L2F 36µM	WT 36µM

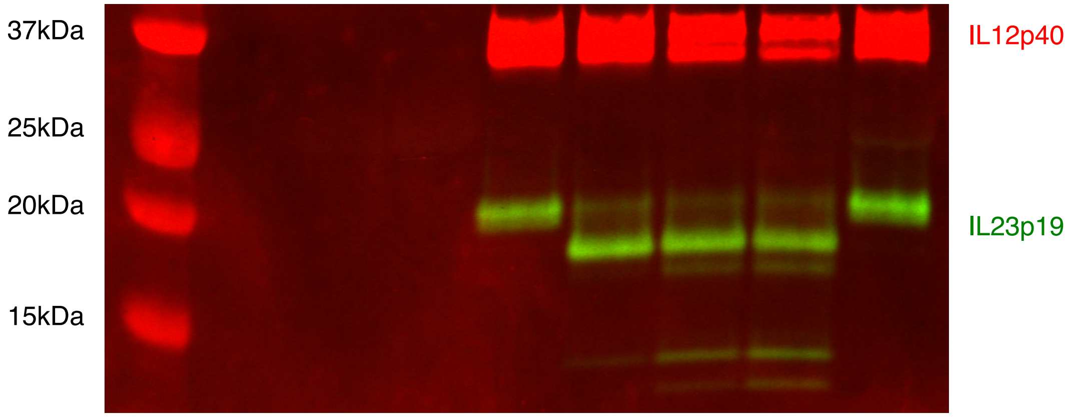

Supplementary Figure 20. Western blot of pre-mixed additives to splenocyte cell culture. IL-23 and TEV proteases were incubated for 16 h at 4 °C in the presence of BSA (bovine serum albumin) as a stabilizing carrier protein. Samples were prepared at 300x concentration used in splenocyte cultures to enable detection of IL-23p19 and IL-12p40 by western blot. Neither component is proteolyzed by wild-type TEV protease; IL-12p40 is also unaffected by TEV L2F. As expected, TEV L2F cleaves IL-23p19 at the HPLVGHM site in a dose-dependent manner. At the highest doses, off-target cleavage products are also observed. An aliquot of these samples was directly used in the cell culture experiments in Fig. 4 to confirm that on-target proteolysis causes IL-23 loss of function.

IL23 (0.54µM)	+	+	+	+
TEV	-	L2F 0.22µM	L2F 2.2µM	L2F 22µM

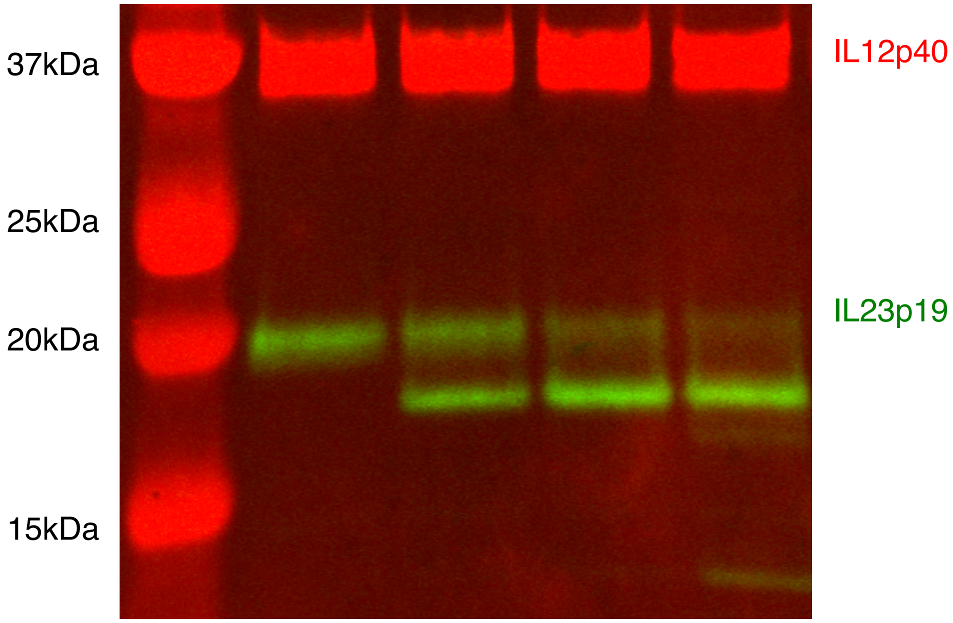

Supplementary Figure 21. Western Blot of pre-mixed additives to splenocyte cell culture. At approximately 0.40 molar equivalents of TEV L2F, greater than 50% of IL-23p19 is cleaved at HPLVGHM site, consistent with the ability of TEV L2F to process substrates with multiple turnover. At the highest doses off-target cleavage products are also observed. An aliquot of these samples was directly used in the cell culture experiments in Fig. S21 to confirm that on-target proteolysis causes IL-23 loss of function.

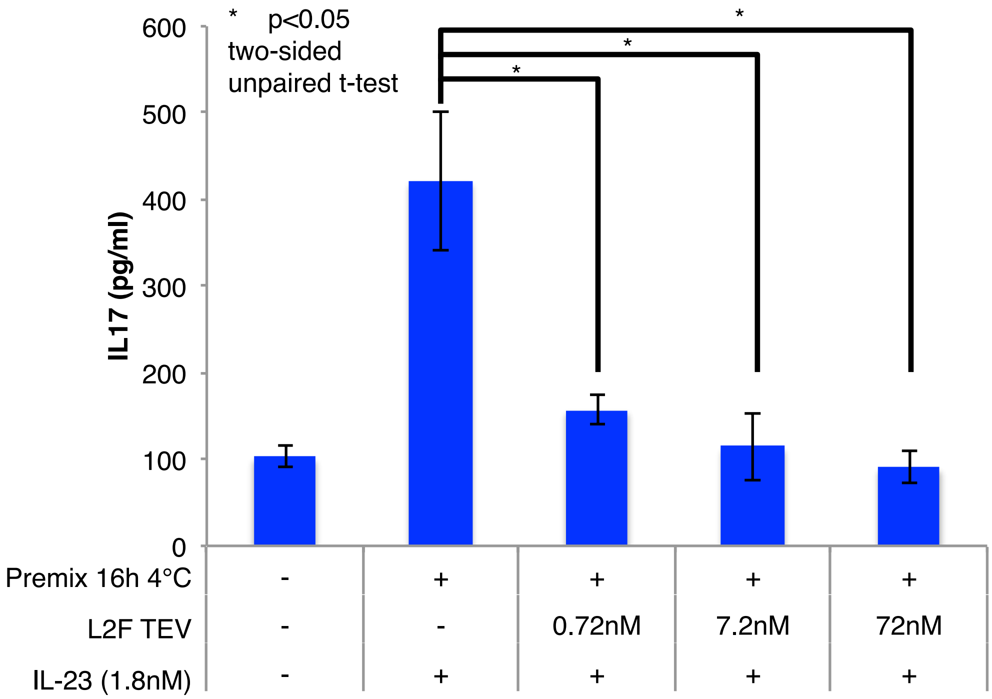

Supplementary Figure 22. TEV L2F catalytically deactivates IL-23 and prevents IL-17 secretion in mouse splenocytes. IL-17 is secreted by cultured mouse mononuclear splenocytes in response to human IL-23 in the media. The secretion of IL-17 can be prevented by pretreatment of IL-23 with TEV L2F at a dose that is less half the molar equivalent of IL-23. Inhibition is first observed at a dose corresponding with 0.72 nM TEV L2F (compared with 1.8 nM IL-23). Center values represent the mean and error bars represent the standard deviation of three technical replicates.

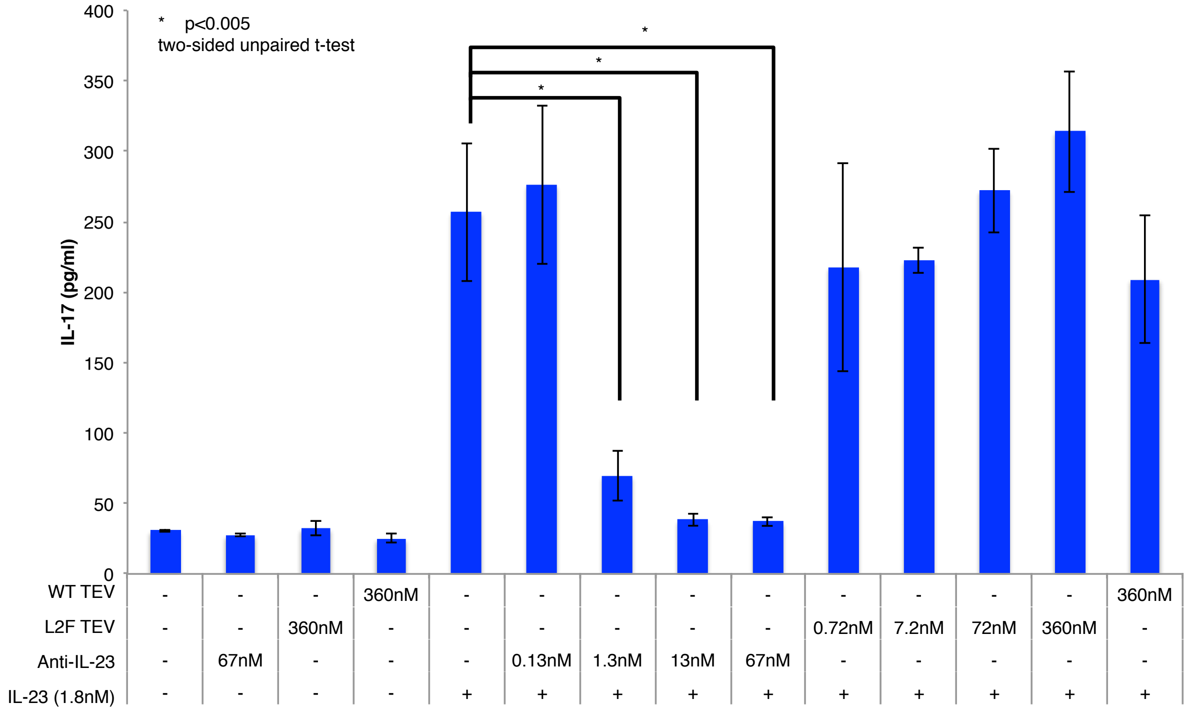

Supplementary Figure 23. IL-23 induced IL-17 secretion in mouse splenocytes. IL-17 is secreted by cultured mouse mononuclear splenocytes in response to human IL-23 in the media. This response can be prevented by addition of antibodies that neutralize IL-23 directly to cell culture media. A dose-dependent response is observed in which the antibody neutralizes IL-23 beginning at a dose of approximately 1.3 nM antibody (compared with 1.8 nM IL-23). Evolved TEV L2F, when added directly to cell culture media, is unable to prevent IL-23 from stimulating IL-17 secretion, due to slower kinetics of IL-23 degradation by protease compared with IL-23 receptor binding, the ~~more~~ oxidizing environment of the media, or sequestration of TEV L2F by the many other proteins in the media. Furthermore, the proteolysis reaction velocity at these physiological concentrations will be orders of magnitude slower than those observed in the 300-fold pre-incubation experiments (Fig. S21). Alternatively, it is also possible that the protease is sequestered by other cell surface or secreted factors preventing IL-23 proteolysis. Center values represent the mean and error bars represent the standard deviation of three technical replicates.

FBS 10%	-	-	-	-	-	+
IL23 (0.54µM)	+	+	+	+	+	+
TEV	-	L2F 0.22µM	L2F 2.2µM	L2F 22µM	WT 22µM	L2F 22µM

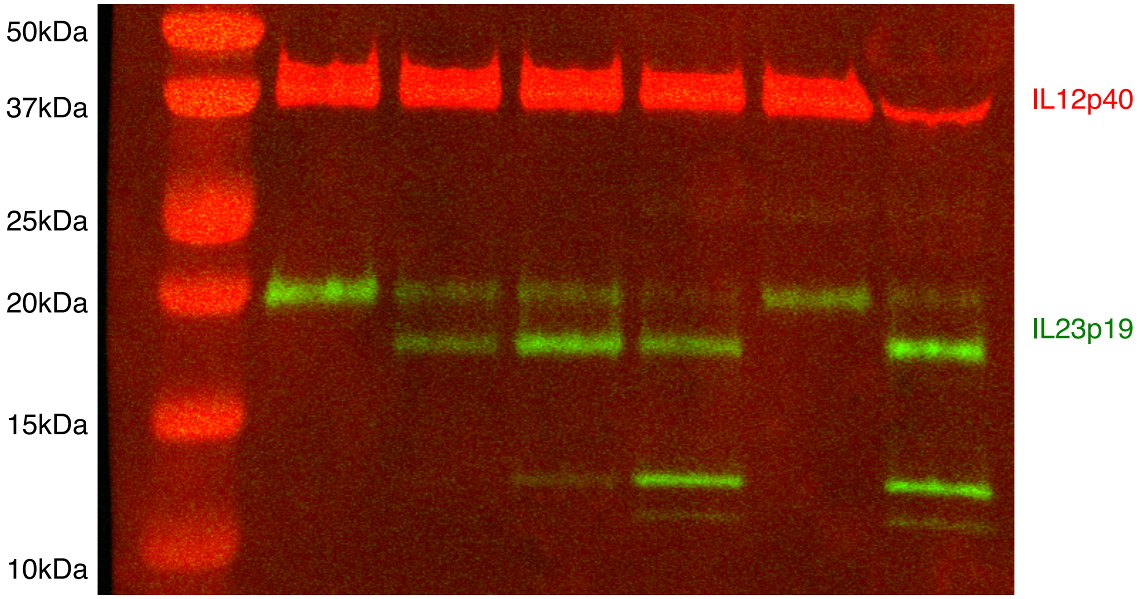

Supplementary Figure 24. TEV L2F is unaffected by the addition of FBS to *in vitro* cleavage assays.

IL-23 and TEV proteases were incubated for 16 h at 4°C in the presence of BSA as a stabilizing carrier protein. The addition of 10% Fetal Bovine Serum (FBS) to the assay buffer had no effect on the efficiency of cleavage by TEV L2F. The same percentage of FBS was used to supplement cell culture media, suggesting that components within serum are not responsible for a loss of TEV L2F activity when added directly to splenocyte cell cultures.

amino acid	P6	P5	P4	P3	P2	P1	P1'
A	-1	0	1	0	2	-1	0
R	0	0	0	-1	0	0	0
N	0	0	0	0	0	0	0
D	0	0	1	0	0	0	0
C	-1	-1	-1	-1	-1	-1	-1
Q	0	0	0	0	0	5	0
E	3	0	0	0	0	4	0
G	-1	0	1	0	0	-1	0
H	3	0	1	0	0	4	0
I	-1	0	1	0	1	-1	0
L	-1	0	1	0	1	-1	0
K	0	0	0	1	0	0	0
M	0	0	0	0	0	0	0
F	-1	0	0	0	3	-1	0
P	-1	0	-1	-1	-1	-1	-1
S	0	0	1	0	1	0	0
T	0	0	1	0	1	0	0
W	0	0	0	0	0	-1	0
Y	0	0	0	4	0	0	0
V	-1	0	1	1	1	-1	0

Supplementary Table 1. Target peptide scoring matrix. We created a subjective rating matrix based upon our knowledge of TEV protease substrate specificity and evolution of TEV proteases that accept single substrate changes. Key features include high ratings for consensus residues ENLYFQS as well as substitutions with known evolutionary solutions such as P6 His, P1 His, or P1 Glu. We also introduced penalties for Cys residues due to disulfide formation in mammalian target proteins and for Pro due to its unique structural properties.

TEV site	E	X	L	Y	F	Q	S
IL2RA	H	F	V	V	G	Q	M
IL23A	H	P	L	V	G	H	M
SELE	H	L	V	A	I	Q	N
SAA1/2	S	D	K	Y	F	H	A

Black = Matches consensus

Green = Previously evolved

Yellow = Accepted

Red = Requires evolution

Supplementary Table 2. Refined list of protease target substrates. Target substrates were identified from the human extracellular proteome based upon ratings calculated using the above scoring matrix. These four substrates were manually curated based upon the disease relevance of the target protein and the solvent-accessibility of target peptide.

	Trajectory		S3	N12	V36	S120	D127	F132	S135	I138	R159	N171	N176	E230
		A										N171D	N176T	
		B									R159I	N171D	N176T	
		C				S120N							N176I	E230A
		D					D127A		S135F				N176I	
		E					D127A		S135F				N176I	
		F					D127A	F132I	S135F				N176I	
		G					D127A		S135F				N176I	
L1		H					D127A		S135F				N176I	
		A	S3R							I138T		N171D	N176T	
		B		N12T						I138T		N171D	N176T	
		C								I138T		N171D	N176T	
		D								I138T		N171D	N176T	
		E								I138T		N171D	N176T	
		F								I138T		N171D	N176T	
		G								I138T		N171D	N176T	
L2	1,2,3	H			V36I					I138T		N171D	N176T	

Supplementary Table 3. Clonal sequencing data from the end of PACE stage 1 in Fig. 2~~A~~ (84 cumulative hours of evolution). Each row corresponds to a single clonal sequence of TEV protease from the SP. Lagoons 1 and 2 (L1 and L2) correspond to two separate biological replicate populations that underwent the same selection in PACE stage 1.

	Trajectory		Y11	K65	T70	T114	D127	S135	N176	V209	W211	V216	M218
		A			T70C	T114P	D127A	S135F	N176I	V209A	W211W	V216I	M218F
		B							N176I	V209M	W211V	V216V	M218W
		C					D127A	S135F	N176I	V209M	W211I	V216V	M218F
		E		K65R			D127A	S135F	N176I	V209E	W211L	V216I	M218W
		F	Y11C				D127A	S135F	N176I	V209M	W211I	V216V	M218W
		G					D127V	S135F	N176I	V209E	W211L	V216I	M218W
L6	1,2	H					D127A	S135F	N176I	V209M	W211I	V216V	M218W

Supplementary Table 4. Clonal sequencing data from trajectories 1 and 2 after PACE stage 2 in Fig. 2~~A~~ (168 cumulative hours of evolution). Lagoon six was inoculated with a site-saturation mutagenesis library of TEV residues 209, 211, 216, and 218 constructed with genetic template populations L1/L2 from stage 1 in Fig. 2~~A~~.

	Trajectory		S120	D127	S135	I138	D148	N176	R203
		A		D127A	S135F			N176I	
		B		D127A	S135F			N176I	
		C			S135F			N176I	
		D	S120N		S135F		D148A	N176I	
		E		D127A	S135F			N176I	
		F		D127A	S135F			N176I	
		G		D127A	S135F		D148A	N176I	R203Q
L1		H	S120N		S135F		D148A	N176I	
		A				I138T		N176T	
		B				I138T	D148A	N176T	
		C				I138T	D148A	N176T	
		D				I138T	D148A	N176T	
		E				I138T	D148A	N176T	
		F				I138T	D148A	N176T	
		G				I138T	D148A	N176T	
L2	3	H				I138T	D148A	N176T	

Supplementary Table 5. Clonal sequencing data from trajectory 3 after PACE stage 2 in Fig. 2~~A~~ (168 cumulative hours of evolution). Lagoons 1 and 2 (L1 and L2) correspond to two separate biological replicate populations that were inoculated from L1 and L2 respectively from PACE stage 1 (see Supplementary Table 3). Samples from these populations were combined into a single inoculum for L3 during PACE stage 3.

	Trajectory		G32	V63	E106	E107	D127	F132	S135	T146	D148	H167	N171	N176	N177	S200	V209	W211	V216	M218	E223	Q226	K229
L1	1	A					D127A		S135F	T146A	D148P			N176I	N177M		V209M	W211I		M218F			
		B			E106G		D127A		S135F	T146A	D148P			N176I	N177R		V209M	W211I		M218F			
		C					D127A		S135F	T146A	D148P			N176I	N177R		V209M	W211I		M218F			
		D					D127A		S135F	T146R	D148C	H167P		N176I	N177G		V209M	W211I		M218F			
		E					D127A		S135F	T146A	D148P			N176I	N177W		V209M	W211I		M218F			
		F					D127A		S135F	T146C	D148P			N176I	N177M		V209M	W211I		M218F			
		G					D127A		S135F	T146C	D148P				N177M	S200G	V209M	W211I		M218F			
		H					D127A		S135F	T146C	D148P			N176I	N177M		V209M	W211I		M218F			
L2	2	A					D127A		S135F	T146S	D148P		N171D	N176T	N177M		V209M	W211I		M218F			K229E
		B					D127A		S135F	T146C	D148P		N171D	N176T	N177M		V209E	W211L	V216I	M218W	E223stop		
		C					D127A		S135F	T146S	D148P		N171D	N176T	N177M		V209M	W211I		M218F			K229E
		D					D127A		S135F	T146C	D148P		N171D	N176T	N177M		V209E	W211L	V216I	M218W			
		E		V63I			D127A	F132S	S135F	T146S	D148P		N171D	N176T	N177M		V209M	W211I		M218F			K229E
		F					D127A		S135F	T146S	D148P		N171D	N176T	N177M		V209M	W211I		M218F			K229E
		G					D127A		S135F	T146C	D148P		N171D	N176T	N177M		V209E	W211L	V216I	M218W	E223stop		
		H					D127A		S135F	T146C	D148P		N171D	N176T	N177M		V209M	W211I		M218F			K229E
L3	3	C				E107D	D127A		S135F	T146A	D148A			N176I			V209S	W211I		M218W			
		D				E107D	D127A		S135F		D148A			N176I			V209E	W211L	V216I	M218W		Q226stop	
		E				E107D	D127A		S135F		D148A			N176I			V209S	W211I		M218W			
		F				E107D	D127A		S135F	T146A	D148A			N176I			V209E	W211L	V216I	M218W			
		G	G32R			E107D	D127A		S135F	T146A	D148A			N176I			V209E	W211L	V216I	M218W		Q226stop	
		H				E107D	D127A		S135F		D148A			N176I			V209F	W211C		M218L			

Supplementary Table 6. Clonal sequencing data from trajectory 1, 2, and 3 after PACE stage 3 in [illegible]. 2A (264 cumulative hours of evolution).

	Trajectory		K6	P8	R9	N12	T17	T30	R50	K67	N68	R80	E106	E107	T118	D127	S135	T146	D148	S152	R159	F162	N171	N176	N177	V209	W211	M218	Q226	P227	K229	Q233
		A														D127A	S135F	T146A	D148P					N176I	N177W	V209M	W211I	M218F			K229E	Q233stop
		B														D127A	S135F	T146A	D148P					N176I	N177W	V209M	W211I	M218F			K229E	
		C		P8L												D127A	S135F	T146A	D148P					N176I	N177W	V209M	W211I	M218F			K229E	
		D														D127A	S135F	T146A	D148P					N176I	N177W	V209M	W211I	M218F			K229E	Q233stop
		E														D127A	S135F	T146A	D148P					N176I	N177W	V209M	W211I	M218F			K229E	Q233stop
		F														D127A	S135F	T146A	D148P					N176I	N177W	V209M	W211I	M218F			K229E	Q233stop
		G														D127A	S135F	T146A	D148P		R159K			N176I	N177R	V209M	W211I	M218F			K229E	
L1	1a	H														D127A	S135F	T146A	D148P					N176I	N177R	V209M	W211I	M218F			K229E	
		A														D127A	S135F	T146S	D148P			F162S	N171D	N176T	N177M	V209M	W211I	M218F			K229E	
		B														D127A	S135F	T146S	D148P			F162S	N171D	N176T	N177M	V209M	W211I	M218F			K229E	
		C														D127A	S135F	T146S	D148P			F162S	N171D	N176T	N177M	V209M	W211I	M218F			K229E	
		D														D127A	S135F	T146S	D148P			F162S	N171D	N176T	N177M	V209M	W211I	M218F			K229E	
		E														D127A	S135F	T146S	D148P				N171D	N176T	N177M	V209M	W211I	M218F			K229E	
		F														D127A	S135F	T146S	D148P			F162S	N171D	N176T	N177M	V209M	W211I	M218F			K229E	
		G														D127A	S135F	T146S	D148P			F162S	N171D	N176T	N177M	V209M	W211I	M218F	Q226stop		K229E	
L2	2a	H														D127A	S135F	T146S	D148P			F162S	N171D	N176T	N177M	V209M	W211I	M218F			K229E	
		A				N12H								E107D		D127A	S135F	T146A	D148A					N176I		V209F	W211C	M218L	Q226stop			
		B												E107D		D127A	S135F	T146A	D148A					N176I		V209F	W211C	M218L		P227S		
		D												E107D		D127A	S135F	T146A	D148A					N176I		V209F	W211C	M218L				
		E								K67N				E107D		D127A	S135F	T146A	D148A	S152N				N176I		V209F	W211C	M218L				
		F												E107D		D127A	S135F	T146A	D148A					N176I		V209F	W211C	M218L				
		G												E107D		D127A	S135F	T146A	D148A					N176I		V209F	W211C	M218L				
L3	3a	H												E107D		D127A	S135F	T146A	D148A					N176I		V209F	W211C	M218L				
		A											E106G			D127A	S135F	T146S	D148P					N176I	N177F	V209M	W211I	M218F			K229E	
		B													T118S	D127A	S135F	T146A	D148P					N176I	N177R	V209M	W211I	M218F			K229E	
		C														D127A	S135F	T146A	D148P					N176I	N177R	V209M	W211I	M218F			K229E	
		D													T118S	D127A	S135F	T146A	D148P					N176I	N177R	V209M	W211I	M218F			K229E	
		E														D127A	S135F	T146A	D148P					N176I	N177R	V209M	W211I	M218F			K229E	
		F														D127A	S135F	T146A	D148P					N176I	N177R	V209M	W211I	M218F			K229E	
		G				N12T										D127A	S135F	T146A	D148P					N176I	N177R	V209M	W211I	M218F			K229E	
L4	1b	H										R80G				D127A	S135F	T146A	D148P					N176I	N177R	V209M	W211I	M218F			K229E	
		A														D127A	S135F	T146S	D148P				N171D	N176T	N177M	V209M	W211I	M218F			K229E	
		B														D127A	S135F	T146S	D148P				N171D	N176T	N177M	V209M	W211I	M218F			K229E	
		D														D127A	S135F	T146S	D148P				N171D	N176T	N177M	V209M	W211I	M218F			K229E	
		E	K6E													D127A	S135F	T146S	D148P				N171D	N176T	N177M	V209M	W211I	M218F			K229E	
		F					T17A									D127A	S135F	T146S	D148P				N171D	N176T	N177M	V209M	W211I	M218F			K229E	
L5	2b	G									N68D					D127A	S135F	T146S	D148P				N171D	N176T	N177M	V209M	W211I	M218F			K229E	
		A											E106G	E107D		D127A	S135F	T146A	D148A					N176I		V209F	W211C	M218L				
		B												E107D		D127A	S135F	T146A	D148A					N176I				M218F	Q226stop			
		C						T30A	R50K					E107D		D127A	S135F	T146A	D148A					N176I		V209F	W211C	M218L				
		D												E107D		D127A	S135F	T146A	D148A					N176I				M218F	Q226stop			
		E												E107D		D127A	S135F	T146A	D148A					N176I				M218F	Q226stop			
		F												E107D		D127A	S135F	T146A	D148A					N176I				M218F	Q226stop			
		G			R9C									E107D		D127A	S135F	T146A	D148A					N176I		V209F	W211C	M218L				
L6	3b	H											E106G	E107D		D127A	S135F	T146A	D148A					N176I								

Supplementary Table 7. Clonal sequencing data from trajectory 1, 2, and 3 after PACE stage 4 in Fig. 2~~A~~ (336 cumulative hours of evolution). Lagoons 1-3 underwent selection on substrate HNLVGHS and were inoculated with samples from lagoons 1-3 respectively from PACE stage 3. Lagoons 4-6 underwent selection on substrate HPLVGHM and were inoculated with samples from lagoons 1-3 respectively from PACE stage 3.

	Trajectory		E2	K6	H28	E106	E107	D127	S135	T146	D148	S153	F162	S170	N171	N176	N177	K184	R203	V209	W211	M218	Q226	P227	V228	K229
		A	E2K					D127A	S135F	T146C	D148P			S170A		N176I	N177S		R203Q	V209M	W211I	M218F	Q226stop			
		B				E106G		D127A	S135F	T146A	D148P	S153N		S170A		N176I	N177R	K184T		V209M	W211I	M218F				K229E
		C	E2K					D127A	S135F	T146C	D148P			S170A		N176I	N177S		R203Q	V209M	W211I	M218F	Q226stop			
		D	E2K					D127A	S135F	T146C	D148P			S170A		N176I	N177S		R203Q	V209M	W211I	M218F	Q226stop			
		E	E2K					D127A	S135F	T146C	D148P			S170A		N176I	N177S		R203Q	V209M	W211I	M218F	Q226stop			
		F	E2K					D127A	S135F	T146C	D148P			S170A		N176I	N177S		R203Q	V209M	W211I	M218F	Q226stop			
		G	E2K					D127A	S135F	T146C	D148P			S170A		N176I	N177S		R203Q	V209M	W211I	M218F	Q226stop			
L1	1	H						D127A	S135F	T146A	D148P	S153N		S170A		N176I	N177R			V209M	W211I	M218F				K229E
		A						D127A	S135F	T146S	D148P		F162S		N171D	N176T	N177M			V209M	W211I	M218F				K229E
		B			H28Y			D127A	S135F	T146S	D148P		F162S		N171D	N176T	N177M			V209M	W211I	M218F				K229E
		C			H28L			D127A	S135F	T146S	D148P		F162S		N171D	N176T	N177M			V209M	W211I	M218F				K229E
		D		K6E				D127A	S135F	T146S	D148P		F162S		N171D	N176T	N177M			V209M	W211I	M218F				K229E
		E						D127A	S135F	T146S	D148P		F162S	S170A	N171D	N176T	N177M			V209M	W211I	M218F				K229E
		F			H28L			D127A	S135F	T146S	D148P		F162S		N171D	N176T	N177M			V209M	W211I	M218F				K229E
		G		K6E				D127A	S135F	T146S	D148P		F162S		N171D	N176T	N177M			V209M	W211I	M218F				K229E
L2	2	H						D127A	S135F	T146S	D148P		F162S	S170A	N171D	N176T	N177M			V209M	W211I	M218F				K229E
		A					E107D	D127A	S135F	T146A	D148A					N176I				V209F	W211C	M218L	Q226S	P227A	V228S	K229stop
		B			H28Y		E107D	D127A	S135F	T146A	D148A	S153N				N176I				V209F	W211C	M218L	Q226S	P227A	V228S	K229stop
		D			H28Y		E107D	D127A	S135F	T146A	D148A	S153N				N176I				V209F	W211C	M218L	Q226S	P227A	V228S	K229stop
		E			H28Y		E107D	D127A	S135F	T146A	D148A	S153N				N176I				V209F	W211C	M218L	Q226S	P227A	V228S	K229stop
		F					E107D	D127A	S135F	T146A	D148A					N176I				V209F	W211C	M218L	Q226S	P227A	V228S	K229stop
L3	3	H			H28Y		E107D	D127A	S135F	T146A	D148A					N176I				V209F	W211C	M218L	Q226S	P227A	V228S	K229stop

Supplementary Table 8. Clonal sequencing data from trajectory 1, 2, and 3 after PACE stage 5 i[illegible]ig. 2~~A~~ (456 cumulative hours of evolution). The six isolated populations resulting from PACE stage four were recombined into three in a manner that maintains isolation of populations that originated from the three original lagoons during stage three. More specifically, lagoon 1 was inoculated with a 1:1 volumetric mixture of lagoons 1 and 4. Lagoon 2 was inoculated with a 1:1 volumetric mixture of lagoons 2 and 5. Lagoon 3 was inoculated with a 1:1 volumetric mixture of lagoons 3 and 6.

	Trajectory		E2	F5	K6	D10	P13	T17	H28	T30	P39	F40	R50	H61	N68	K89	K99	E106	E107	I109	T114	M121	M124	D127	C130	F132	S135	T146	D148	S153	F162	S170	N171	N176	N177	R203	V209	W211	M218	K220	E223	Q226	P227	V228	K229	A231	L234
		A	E2K																					D127A			S135F	T146C	D148P			S170A		N176I	N177S	R203Q	V209M	W211I	M218F			Q226stop					
		B	E2K								P39S													D127A			S135F	T146C	D148P		F162S	S170A		N176I	N177S	R203Q	V209M	W211I	M218F			Q226stop					
		C	E2K																E107D					D127A			S135F	T146C	D148P			S170A		N176I	N177S	R203Q	V209M	W211I	M218F			Q226stop					
		D	E2K																					D127A			S135F	T146C	D148P			S170A		N176I	N177S	R203Q	V209M	W211I	M218F			Q226stop					
		E	E2K						H28Y						N68D									D127A			S135F	T146C	D148P			S170A		N176I	N177S	R203Q	V209M	W211I	M218F			Q226stop					
		F	E2K																					D127A			S135F	T146C	D148P			S170A		N176I	N177S	R203Q	V209M	W211I	M218F			Q226stop					
		G	E2K																					D127A			S135F	T146C	D148P			S170A		N176I	N177S	R203Q	V209M	W211I	M218F		E223G	Q226stop					
L1	1a	H	E2K																					D127A			S135F	T146C	D148P			S170A		N176I	N177S	R203Q	V209M	W211I	M218F			Q226stop					
		A																	E107D		T114A			D127A			S135F	T146S	D148P		F162S	S170A	N171D	N176T	N177M		V209M	W211I	M218F						K229E		
		B							H28L	T30A								E106D						D127A			S135F	T146S	D148P		F162S	S170A	N171D	N176T	N177M		V209M	W211I	M218F						K229E		
		D			K6E				H28L															D127A			S135F	T146S	D148P		F162S		N171D	N176T	N177M		V209M	W211I	M218F		E223stop						
		E			K6E																			D127A			S135F	T146S	D148P		F162S		N171D	N176T	N177M		V209M	W211I	M218F						K229E		
		F			K6E																			D127A			S135F	T146S	D148P		F162S	S170A	N171D	N176T	N177M		V209M	W211I	M218F						K229E		
		G			K6E					T30A														D127A			S135F	T146S	D148P		F162S	S170A	N171D	N176T	N177M		V209M	W211I	M218F						K229E		
L2	2a	H			K6E																			D127A			S135F	T146S	D148P		F162S		N171D	N176T	N177M		V209M	W211I	M218F						K229E		
		B							H28Y										E107D					D127A			S135F	T146A	D148A	S153N				N176I			V209F	W211C	M218L			Q226S	P227A	V228S	K229stop		
		C							H28Y							K89E			E107D			M121T		D127A			S135F	T146A	D148A	S153N				N176I			V209F	W211C	M218L			Q226S	P227A	V228S	K229stop		
		D							H28Y										E107D					D127A			S135F	T146A	D148A	S153N				N176I			V209F	W211C	M218L			Q226S	P227A	V228S	K229stop		
		E							H28Y			F40L							E107D					D127A			S135F	T146A	D148A	S153N				N176I			V209F	W211C	M218L			Q226S	P227A	V228S	K229stop		
		G							H28Y										E107D					D127A			S135F	T146A	D148A	S153N				N176I			V209F	W211C	M218L			Q226S	P227A	V228S	K229stop		
L3	3a	H							H28Y										E107D	I109V				D127A			S135F	T146A	D148A	S153N			N171K	N176I			V209F	W211C	M218L			Q226S	P227A	V228S	K229stop		
		A	E2K			D10N																		D127A			S135F	T146C	D148P			S170A		N176I	N177S	R203Q	V209M	W211I	M218F			Q226stop					
		B	E2K																					D127A			S135F	T146C	D148P			S170A		N176I	N177S	R203Q	V209M	W211I	M218F			Q226stop					
		D	E2K																					D127A			S135F	T146C	D148P			S170A		N176I	N177S	R203Q	V209M	W211I	M218F			Q226stop					
		E															K99Q	E106G					M124I	D127A			S135F	T146A	D148P	S153N		S170A		N176I	N177R		V209M	W211I	M218F						K229E		
		F	E2K																					D127A			S135F	T146C	D148P			S170A		N176I	N177S	R203Q	V209M	W211I	M218F			Q226stop					
		G	E2K											H61R										D127A			S135F	T146C	D148P			S170A		N176I	N177S	R203Q	V209M	W211I	M218F			Q226stop					
L4	1b																																													
		A							H28L												T114A			D127A			S135F	T146S	D148P	S153N	F162S	S170A	N171D	N176T	N177M		V209M	W211I	M218F						K229E		
		B		F5L				T17A					R50G						E107D					D127A			S135F	T146S	D148P		F162S	S170A	N171D	N176T	N177M		V209M	W211I	M218F						K229E		
		C											R50G											D127A	C130R		S135F	T146S	D148P		F162S	S170A	N171D	N176T	N177M		V209M	W211I	M218F	K220Q					K229E		
		D											R50G							I109V				D127A			S135F	T146S	D148P		F162S	S170A	N171D	N176T	N177M		V209M	W211I	M218F		E223stop				K229E		
		E					P13S						R50G											D127A		F132V	S135F	T146S	D148P		F162S	S170A	N171D	N176T	N177M		V209M	W211I	M218F						K229E		L234R
		F											R50G							I109V				D127A			S135F	T146S	D148P		F162S	S170A	N171D	N176T	N177M		V209M	W211I	M218F						K229E		
		G																						D127A			S135F	T146S	D148P		F162S	S170A	N171D	N176T	N177M		V209M	W211I	M218F						K229E	A231E	
L5	2b																																													
		A							H28Y										E107D					D127A			S135F	T146A	D148A	S153N				N176I			V209F	W211C	M218L			Q226S	P227A	V228S	K229stop		
		B							H28Y						N68D				E107D					D127A			S135F	T146A	D148A	S153N				N176I			V209F	W211C	M218L			Q226S	P227A	V228S	K229stop		
		C							H28Y										E107D			M121T		D127A			S135F	T146A	D148A	S153N				N176I			V209F	W211C	M218L			Q226S	P227A	V228S	K229stop		
		D							H28Y										E107D					D127A			S135F	T146A	D148A	S153N				N176I			V209F	W211C	M218L			Q226S	P227A	V228S	K229stop		
		E							H28Y			F40L							E107D					D127A			S135F	T146A	D148A	S153N				N176I			V209F	W211C	M218L			Q226S	P227A	V228S	K229stop		
		F						T17A	H28Y										E107D					D127A			S135F	T146A	D148A	S153N				N176I			V209F	W211C	M218L			Q226S	P227A	V228S	K229stop		
		G							H28Y										E107D					D127A			S135F	T146A	D148A	S153N				N176I			V209F	W211C	M218L			Q226S	P227A	V228S	K229stop		
L6	3b	H							H28Y										E107D	I109V				D127A		F132V	S135F	T146A	D148A	S153N				N176I			V209F	W211C	M218L			Q226S	P227A	V228S	K229stop		

Supplementary Table 9. Clonal sequencing data from trajectory 1, 2, and 3 after PACE sta[illegible] in Fig. 2A (528 cumulative hours of evolution). Lagoons 1-3 underwent selection for cleavage of IL23 (38-66) and were inoculated with samples from lagoons 1-3 respectively from PACE stage 5. Lagoons 4-6 underwent selection on substrate HPLVGHM at increased stringency due to T7 RNAP mutation Q649S and were inoculated with samples from lagoons 1-3 respectively from PACE stage 5.

	Trajectory																																																	
		A	E2K																							D127A	S135F	T146C	D148P				S170A		N176I	N177S		R203Q	V209M	W211I		M218F		Q226stop						
		C	E2K																E107D							D127A	S135F	T146C	D148P				S170A		N176I	N177S		R203Q	V209M	W211I		M218F		Q226stop						
		D		T17A		E24K	H28L										K97N									D127P	S135F	T146A	D148P		S153N		S170A		N176I	N177R			V209M	W211I		M218F					K229E			
		E	E2K																							D127A	S135F	T146C	D148P				S170A		N176I	N177S		R203Q	V209M	W211I		M218F		Q226stop						
		G		T17A			H28L										K97N									D127P	S135F	T146A	D148P		S153N		S170A		N176I	N177R			V209M	W211I		M218F					K229E			
L1	1a	H		T17A			H28L										K97N									D127P	S135F	T146A	D148P		S153N		S170A		N176I	N177R			V209M	W211I		M218F					K229E			
		A						T30A																		D127A	S135F	T146S	D148P			F162A	S170A	N171D	N176T	N177M			V209M	W211V	K215E	M218F					K229E			
		B						T30A																		D127A	S135F	T146S	D148P			F162A	S170A	N171D	N176T	N177M			V209M	W211V	K215E	M218F					K229E			
		C						T30A																		D127A	S135F	T146S	D148P			F162A	S170A	N171D	N176T	N177M			V209M	W211V	K215E	M218F	F225L				K229E			
		D						T30A						T71P		K89E										D127A	S135F	T146S	D148P			F162A	S170A	N171D	N176T	N177M			V209M	W211V	K215E	M218F					K229E			
		E						T30A				T69P													M124T	D127A	S135F	T146S	D148P			F162A	S170A	N171D	N176T	N177M			V209M	W211I		M218F					K229E			
		F						T30A																		D127A	S135F	T146S	D148P			F162A	S170A	N171D	N176T	N177M			V209M	W211V	K215E	M218F					K229E			
L2	2a	H						T30A																		D127A	S135F	T146P	D148P	Q150P		F162A	S170A	N171D	N176T	N177M			V209M	W211V	K215E	M218F					K229E			
		A					H28Y												E107D							D127A	S135F	T146A	D148A		S153N				N176I				V209F	W211C		M218L								
		B					H28Y	T30A											E107D							D127A	S135F	T146A	D148A		S153N				N176I				V209F	W211C		M218L		Q226P	P227A	V228S	K229stop			
		C					H28Y	T30A											E107D							D127A	S135F	T146A	D148A		S153N				N176I				V209F	W211C		M218L		Q226P	P227A	V228S	K229stop			
		D					H28Y												E107D					S120N			D127A	S135F	T146A	D148A		S153N				N176I				V209F	W211C		M218L		Q226S	P227A	V228S	K229stop			
		E					H28Y	T30A											E107D								D127A	S135F	T146A	D148A		S153N				N176I				V209F	W211C		M218L								
		F					H28Y												E107D							D127A	S135F	T146A	D148A		S153N				N176I				V209F	W211C		M218L		Q226S	P227A	V228S	K229stop			
		G					H28Y												E107D							D127A	S135F	T146A	D148A		S153N				N176I				V209F	W211C		M218L		Q226S	P227A	V228S	K229stop			
L3	3a	H			I18V		H28Y												E107D							D127A	S135F	T146A	D148A						N176I				V209F	W211C		M218L		Q226P	P227A	V228S	K229stop			
		A	E2K																	I109V						D127A	S135F	T146C	D148P		S153N		S170A		N176I	N177S		R203Q	V209M	W211I		M218F		Q226stop						
		B	E2K															E106D								D127A	S135F	T146C	D148P		S153N		S170A		N176I	N177S	K184E	R203Q	V209M	W211I		M218F		Q226stop						
		C	E2K										T70P						E107D							D127A	S135F	T146C	D148P		S153N		S170A		N176I	N177S		R203Q	V209M	W211I		M218F		Q226stop						
		D	E2K					T30A										E106G								D127A	S135F	T146A	D148P		S153N		S170A		N176I	N177R			V209M	W211V		M218F					K229E		T232A	
		E	E2K																E107D		L111F				M124I	D127A	S135F	T146C	D148P		S153N		S170A		N176I	N177S		R203Q	V209M	W211I		M218F		Q226stop						
		F	E2K																E107D							D127A	S135F	T146C	D148P		S153N		S170A		N176I	N177S		R203Q	V209M	W211I		M218F		Q226stop						
L4	1b	G	E2K																E107D			T114A				D127A	S135F	T146C	D148P		S153N		S170A		N176I	N177S		R203Q	V209M	W211I		M218F		Q226stop						
		A		T17A			H28L																			D127A	S135F	T146S	D148P		S153N	F162S	S170A	N171D	N176T	N177M			V209M	W211I		M218F	F225S				K229E			
		B					H28L		H61Q																	D127A	S135F	T146S	D148P		S153N	F162S	S170A	N171D	N176T	N177M			V209M	W211I		M218F		Q226S	P227A	V228S	K229stop			
		C		T17A			H28L																			D127A	S135F	T146S	D148P		S153N	F162S	S170A	N171D	N176T	N177M			V209M	W211I		M218F		Q226S	P227A	V228S	K229stop			
		E		T17A			H28L																			D127A	S135F	T146S	D148P		S153N	F162S	S170A	N171D	N176T	N177M			V209M	W211I		M218F		Q226S	P227A	V228S	K229stop			L234R
		F		T17A			H28L				N68D															D127A	S135F	T146S	D148P		S153N	F162S	S170A	N171D	N176T	N177M			V209M	W211I		M218F		Q226S	P227A	V228S	K229stop			
		G	E2K	T17A			H28L																			D127A	S135F	T146S	D148P		S153N	F162S	S170A	N171D	N176T	N177M			V209M	W211I		M218F		Q226S	P227A	V228S	K229stop	A231E		
L5	2b	H					H28L				N68D															D127A	S135F	T146S	D148P		S153N	F162S	S170A	N171D	N176T	N177M			V209M	W211I		M218F		Q226S	P227stop					
		A					H28Y			V66G					H75L				E107D							D127A	S135F	T146A	D148A						N176I				V209F	W211C		M218L		Q226S	P227A	V228S	K229stop			
		C					H28Y												E107D					M121T		D127A	S135F	T146A	D148A						N176I				V209F	W211C		M218L		Q226S	P227A	V228S	K229stop			
		D					H28Y												E107D							D127A	S135F	T146A	D148A		S153N				N176I				V209F	W211C		M218L		Q226S	P227A	V228S	K229stop			
		E					H28Y												E107D							D127A	S135F	T146A	D148A		S153N				N176I				V209F	W211C		M218L		Q226S	P227A	V228S	K229stop			
		F					H28Y												E107D							D127A	S135F	T146A	D148A		S153N				N176I				V209F	W211C		M218L		Q226S	P227A	V228S	K229stop			
L6	3b	G					H28Y												E107D							D127A	S135F	T146A	D148A		S153N				N176I				V209F	W211C		M218L		Q226S	P227A	V228S	K229stop			

Supplementary Table 10. Clonal sequencing data from trajectory 1, 2, and 3 after PACE stage 7 in Fig. 2A (600 cumulative hours of evolution).

	Trajectory		E2	F5	R9	D10	N12	T17	E24	D26	H28	T30	K67	N68	Q73	R80	Q104	E107	T118	V125	D127	T128	C130	F132	S135	Q145	T146	D148	S153	F162	S170	N171	N176	N177	V182	N185	Q193	R203	V209	W211	K215	M218	P221	E222	Q226	P227	V228	K229
		A	E2K															E107D			D127A				S135F		T146C	D148P			S170A		N176I	N177S				R203Q	V209M	W211I	K215E	M218F			Q226stop			
		B	E2K															E107D			D127A				S135F		T146C	D148P			S170A		N176I	N177S				R203Q	V209M	W211I	K215E	M218F			Q226stop			
		C	E2K															E107D			D127A				S135F		T146C	D148P			S170A		N176I	N177S				R203Q	V209M	W211I	K215E	M218F			Q226stop			
		D	E2K															E107D			D127A				S135F		T146C	D148P			S170A		N176I	N177S				R203Q	V209M	W211I	K215E	M218F			Q226stop			
		E	E2K															E107D			D127A				S135F		T146C	D148P			S170A		N176I	N177S				R203Q	V209M	W211I	K215E	M218F			Q226stop			
		F	E2K															E107D			D127A				S135F		T146C	D148P	S153N		S170A		N176I	N177S				R203Q	V209M	W211I	K215E	M218F			Q226stop			
		G	E2K															E107D			D127A				S135F		T146C	D148P			S170A		N176I	N177S				R203Q	V209M	W211I	K215E	M218F			Q226stop			
L1	1a	H	E2K															E107D			D127A				S135F		T146C	D148P			S170A		N176I	N177S				R203Q	V209M	W211I	K215E	M218F			Q226stop			
		B									H28L							E107D			D127A	T128P			S135F		T146S	D148P	S153N	F162S	S170A	N171D	N176T	N177M					V209M	W211I		M218F						K229E
		C			R9C						H28L	T30A		N68D				E107D			D127A			F132L	S135F		T146S	D148P	S153N	F162S	S170A	N171D	N176T	N177M	V182G				V209M	W211I		M218F						K229E
		F						T17S			H28L	T30A		N68D				E107D			D127A			F132L	S135F		T146S	D148P	S153N	F162S	S170A	N171D	N176T	N177M					V209M	W211I		M218F						K229E
L2	2a	H									H28L	T30A		N68D				E107D			D127A			F132L	S135F		T146S	D148P	S153N	F162S	S170A	N171D	N176T	N177M					V209M	W211I		M218F						K229E
		A									H28Y						Q104R	E107D			D127A			F132S	S135F		T146A	D148A	S153N				N176I						V209F	W211C		M218L			Q226S	P227A	V228S	K229stop
		B									H28Y							E107D			D127A				S135F		T146A	D148A	S153N				N176I						V209F	W211C		M218L			Q226S	P227A	V228S	K229stop
		C							E24V		H28Y							E107D			D127A				S135F		T146A	D148A	S153N				N176I						V209F	W211C		M218L			Q226S	P227A	V228S	K229stop
		D									H28Y							E107D			D127A				S135F		T146A	D148A	S153N				N176I						V209F	W211C		M218L			Q226S	P227A	V228S	K229stop
		E	E2G								H28Y							E107D			D127A				S135F		T146A	D148A					N176I			N185D			V209F	W211C		M218L			Q226S	P227A	V228S	K229stop
		F									H28Y			N68D				E107D			D127A				S135F		T146A	D148A					N176I						V209F	W211C		M218L			Q226S	P227A	V228S	K229stop
		G							E24K		H28Y			N68D				E107D			D127A	T128A			S135F		T146A	D148A					N176I						V209F	W211C		M218L			Q226S	P227A	V228S	K229stop
L3	3a	H									H28Y							E107D			D127A				S135F		T146A	D148A	S153N				N176I						V209F	W211C		M218L			Q226S	P227A	V228S	K229stop
		A	E2K							D26Y								E107D			D127A				S135F		T146C	D148P			S170A		N176I	N177S				R203Q	V209M	W211I		M218L			Q226stop			
		B	E2K															E107D			D127A				S135F		T146C	D148P			S170A		N176I	N177S				R203Q	V209M	W211I		M218F			Q226stop			
		C	E2K															E107D			D127A				S135F		T146C	D148P			S170A		N176I	N177S				R203Q	V209M	W211I		M218F			Q226stop			
		D	E2K															E107D			D127A				S135F		T146C	D148P			S170A		N176I	N177S				R203Q	V209M	W211I		M218F			Q226stop			
		E	E2K													R80G					D127A				S135F		T146C	D148P			S170A		N176I	N177S				R203Q	V209M	W211I	K215E	M218F			Q226stop			
		F	E2K					T17A										E107D			D127A				S135F		T146C	D148P			S170A		N176I	N177S				R203Q	V209M	W211I		M218F			Q226stop			
		G	E2K															E107D			D127A				S135F		T146C	D148P			S170A		N176I	N177S				R203Q	V209M	W211I		M218F			Q226stop			
L4	1b	H	E2K															E107D			D127A				S135F		T146C	D148P			S170A		N176I	N177S				R203Q	V209M	W211I		M218F			Q226stop			
		A		F5L								T30A			Q73H				T118A		D127A		C130R		S135F		T146S	D148P		F162A	S170A	N171D	N176T	N177M					V209M	W211V	K215E	M218F	P221T	E222stop				
		B										T30A									D127A				S135F		T146S	D148P		F162A	S170A	N171D	N176T	N177M					V209M	W211V	K215E	M218F	P221T	E222stop				
		C										T30A								V125G	D127A				S135F		T146S	D148P		F162A	S170A	N171D	N176T	N177M					V209M	W211V	K215E	M218F	P221T	E222stop				
		D										T30A									D127A				S135F		T146S	D148P		F162A	S170A	N171D	N176T	N177M					V209M	W211V	K215E	M218F	P221T	E222stop				
		E										T30A									D127A				S135F		T146S	D148P		F162A	S170A	N171D	N176T	N177M					V209M	W211V	K215E	M218F						K229E
		F										T30A									D127A				S135F		T146S	D148P		F162A	S170A	N171D	N176T	N177M					V209M	W211V	K215E	M218F	P221T	E222stop				
		G										T30A									D127A				S135F		T146S	D148P		F162A	S170A	N171D	N176T	N177M					V209M	W211V	K215E	M218F	P221T	E222stop				
L5	2b	H										T30A			Q73H				T118A		D127A		C130R		S135F	Q145P	T146S	D148P		F162A	S170A	N171D	N176T	N177M					V209M	W211V	K215E	M218F	P221T	E222stop				
		A					N12T				H28Y							E107D			D127A				S135F		T146A	D148A	S153N				N176I						V209F	W211C		M218L			Q226S	P227A	V228S	K229stop
		B									H28Y							E107D			D127A				S135F		T146A	D148A	S153N				N176I						V209F	W211C		M218L			Q226S	P227A	V228S	K229stop
		C				D10G					H28Y		K67E					E107D			D127A				S135F		T146A	D148A	S153N				N176I				Q193P		V209F	W211C		M218L			Q226S	P227A	V228S	K229stop
		D									H28Y							E107D			D127A				S135F		T146A	D148A	S153N				N176I						V209F	W211C		M218L			Q226S	P227A	V228S	K229stop
		E									H28Y							E107D			D127A				S135F		T146A	D148A	S153N				N176I						V209F	W211C		M218L			Q226S	P227A	V228S	K229stop
		F									H28Y							E107D			D127A				S135F		T146A	D148A	S153N				N176I						V209F	W211C		M218L			Q226S	P227A	V228S	K229stop
L6	3b	G									H28Y							E107D			D127A				S135F		T146A	D148A	S153N				N176I						V209F	W211C		M218L						

Supplementary Table 11. Clonal sequencing data from trajectory 1, 2, and 3 after PACE stage 8 in Fig. 2A (672 cumulative hours of evolution).

Wild-type TEV

	A	C	D	E	F	G	H	I	K	L	M	N	P	Q	R	S	T	V	W	Y
P6	-0.19	0.08	1.03	5.69	0.99	-0.16	0.03	0.41	-0.20	0.42	0.31	-0.10	0.04	0.23	-0.14	-0.05	-0.01	0.26	0.37	-0.01
P5	0.30	-0.25	0.45	-0.07	0.25	-0.46	0.15	-0.19	-0.06	0.21	0.10	-0.51	-0.24	0.02	0.02	0.10	-0.04	0.20	0.14	0.24
P4	-0.34	-0.29	-0.31	-0.15	-0.33	-0.31	-0.40	5.16	-0.29	3.16	0.68	-0.32	-0.37	-0.39	-0.33	-0.35	-0.31	0.39	-0.21	-0.39
P3	-0.50	-0.52	-0.09	-0.54	0.84	0.30	-0.48	-0.11	-0.50	-0.37	-0.54	-0.45	-0.39	-0.52	-0.29	-0.56	-0.58	0.01	-0.26	4.08
P2	-0.19	-0.74	-0.48	-0.66	3.81	-0.39	-0.70	3.79	-0.69	0.48	0.85	-0.59	-0.03	-0.72	-0.62	-0.38	0.19	4.36	0.92	-0.72
P1	-0.22	-0.06	-0.14	-0.12	-0.16	-0.15	-0.08	-0.24	-0.24	-0.18	1.29	-0.02	-0.22	5.49	-0.27	-0.24	-0.17	-0.28	-0.16	-0.26
P1'	2.05	-0.50	-0.03	-0.48	1.60	1.96	1.22	-0.39	-0.58	-0.44	1.38	0.07	-0.68	-0.33	-0.62	2.77	-0.33	-0.60	2.24	2.60

TEV L2F

T17S, H28L, T30A, N68D, E107D, D127A, F132L, S135F, T146S, D148P, S153N, F162S, S170A, N171D, N176T, N177M, V209M, W211I, M218F, K229E

	A	C	D	E	F	G	H	I	K	L	M	N	P	Q	R	S	T	V	W	Y
P6	0.50	-0.59	-0.04	0.02	-0.08	0.01	-0.01	0.11	0.02	0.02	0.07	-0.03	-0.03	-0.02	0.01	-0.07	-0.01	0.02	0.15	-0.72
P5	0.01	-0.57	-0.08	0.03	-0.05	0.00	0.09	-0.05	0.01	0.01	0.06	-0.23	0.07	0.03	0.09	-0.01	-0.03	0.00	-0.02	0.06
P4	0.03	-0.40	-0.21	-0.12	-0.11	-0.32	-0.41	3.80	-0.25	4.24	1.30	-0.16	-0.31	-0.38	-0.35	-0.32	-0.33	1.23	-0.20	-0.37
P3	-0.66	-0.86	-0.58	-0.79	1.42	-0.51	-0.13	1.82	-0.65	1.12	1.30	-0.78	-0.88	0.02	0.00	-0.85	-0.36	1.73	1.43	1.36
P2	0.50	-0.85	-0.83	-0.79	3.04	-0.64	-0.81	1.76	-0.82	0.53	-0.18	-0.70	-0.56	-0.80	-0.73	-0.28	0.02	1.94	2.67	2.22
P1	0.45	-0.66	0.11	1.74	1.97	-0.08	2.35	-0.70	-0.62	1.89	2.60	0.68	-0.54	3.04	-0.54	0.12	1.33	-0.76	-0.15	2.71
P1'	3.22	-0.51	0.98	1.36	2.07	2.13	1.99	3.16	-0.77	2.06	3.01	-0.80	-0.80	1.50	-0.74	2.45	2.26	2.69	2.52	2.87

TEV I138T, N171D, N176T

	A	C	D	E	F	G	H	I	K	L	M	N	P	Q	R	S	T	V	W	Y
P6	-0.81	-0.50	0.59	0.78	1.41	-0.51	-0.12	1.53	1.45	1.60	1.70	-0.42	1.50	1.06	-0.08	0.28	0.48	1.46	0.42	0.19
P5	0.63	-0.19	1.66	1.25	2.49	-0.76	0.85	2.32	-0.57	2.11	2.16	-0.06	2.39	0.93	-0.48	0.07	0.72	1.97	3.04	2.81
P4	-0.20	-0.22	-0.41	-0.25	-0.12	-0.16	-0.24	1.69	-0.13	4.15	-0.01	-0.13	-0.04	-0.14	-0.18	-0.21	-0.28	-0.25	-0.09	-0.27
P3	-0.26	-0.35	0.84	0.26	-0.08	1.62	-0.12	-0.33	-0.19	-0.25	-0.39	0.00	-0.19	-0.19	-0.12	-0.23	-0.30	-0.26	-0.26	3.35
P2	-0.06	-0.40	-0.10	-0.30	5.21	-0.18	-0.34	0.05	-0.29	-0.21	-0.38	-0.33	-0.77	-0.31	-0.18	-0.21	-0.26	1.74	-0.27	-0.48
P1	-0.10	-0.12	-0.47	-0.12	-0.13	-0.01	-0.20	-0.25	-0.06	-0.17	-0.11	-0.18	0.08	4.05	-0.08	-0.05	-0.07	-0.26	-0.11	-0.24
P1'	1.24	-0.31	-0.44	-0.29	0.20	0.74	0.05	-0.25	-0.33	-0.27	-0.04	-0.37	-0.36	-0.43	-0.40	2.35	-0.41	-0.41	1.24	-0.06

TEV T146S, D148P, S153N, S170A, N177M

	A	C	D	E	F	G	H	I	K	L	M	N	P	Q	R	S	T	V	W	Y
P6	0.48	0.35	1.31	2.68	0.52	-0.03	0.09	0.31	-0.16	0.26	0.36	0.18	0.15	0.34	-0.14	-0.01	0.10	0.21	0.18	0.19
P5	0.30	-0.48	0.20	0.30	0.28	-0.55	-0.06	0.42	-0.25	0.27	0.40	-0.23	0.30	0.30	-0.04	-0.08	0.17	0.32	0.30	0.17
P4	-0.34	-0.34	-0.39	-0.08	-0.35	-0.27	-0.37	4.37	-0.22	4.49	0.91	-0.20	-0.28	-0.24	-0.29	-0.33	-0.32	0.50	-0.31	-0.47
P3	-0.67	-0.73	-0.48	-0.59	0.83	0.02	-0.49	0.86	-0.72	-0.15	-0.38	-0.56	-0.74	-0.56	-0.45	-0.74	-0.69	0.81	0.66	2.27
P2	-0.36	-0.64	-0.58	-0.49	3.55	-0.47	-0.51	1.16	-0.65	0.14	-0.01	-0.42	-0.27	-0.56	-0.49	-0.37	-0.23	0.39	1.18	-0.43
P1	0.15	-0.43	0.14	1.21	1.55	-0.14	2.02	-0.78	-0.59	1.57	2.23	0.14	-0.61	2.16	-0.56	0.12	0.86	-0.78	0.08	1.99
P1'	1.00	-0.32	0.49	0.38	1.11	0.81	0.54	0.90	-0.60	0.33	0.73	-0.76	-0.81	0.13	-0.56	1.92	0.32	-0.08	1.00	2.71

TEV V209M, W211I, M218F

	A	C	D	E	F	G	H	I	K	L	M	N	P	Q	R	S	T	V	W	Y
P6	0.91	0.31	0.39	2.61	0.19	-0.01	0.01	0.22	0.31	0.29	0.28	0.09	0.11	0.17	-0.12	0.05	0.10	0.18	0.05	-0.46
P5	1.06	-0.37	1.42	1.05	1.65	-0.78	1.14	2.04	-0.55	1.47	1.45	0.36	1.88	0.60	-0.45	0.19	0.84	1.90	1.78	1.94
P4	-0.31	-0.22	-0.07	-0.13	-0.23	-0.19	-0.30	2.68	-0.13	4.45	0.19	-0.11	-0.17	-0.18	-0.23	-0.27	-0.27	0.26	-0.19	-0.26
P3	-0.39	-0.46	-0.03	-0.30	0.32	0.72	-0.34	-0.10	-0.34	-0.17	-0.44	-0.11	-0.23	-0.32	-0.03	-0.45	-0.44	-0.09	-0.16	3.38
P2	0.06	-0.50	-0.42	-0.49	3.77	-0.28	-0.50	-0.26	-0.51	-0.37	-0.27	-0.27	0.41	-0.56	-0.38	-0.29	-0.21	0.05	1.41	0.49
P1	-0.03	-0.07	0.06	0.20	-0.22	0.07	0.04	-0.30	-0.08	-0.25	0.51	-0.01	-0.07	3.92	-0.12	-0.07	-0.02	-0.29	-0.23	-0.29
P1'	0.79	-0.13	-0.07	-0.16	0.39	1.08	0.56	-0.33	-0.39	-0.36	-0.08	-0.50	-0.46	-0.30	-0.44	2.44	-0.24	-0.46	0.88	0.55

TEV H28L, T30A

	A	C	D	E	F	G	H	I	K	L	M	N	P	Q	R	S	T	V	W	Y
P6	0.56	0.18	0.24	0.33	0.58	0.04	-0.03	0.00	0.17	0.07	0.04	0.13	0.00	-0.05	-0.04	0.11	-0.01	-0.10	0.25	0.09
P5	0.11	-0.05	0.46	0.43	0.32	-0.39	-0.14	0.22	-0.45	0.32	0.66	-0.16	1.07	0.00	-0.38	-0.28	0.08	0.34	1.05	0.29
P4	-0.05	-0.03	-0.12	0.04	-0.11	0.03	-0.07	0.26	0.08	0.60	-0.12	0.04	0.06	-0.05	0.01	-0.01	-0.14	-0.10	-0.09	-0.20
P3	0.08	-0.07	-0.02	0.29	0.00	0.45	0.11	-0.16	0.23	-0.09	-0.20	0.22	0.11	0.03	0.04	0.03	-0.04	-0.20	-0.10	0.51
P2	0.07	-0.14	-0.13	-0.02	0.74	-0.08	-0.10	0.54	0.02	-0.09	-0.18	0.03	-0.35	-0.10	0.08	0.00	0.04	0.15	-0.18	-0.26
P1	-0.10	-0.09	0.16	0.01	-0.09	0.18	-0.07	-0.12	0.08	-0.16	-0.05	0.01	0.08	0.51	0.05	-0.02	-0.03	-0.15	-0.07	-0.14
P1'	0.02	-0.20	-0.21	-0.08	0.21	0.16	-0.04	-0.02	-0.18	-0.11	0.24	-0.21	-0.19	-0.22	-0.26	0.33	-0.07	-0.11	0.97	0.06

TEV T17S, N68D, E107D, D127A, F132L, S135F, F162S, K229E

	A	C	D	E	F	G	H	I	K	L	M	N	P	Q	R	S	T	V	W	Y
P6	-0.85	-0.16	0.45	2.26	0.47	-0.06	-0.12	0.23	3.24	0.43	0.48	-0.09	-0.05	0.53	-0.08	-0.05	-0.02	0.16	-0.08	-0.05
P5	0.40	-0.42	0.10	0.90	0.92	-0.65	-0.31	1.30	-0.49	1.11	1.07	-0.44	1.43	0.59	-0.15	-0.30	0.53	1.13	1.47	0.92
P4	-0.21	-0.16	1.06	-0.06	-0.36	-0.06	-0.18	2.69	-0.12	2.92	0.00	-0.04	-0.10	-0.14	-0.12	-0.19	-0.21	0.00	-0.20	-0.36
P3	-0.27	-0.37	-0.34	-0.17	0.20	0.74	-0.23	-0.25	0.25	-0.26	-0.37	-0.08	0.06	-0.25	-0.09	-0.27	-0.35	-0.16	-0.15	2.34
P2	-0.25	-0.37	-0.24	-0.31	2.20	-0.33	-0.40	1.36	-0.39	-0.11	-0.17	-0.23	-0.31	-0.36	-0.21	-0.26	-0.16	0.80	0.19	-0.43
P1	-0.04	0.08	-0.45	0.08	-0.15	-0.12	0.00	-0.33	-0.12	-0.18	0.56	0.00	-0.02	2.36	-0.07	0.00	0.04	-0.36	-0.07	-0.27
P1'	0.91	-0.35	-0.10	-0.38	1.30	0.49	0.64	-0.05	-0.46	-0.26	0.58	-0.56	-0.41	-0.40	-0.50	1.69	-0.30	-0.35	2.37	1.85

TEV E107D, D127A, S135F, R203Q, K215E

	A	C	D	E	F	G	H	I	K	L	M	N	P	Q	R	S	T	V	W	Y
P6	-0.94	0.10	0.10	0.62	0.53	-0.01	-0.13	0.14	2.29	0.38	0.41	-0.11	-0.02	0.39	-0.04	-0.04	-0.08	0.14	0.00	0.44
P5	0.16	-0.39	-0.55	-0.44	0.12	-0.52	-0.44	0.51	0.36	0.46	0.68	-0.58	1.43	0.49	1.08	-0.37	0.19	0.33	0.24	-0.06
P4	-0.12	-0.07	0.56	-0.02	-0.20	-0.01	-0.18	1.05	0.02	0.94	0.03	0.06	-0.07	-0.06	-0.04	-0.13	-0.11	-0.06	0.00	-0.21
P3	0.00	-0.17	1.01	0.07	0.14	0.05	-0.10	-0.21	0.14	-0.02	-0.14	0.15	-0.12	-0.05	0.05	-0.09	-0.21	-0.18	0.14	0.35
P2	-0.12	-0.14	-0.10	-0.15	1.07	-0.24	-0.24	0.58	-0.11	-0.08	-0.18	-0.10	0.98	-0.17	0.01	-0.11	-0.13	0.27	0.07	-0.31
P1	-0.14	0.21	-0.49	-0.18	0.29	0.02	-0.18	-0.18	-0.07	0.12	0.13	-0.18	-0.03	0.76	-0.06	-0.11	-0.10	-0.24	0.60	-0.08
P1'	0.12	0.09	-0.27	-0.25	1.25	0.06	0.14	-0.21	-0.27	-0.15	0.14	-0.31	-0.27	-0.35	-0.30	0.72	-0.29	-0.35	1.50	1.24

TEV E107D, D127A, S135F

	A	C	D	E	F	G	H	I	K	L	M	N	P	Q	R	S	T	V	W	Y
P6	-0.81	0.25	0.57	2.22	1.16	-0.13	-0.08	0.90	2.59	1.14	0.94	-0.15	0.00	0.71	-0.14	-0.01	0.02	0.67	0.64	1.44
P5	0.15	-0.15	-0.08	0.20	0.42	-0.53	0.07	0.12	-0.18	0.27	0.47	-0.52	0.24	0.25	0.04	0.08	0.15	0.10	0.36	0.44
P4	-0.26	-0.14	0.80	-0.05	-0.20	-0.15	-0.27	3.37	-0.16	3.03	0.46	-0.02	-0.18	-0.29	-0.20	-0.25	-0.24	0.24	-0.03	-0.30
P3	-0.34	-0.40	-0.28	-0.27	0.73	0.75	-0.28	-0.15	-0.31	-0.25	-0.44	-0.16	-0.45	-0.32	-0.02	-0.41	-0.44	0.03	-0.12	2.57
P2	-0.37	-0.51	-0.54	-0.52	3.67	-0.55	-0.53	2.99	-0.56	0.09	0.03	-0.35	-0.18	-0.59	-0.41	-0.40	-0.22	2.52	1.22	-0.50
P1	-0.15	0.28	-0.47	-0.02	-0.11	-0.07	-0.02	-0.29	-0.09	-0.13	0.71	0.06	-0.07	2.88	-0.12	-0.03	-0.04	-0.36	0.05	-0.23
P1'	1.45	0.19	-0.04	-0.39	1.59	1.17	0.89	-0.27	-0.46	-0.35	1.25	-0.48	-0.56	-0.28	-0.46	2.27	-0.34	-0.49	1.89	2.40

Supplementary Table 12. Phage display enrichment values from selections on single site libraries. Each sub-table within the larger table represents the amino acid enrichment values generated for the given genotype of TEV protease. Each row contains enrichment values from a selection performed on the library in which the corresponding position within the ENLYFQS motif was randomized. The enrichment value for each amino acid identity at a given position was calculated as $frequency_{cleaved}/frequency_{elution}$-1. The cells are shaded on a linear scale from red to blue; this color scale is normalized for each sub-table with the lowest number in the sub-table being the darkest red and the highest number in the sub-table being darkest blue.

WT XXXYFQS	A	C	D	E	F	G	H	I	K	L	M	N	P	Q	R	S	T	V	W	Y
P6	-0.24	-0.53	1.34	2.27	0.83	-0.40	-0.10	0.30	-0.45	0.15	0.25	-0.31	-0.19	-0.27	-0.43	-0.01	-0.29	0.06	-0.15	1.28
P5	-0.13	-0.33	-0.23	-0.12	0.21	-0.50	-0.10	0.41	-0.35	0.86	0.12	-0.43	-0.34	0.09	-0.43	-0.30	-0.13	0.00	1.08	0.19
P4	-0.44	-0.37	-0.60	-0.61	-0.49	-0.56	-0.48	-0.12	-0.48	5.17	-0.42	-0.46	-0.50	-0.32	-0.39	-0.44	-0.49	-0.47	-0.36	-0.49
**WT EXXXFQS**	A	C	D	E	F	G	H	I	K	L	M	N	P	Q	R	S	T	V	W	Y
P5	-0.08	-0.44	0.54	0.30	-0.13	0.06	0.52	-0.17	-0.58	0.16	0.55	-0.68	0.22	-0.17	-0.21	-0.14	-0.43	-0.07	0.60	-0.38
P4	-0.69	-0.66	-0.55	-0.80	-0.69	-0.78	-0.63	2.73	-0.63	4.05	0.09	-0.70	-0.76	-0.73	-0.71	-0.73	-0.69	-0.49	-0.70	-0.72
P3	-0.73	-0.68	-0.59	-0.79	0.20	-0.76	-0.69	0.12	-0.75	-0.56	-0.66	-0.68	-0.82	-0.75	-0.67	-0.75	-0.76	-0.07	-0.56	5.51
**WT ENXXXQS**	A	C	D	E	F	G	H	I	K	L	M	N	P	Q	R	S	T	V	W	Y
P4	-0.32	-0.67	-0.94	-0.89	-0.67	-0.12	-0.78	0.46	-0.65	6.62	-0.14	-0.52	-0.77	-0.72	-0.73	-0.79	-0.66	-0.35	-0.59	-0.59
P3	-0.04	-0.66	-0.87	-0.14	0.31	-0.20	-0.84	-0.63	-0.69	-0.36	-0.50	-0.69	-0.85	-0.78	-0.53	-0.62	-0.71	-0.54	-0.12	2.91
P2	-0.82	-0.74	-0.93	-0.51	1.35	-0.62	-0.74	1.66	-0.81	0.03	-0.32	-0.78	-0.72	-0.74	-0.69	-0.59	-0.36	0.74	-0.50	-0.43
**WT ENLXXXS**	A	C	D	E	F	G	H	I	K	L	M	N	P	Q	R	S	T	V	W	Y
P3	-0.59	-0.47	-0.57	-0.66	-0.06	-0.53	-0.51	-0.50	-0.55	-0.42	-0.52	-0.64	-0.73	-0.62	-0.75	-0.70	-0.52	-0.45	-0.54	3.19
P2	-0.56	-0.29	-0.62	-0.71	-0.32	-0.49	-0.64	-0.37	-0.42	-0.19	-0.57	-0.72	-0.55	-0.69	-0.60	-0.05	-0.67	0.35	1.60	-0.33
P1	-0.57	-0.27	-0.61	-0.59	-0.54	-0.32	-0.25	-0.51	-0.51	-0.53	-0.26	1.29	-0.59	15.26	-0.55	-0.58	-0.56	-0.64	-0.68	-0.63
**WT ENLYXXX**	A	C	D	E	F	G	H	I	K	L	M	N	P	Q	R	S	T	V	W	Y
P2	-0.16	-0.46	-0.40	-0.27	1.83	0.55	-0.46	0.03	-0.65	-0.16	-0.27	-0.61	-0.63	-0.51	-0.51	-0.36	-0.39	0.70	-0.06	-0.29
P1	-0.31	-0.51	-0.43	-0.19	-0.47	-0.47	-0.30	-0.42	-0.59	-0.32	-0.25	-0.31	-0.63	11.84	-0.37	-0.43	-0.44	-0.42	-0.41	-0.39
P1'	0.78	-0.23	-0.56	-0.70	-0.47	0.41	1.40	-0.44	-0.43	-0.41	0.40	-0.11	-0.46	-0.15	-0.36	0.73	-0.45	-0.22	-0.06	-0.15

L2F XXXYFQS	A	C	D	E	F	G	H	I	K	L	M	N	P	Q	R	S	T	V	W	Y
P6	-0.23	-0.51	-0.16	-0.24	0.29	-0.20	0.05	-0.08	-0.34	0.31	0.03	-0.24	0.00	-0.30	-0.16	-0.03	0.09	0.06	0.26	-0.08
P5	-0.17	0.02	-0.17	-0.07	0.06	-0.44	-0.13	0.14	0.15	0.30	-0.35	-0.41	-0.33	-0.24	-0.08	-0.43	-0.21	0.18	0.86	0.82
P4	0.78	0.38	-0.81	-0.88	-0.61	-0.82	-0.87	3.19	-0.85	3.61	1.77	-0.87	-0.86	-0.88	-0.87	-0.69	-0.72	1.15	-0.76	-0.80
**L2F EXXXFQS**	A	C	D	E	F	G	H	I	K	L	M	N	P	Q	R	S	T	V	W	Y
P5	-0.08	0.31	0.43	0.18	0.07	0.05	-0.09	0.07	0.19	0.23	0.01	-0.77	0.05	-0.09	-0.05	-0.06	-0.39	-0.16	0.35	0.00
P4	-0.05	-0.23	-0.56	-0.78	-0.42	-0.75	-0.77	2.48	-0.76	3.20	0.75	-0.70	-0.76	-0.77	-0.76	-0.72	-0.67	-0.12	-0.51	-0.64
P3	-0.48	-0.49	-0.64	-0.55	0.22	-0.73	-0.53	0.61	-0.57	0.58	0.13	-0.64	-0.71	-0.38	-0.48	-0.64	-0.45	0.35	0.22	0.40
**L2F ENXXXQS**	A	C	D	E	F	G	H	I	K	L	M	N	P	Q	R	S	T	V	W	Y
P4	-0.48	-0.35	-0.88	-0.77	-0.05	-0.28	-0.81	2.31	-0.30	5.44	-0.30	-0.61	-0.65	-0.77	-0.77	-0.69	-0.72	-0.67	-0.47	-0.72
P3	-0.52	-0.57	-0.81	-0.35	-0.11	-0.35	-0.84	-0.13	-0.67	0.77	0.33	-0.52	-0.15	-0.54	-0.64	-0.48	-0.25	0.18	0.09	0.34
P2	-0.68	-0.45	-0.82	-0.61	1.41	0.18	-0.81	-0.24	-0.94	0.18	-0.60	-0.81	-0.26	-0.46	-0.59	0.15	-0.53	-0.17	0.54	-0.01
**L2F ENLXXXS**	A	C	D	E	F	G	H	I	K	L	M	N	P	Q	R	S	T	V	W	Y
P3	-0.73	-0.68	-0.67	-0.77	0.15	-0.70	-0.67	0.54	-0.54	0.52	-0.41	-0.74	-0.78	-0.41	-0.77	-0.77	-0.65	0.09	-0.25	0.97
P2	0.44	-0.54	-0.64	-0.71	1.06	-0.09	-0.78	0.04	-0.66	-0.30	-0.73	-0.82	-0.61	-0.78	-0.78	-0.40	-0.61	-0.08	0.81	0.23
P1	-0.35	-0.59	-0.71	0.92	0.74	-0.61	3.94	-0.77	-0.75	-0.49	2.88	1.81	-0.66	2.82	-0.63	-0.68	-0.53	-0.79	-0.74	0.27
**L2F ENLYXXX**	A	C	D	E	F	G	H	I	K	L	M	N	P	Q	R	S	T	V	W	Y
P2	0.14	-0.29	-0.50	-0.79	2.09	-0.39	-0.72	-0.53	-0.76	-0.16	-0.53	-0.68	-0.35	-0.69	-0.72	-0.40	-0.35	0.17	1.11	0.11
P1	-0.66	-0.67	-0.62	0.52	0.13	-0.58	2.90	-0.71	-0.71	-0.54	2.37	0.39	-0.71	2.81	-0.64	-0.70	-0.29	-0.74	-0.66	-0.02
P1'	0.51	-0.29	-0.51	-0.54	-0.22	0.18	0.40	-0.29	-0.74	0.04	1.04	-0.14	-0.75	-0.46	-0.67	0.68	-0.24	0.37	1.10	-0.41

L2F XXXVGHM	A	C	D	E	F	G	H	I	K	L	M	N	P	Q	R	S	T	V	W	Y
P6	-0.09	-0.21	-0.44	4.48	-0.23	-0.09	0.06	-0.01	0.17	-0.12	0.23	0.10	0.05	-0.28	-0.10	-0.16	0.01	0.03	0.02	0.26
P5	-0.01	-0.11	-0.11	0.05	0.38	-0.30	-0.16	0.00	1.03	0.06	-0.21	0.31	-0.43	0.03	-0.21	-0.18	-0.36	-0.03	0.12	0.25
P4	-0.14	0.13	-0.38	-0.47	-0.12	-0.16	-0.09	-0.19	0.17	1.12	-0.35	-0.23	0.18	-0.44	-0.15	-0.31	-0.20	-0.13	-0.10	-0.38
**L2F HXXXGHM**	A	C	D	E	F	G	H	I	K	L	M	N	P	Q	R	S	T	V	W	Y
P5	-0.12	0.32	0.18	-0.48	0.21	-0.55	-0.19	0.17	-0.50	-0.16	-0.20	-0.07	-0.17	-0.03	-0.38	-0.36	-0.34	-0.15	0.41	-0.01
P4	-0.32	-0.11	-0.69	-0.70	-0.15	-0.22	-0.32	0.03	-0.24	1.28	0.02	-0.10	-0.14	-0.34	-0.37	-0.05	-0.42	0.00	-0.21	-0.20
P3	-0.30	-0.18	-0.28	-0.12	0.16	-0.49	-0.17	0.66	-0.22	0.33	0.19	-0.25	-0.62	-0.13	-0.36	-0.34	-0.28	0.08	0.02	0.16
**L2F HPXXXHM**	A	C	D	E	F	G	H	I	K	L	M	N	P	Q	R	S	T	V	W	Y
P4	-0.87	-0.84	-0.91	-0.91	-0.82	-0.88	-0.91	1.28	-0.85	6.50	-0.83	-0.94	-0.86	-0.88	-0.83	-0.85	-0.89	-0.83	-0.83	-0.87
P3	-0.90	-0.76	-0.23	-0.82	-0.49	-0.88	-0.87	1.92	-0.85	0.64	-0.18	-0.85	-0.87	-0.70	-0.85	-0.89	-0.82	0.99	-0.74	1.37
P2	-0.08	-0.28	-0.75	-0.78	2.00	-0.77	-0.86	-0.78	-0.91	-0.09	-0.84	-0.86	-0.87	-0.88	-0.89	-0.66	-0.52	0.73	0.71	-0.89
**L2F HPLXXXM**	A	C	D	E	F	G	H	I	K	L	M	N	P	Q	R	S	T	V	W	Y
P3	-0.70	-0.60	-0.49	-0.69	0.03	-0.78	-0.72	0.18	-0.76	2.63	-0.40	-0.69	-0.74	-0.70	-0.72	-0.76	-0.70	-0.04	0.28	0.27
P2	0.01	-0.55	-0.45	-0.72	2.43	-0.69	-0.69	-0.12	-0.72	-0.26	-0.68	-0.71	-0.71	-0.76	-0.75	-0.48	-0.56	0.49	0.71	0.60
P1	-0.02	-0.56	-0.71	-0.65	0.83	-0.80	4.96	-0.65	-0.72	-0.62	0.38	-0.21	-0.78	2.19	-0.75	-0.52	-0.47	-0.62	-0.24	-0.43
**L2F HPLVXXX**	A	C	D	E	F	G	H	I	K	L	M	N	P	Q	R	S	T	V	W	Y
P2	0.04	0.02	-0.47	-0.45	0.83	-0.51	-0.46	-0.34	-0.43	0.06	-0.29	-0.45	-0.41	-0.38	-0.42	-0.46	-0.45	-0.17	1.46	0.00
P1	-0.34	-0.22	-0.41	-0.21	0.22	-0.49	5.04	-0.26	-0.50	-0.24	1.19	0.01	-0.44	-0.13	-0.44	-0.47	-0.38	-0.39	-0.40	-0.03
P1'	0.36	-0.17	-0.32	-0.36	-0.20	0.10	-0.27	0.28	-0.49	0.17	0.37	-0.18	-0.42	-0.30	-0.44	0.01	-0.10	0.64	0.54	0.02

Supplementary Table 13. Phage display enrichment values from selections on libraries with three randomized residues. Each sub-table within the larger table represents the amino acid enrichment values generated for the given genotype of TEV protease on the specified library (with randomized residues denoted as X). Each set of three rows contains enrichment values after two rounds of selection performed on the library in which the corresponding three positions within either the ENLYFQS or HPLVGHM motif was randomized. The enrichment value for each amino acid identity at a given position was calculated as $frequency_{cleaved}/frequency_{control\_selection}-1$. The cells are shaded on a linear scale from red to blue; this color scale is normalized for each sub-table with the lowest number in the sub-table being the darkest red and the highest number in the sub-table being darkest blue.

Shorthand Name	Type	Glycerol Stock	Origin of Replication	Resistance Marker	Description/Features
MP6	MP	MSP513	cloDF	Chloramphenicol	pBad dnaQ926, dam, seqA, emrR, ugi, cda1
122-1182-proB	AP	MSP955	pSC101	Carbenicillin	proB-lysozyme-ggs-ENLYFQS-ggs-T7RNAP//T7pro-gIII-lux
122-432-proB	AP	MSP565	pSC101	Carbenicillin	proB-lysozyme-ggs-HNLYFQS-ggs-T7RNAP//T7pro-gIII-lux
122-653-proB	AP	MSP722	pSC101	Carbenicillin	proB-lysozyme-ggs-ENLYGQS-ggs-T7RNAP//T7pro-gIII-lux
122-683-proB	AP	MSP770	pSC101	Carbenicillin	proB-lysozyme-ggs-HNLYFHS-ggs-T7RNAP//T7pro-gIII-lux
122-690-proB	AP	MSP780	pSC101	Carbenicillin	proB-lysozyme-ggs-HNLYGHS-ggs-T7RNAP//T7pro-gIII-lux
122-699-proB	AP	MSP794	pSC101	Carbenicillin	proB-lysozyme-ggs-HNLVGHS-ggs-T7RNAP//T7pro-gIII-lux
122-692-proB	AP	MSP782	pSC101	Carbenicillin	proB-lysozyme-ggs-HPLVGHM-ggs-T7RNAP//T7pro-gIII-lux
122-692-proA	AP	MSP814	pSC101	Carbenicillin	proA-lysozyme-ggs-HPLVGHM-ggs-T7RNAP//T7pro-gIII-lux
122-692-proB Q649S	AP	MSP832	pSC101	Carbenicillin	proB-lysozyme-ggs-HPLVGHM-ggs-T7RNAP(Q649S)//T7pro-gIII-lux
122-733-proB	AP	MSP833	pSC101	Carbenicillin	proB-lysozyme-IL-23(38-66)-T7RNAP//T7pro-gIII-lux
122-733-proB Q649S	AP	MSP855	pSC101	Carbenicillin	proB-lysozyme-IL-23(38-66)-T7RNAP(Q649S)//T7pro-gIII-lux
122-733-proA Q649S	AP	MSP848	pSC101	Carbenicillin	proA-lysozyme-IL-23(38-66)-T7RNAP(Q649S)//T7pro-gIII-lux
pET MBPTEV WT	Expression	MSP573	pBR322	Kanamycin	pET28 MBP-ENLYFQS-TEV WT
pET MBPTEV 111215 L1F	Expression	MSP850	pBR322	Kanamycin	pET28 MBP-HPLVGHM-TEV L1F 111215
pET MBPTEV 111215 L2F	Expression	MSP851	pBR322	Kanamycin	pET28 MBP-HPLVGHM-TEV L2F 111215
pET MBPTEV 111215 L5B	Expression	MSP852	pBR322	Kanamycin	pET28 MBP-HPLVGHM-TEV L5B 111215
pET MBPTEV H28L T30A	Expression	MSP968	pBR322	Kanamycin	pET28 MBP-ENLYFQM-TEV H28L T30A
pET MBPTEV HisP6c	Expression	MSP577	pBR322	Kanamycin	pET28 MBP-HNLYFQS-TEV I138T, N171D, N176T
pET MBPTEV HisP1a	Expression	MSP969	pBR322	Kanamycin	pET28 MBP-ENLYFHS-TEV T146S, D148P, S153N, S170A, N177M
pET MBPTEV GlyP2a	Expression	MSP824	pBR322	Kanamycin	pET28 MBP-ENLYGQS-TEV V209M, W211I, M218F
pET MBPTEV solC	Expression	MSP970	pBR322	Kanamycin	pET28 MBP-ENLYFQS-TEV E107D, D127A, S135F
pET MBPTEV solB	Expression	MSP971	pBR322	Kanamycin	pET28 MBP-ENLYFQS-TEV E107D, D127A, S135F, R203Q, K215E
pET MBPTEV solA	Expression	MSP972	pBR322	Kanamycin	pET28 MBP-ENLYFQS-TEV T17S, N68D, E107D, D127A, F132L, S135F, F162S, K229E
pET MBPGST 447	Expression	MSP574	pBR322	Kanamycin	pET28 MBP-ENLYFQS-GST
pET MBPGST 446	Expression	MSP578	pBR322	Kanamycin	pET28 MBP-HNLYFQS-GST
pET MBPGST 709	Expression	MSP805	pBR322	Kanamycin	pET28 MBP-ENLYFHS-GST
pET MBPGST 710	Expression	MSP806	pBR322	Kanamycin	pET28 MBP-ENLYgQS-GST
pET MBPGST 711	Expression	MSP807	pBR322	Kanamycin	pET28 MBP-HNLYFHS-GST
pET MBPGST 712	Expression	MSP808	pBR322	Kanamycin	pET28 MBP-HNLYGHS-GST
pET MBPGST 713	Expression	MSP809	pBR322	Kanamycin	pET28 MBP-HPLVGHM-GST

Supplementary Table 14. Plasmids used for PACE and protein expression. Plasmids are listed with important features including origin of replication, resistance marker, and encoded proteins.

Name	Sequence	Purpose
MSP693	5'AGATGGGTTCATTGUTGGTATANNKTCAGCATCGAATTTCACCAACACAATTNNKTATTTCACAAGCGTGCCGAAAAAC3'	Cloning P1 TEV SP library
MSP694	5'ACAATGAACCCATCUCTAGTTGATACTAATGGACTGCCACACTGCCCMNNCTTMNNTTGAATCCAATGCTTCCAGAA3'	Cloning P1 TEV SP library
MSP695	5'AGATGGGTTCATTGUTGGTATANNKTCAGCATCGGATTTCACCAACACAACTNNKTATTTCACAAGCGTGCCGAAAAAC3'	Cloning P1 TEV SP library
MSP620	5'AATGCTGACTCUNNKTTGNNKGGGGGCCATAAA3'	Cloning P2 TEV SP library
MSP621	5'AGGCTCTTCAGGTTUCACMNNGAAMNNTTTATGGCCCCC3'	Cloning P2 TEV SP library
MSP622	5'AAACCTGAAGAGCCUTTTCAGCCAGTTAAG3'	Cloning P2 TEV SP library
MSP454	5'AGTCAGCAUTTAATCGCCAACCACTAACCCAC3'	Cloning P2 TEV SP library
MSP441	5'ACTCAGCAUCGNNKTTCACCAACACANNKAATNNKTTCACAAGCGTGCCGAAAAAC3'	Cloning P6 TEV SP library
MSP442	5'ATGCTGAGUGTATACCAACAATGAACCCATC3'	Cloning P6 TEV SP library
MSP745	5'AGAACCACCTTTGUCGTCGTCGTCTTTGTAGTCGGAGTGAGAATAGAAAGGAACAACTAA3'	Universal substrate phage library primer
MSP809	5'ACAAAGGTGGTTCUNNKNNKNNKTACTTCCAGTCTGGTGGTTCTGCTGAAACTGTTGAAA3'	Cloning substrate phage library: xxxYFQS
MSP810	5'ACAAAGGTGGTTCUGAANNKNNKNNKTTCCAGTCTGGTGGTTCTGCTGAAACTGTTGAAA3'	Cloning substrate phage library: ExxxFQS
MSP811	5'ACAAAGGTGGTTCUGAAAACNNKNNKNNKCAGTCTGGTGGTTCTGCTGAAACTGTTGAAA3'	Cloning substrate phage library: ENxxxQS
MSP812	5'ACAAAGGTGGTTCUGAAAACCTGNNKNNKNNKTCTGGTGGTTCTGCTGAAACTGTTGAAA3'	Cloning substrate phage library: ENLxxxS
MSP813	5'ACAAAGGTGGTTCUGAAAACCTGTACNNKNNKNNKGGTGGTTCTGCTGAAACTGTTGAAA3'	Cloning substrate phage library: ENLYxxx
MSP814	5'ACAAAGGTGGTTCUNNKNNKNNKGTCGGCCACATGGGTGGTTCTGCTGAAACTGTTGAAA3'	Cloning substrate phage library: xxxVGHM
MSP815	5'ACAAAGGTGGTTCUCACNNKNNKNNKGGCCACATGGGTGGTTCTGCTGAAACTGTTGAAA3'	Cloning substrate phage library: HxxxGHM
MSP816	5'ACAAAGGTGGTTCUCACCCCNNKNNKNNKCACATGGGTGGTTCTGCTGAAACTGTTGAAA3'	Cloning substrate phage library: HPxxxHM
MSP817	5'ACAAAGGTGGTTCUCACCCCCTGNNKNNKNNKATGGGTGGTTCTGCTGAAACTGTTGAAA3'	Cloning substrate phage library: HPLxxxM
MSP818	5'ACAAAGGTGGTTCUCACCCCCTGGTCNNKNNKNNKGGTGGTTCTGCTGAAACTGTTGAAA3'	Cloning substrate phage library: HPLVxxx
MSP845	5'ACAAAGGTGGTTCUNNKAACCTGTACTTCCAGTCTGGTGGTTCTGCTGAAACTGTTGAAA3'	Cloning substrate phage library: xNLYFQS
MSP846	5'ACAAAGGTGGTTCUGAANNKCTGTACTTCCAGTCTGGTGGTTCTGCTGAAACTGTTGAAA3'	Cloning substrate phage library: ExLYFQS
MSP847	5'ACAAAGGTGGTTCUGAAAACNNKTACTTCCAGTCTGGTGGTTCTGCTGAAACTGTTGAAA3'	Cloning substrate phage library: ENxYFQS
MSP848	5'ACAAAGGTGGTTCUGAAAACCTGNNKTTCCAGTCTGGTGGTTCTGCTGAAACTGTTGAAA3'	Cloning substrate phage library: ENLxFQS
MSP849	5'ACAAAGGTGGTTCUGAAAACCTGTACNNKCAGTCTGGTGGTTCTGCTGAAACTGTTGAAA3'	Cloning substrate phage library: ENLYxQS
MSP850	5'ACAAAGGTGGTTCUGAAAACCTGTACTTCNNKTCTGGTGGTTCTGCTGAAACTGTTGAAA3'	Cloning substrate phage library: ENLYFxS
MSP851	5'ACAAAGGTGGTTCUGAAAACCTGTACTTCCAGNNKGGTGGTTCTGCTGAAACTGTTGAAA3'	Cloning substrate phage library: ENLYFQx
BCD1136	5’GGAATACCCAAAAGAACTGGCATG3’	Sanger sequencing of TEV SP
MSP819	5’ACACTCTTTCCCTACACGACGCTCTTCCGATCTNNNNCAACAGTTTCAGCAGAACCAC3’	PCR for HT-Seq of substrate phage
MSP820	5’ACACTCTTTCCCTACACGACGCTCTTCCGATCTNNNNAACAGTTTCAGCAGAACCACC3’	PCR for HT-Seq of substrate phage
MSP824	5’TGGAGTTCAGACGTGTGCTCTTCCGATCTGTTCCTTTCTATTCTCACTCCGAC3’	PCR for HT-Seq of substrate phage

Supplementary Table 15. Primer Sequences

Supplementary Note 1. MATLAB script for extracellular target substrate search.

```
%We iterated through each entry in ProteinList,
%which is the list of extracellular protein amino acid sequences
%(corresponding gene names are stored in ExtracellularNames)
for i=1:length(ProteinList)
   aa=ProteinList{i};
   %convert amino acid letters to a sequence of integers
   protein=aa2int(aa);
   %check that there are only the 20 canonical amino acids
   if sum(protein>20)==0 && sum(protein<=0)==0
      %initialize and empty score output
      SpecificityScore=[];
      %convert integer protein code to a sparse matrix
      proteinmat=sparse([1:length(protein)], double(protein),ones(1,length(protein)),length(protein),20);
      %for every window of seven amino acids we calculate a match score
      %(SpecificityScore) by multiplying the sparse protein sequence matrix
      %by the scoring matrix (TEVspecificity) and sum the diagonal by taking the trace of the product
      for j=1:length(protein)-6
         SpecificityScore=[SpecificityScore; trace(proteinmat(j:j+6,:)*TEVspecificity)];
      end
      %find the max score and the index to locate the substrate sequence
      [C,I]=max(SpecificityScore);
      score(i,1)=C;
      %store the corresponding best match peptide in aligns
      aligns(i,1:7)=aa(I:I+6);
      starts(i)=I;
   end
end
%sort the best matches for each protein by score
[score,I]=sort(score,'descend');
aligns=aligns(I,1:7);
hits=ExtracellularNames(I);
starts=starts(I);
```

Supplementary Note 2. Python script for processing high-throughput sequencing data from phage substrate display experiments.

In [1]:

```
%matplotlib inline
import numpy as np
import scipy as sp
import matplotlib as mpl
import matplotlib.cm as cm
import matplotlib.pyplot as plt
import pandas as pd
pd.set_option('display.width', 500)
pd.set_option('display.max_columns', 100)
pd.set_option('display.notebook_repr_html', True)
import seaborn as sns
sns.set_style("whitegrid")
sns.set_context("poster")
import requests
import time
from bs4 import BeautifulSoup
import regex
import re
import os
from Bio import SeqIO
import Bio
from Bio import motifs
from Bio.Alphabet import IUPAC, Gapped
alpha = Gapped(IUPAC.protein)
```

In [2]:

```
#specify input and output directories; iterate through fastq files
indir='/Users/michaelpacker/Desktop/Liu_Lab/MiSeqData/021816Miseq/fastq/'
outdir='/Users/michaelpacker/Desktop/Liu_Lab/MiSeqData/021816Miseq/'
filenames=os.listdir(indir)
for i in range(len(filenames)):
    seqs={}
    #check that the file is fastq
    if filenames[i][-5:]=='fastq':
        #read fastq
```

```
        for record in SeqIO.parse(indir+filenames[i], "fastq") :
            #trimming to protease substrate
            #split on sequence immediately before protease substrate check that there are two entries in the split string
            if len(record.seq.tostring().split('AGAACCACC'))>=2:
                #take the second string from the first split, and split again on the sequence immediately after the protease
substrate
                sequence=record.seq.tostring().split('AGAACCACC')[1]
                #check substrate length
                if len(sequence)>=21:
                    seqs[record.id]=sequence[0:21]
        #only save substrates free of stop codons and amibiguous bases, filter out library cloning template sequence
HNLYGHS and display truncation sequence YKDDDDK
        substrates=[Bio.Seq.translate(Bio.Seq.reverse_complement(x)) for x in seqs.values() if '*' not in
Bio.Seq.translate(Bio.Seq.reverse_complement(x)) if
Bio.Seq.translate(Bio.Seq.reverse_complement(x))!='HNLYGHS'if
Bio.Seq.translate(Bio.Seq.reverse_complement(x))!='YKDDDDK'if 'X' not in
Bio.Seq.translate(Bio.Seq.reverse_complement(x))]
        #use motifs to calculate normalized amino acid frequencies
        M=motifs.create(substrates, alphabet=alpha)
        pd.DataFrame(substrates).to_csv(outdir+filenames[i]+'substrates.csv')
        pd.DataFrame(M.counts).to_csv(outdir+filenames[i]+'counts.csv')
pd.DataFrame(M.counts.normalize()).to_csv(outdir+filenames[i]+'normalizedcounts.csv')
```
